# The genome of the zoonotic malaria parasite *Plasmodium simium* reveals adaptations to host switching

**DOI:** 10.1186/s12915-021-01139-5

**Published:** 2021-10-01

**Authors:** Tobias Mourier, Denise Anete Madureira de Alvarenga, Abhinav Kaushik, Anielle de Pina-Costa, Olga Douvropoulou, Qingtian Guan, Francisco J. Guzmán-Vega, Sarah Forrester, Filipe Vieira Santos de Abreu, Cesare Bianco Júnior, Julio Cesar de Souza Junior, Silvia Bahadian Moreira, Zelinda Maria Braga Hirano, Alcides Pissinatti, Maria de Fátima Ferreira-da-Cruz, Ricardo Lourenço de Oliveira, Stefan T. Arold, Daniel C. Jeffares, Patrícia Brasil, Cristiana Ferreira Alves de Brito, Richard Culleton, Cláudio Tadeu Daniel-Ribeiro, Arnab Pain

**Affiliations:** 1 Pathogen Genomics Laboratory, Biological and Environmental Sciences and Engineering (BESE) Division, King Abdullah University of Science and Technology (KAUST), Thuwal, Saudi Arabia; 2 Grupo de Pesquisa em Biologia Molecular e Imunologia da Malária, Instituto René Rachou, Fundação Oswaldo Cruz (Fiocruz), Belo Horizonte, MG 30190-009 Brazil; 3 Centro de Pesquisa, Diagnóstico e Treinamento em Malária (CPD-Mal), Fiocruz, Rio de Janeiro, RJ 21040-360 Brazil; 4 Laboratório de Pesquisa Clínica em Doenças Febris Agudas, Instituto Nacional de Infectologia Evandro Chagas, Fiocruz, Rio de Janeiro, RJ 21040-360 Brazil; 5 Centro Universitário Serra dos Órgãos (UNIFESO), Teresópolis, RJ 25964-004 Brazil; 6 Computational Bioscience Research Center, Biological and Environmental Sciences and Engineering (BESE) Division, King Abdullah University of Science and Technology (KAUST), Thuwal, Saudi Arabia; 7 Department of Biology and York Biomedical Research Institute, University of York, Wentworth Way, York, YO10 5DD UK; 8 Laboratório de Mosquitos Transmissores de Hematozoários, Instituto Oswaldo Cruz (IOC), Fiocruz, Rio de Janeiro, RJ 21040-360 Brazil; 9 Laboratório de Pesquisa em Malária, IOC, Fiocruz, Rio de Janeiro, RJ 21040-360 Brazil; 10 Universidade Regional de Blumenau (FURB), Centro de Pesquisas Biológicas de Indaial (CEPESBI)/ Projeto bugio, Blumenau, Indaial, SC Brazil; 11 Centro de Primatologia do Rio de Janeiro (CPRJ/Inea), Guapimirim, RJ 25940-000 Brazil; 12 Centre de Biologie Structurale, CNRS, INSERM, Université de Montpellier, 34090 Montpellier, France; 13 Division of Molecular Parasitology, Proteo-Science Center, Ehime University, Toon, Ehime 791-0295 Japan; 14 Global Station for Zoonosis Control, Global Institution for Collaborative Research and Education (GI-CoRE), Hokkaido University, N20 W10 Kita-ku, Sapporo, Japan

**Keywords:** *Plasmodium simium*, *Plasmodium vivax*, Malaria, Zoonosis, Comparative genomics

## Abstract

**Background:**

*Plasmodium simium*, a malaria parasite of non-human primates (NHP), was recently shown to cause zoonotic infections in humans in Brazil. We sequenced the *P. simium* genome to investigate its evolutionary history and to identify any genetic adaptions that may underlie the ability of this parasite to switch between host species.

**Results:**

Phylogenetic analyses based on whole genome sequences of *P. simium* from humans and NHPs reveals that *P. simium* is monophyletic within the broader diversity of South American *Plasmodium vivax*, suggesting *P. simium* first infected NHPs as a result of a host switch of *P. vivax* from humans. The *P. simium* isolates show the closest relationship to Mexican *P. vivax* isolates. Analysis of erythrocyte invasion genes reveals differences between *P. vivax* and *P. simium*, including large deletions in the Duffy-binding protein 1 (DBP1) and reticulocyte-binding protein 2a genes of *P. simium*. Analysis of *P. simium* isolated from NHPs and humans revealed a deletion of 38 amino acids in DBP1 present in all human-derived isolates, whereas NHP isolates were multi-allelic.

**Conclusions:**

Analysis of the *P. simium* genome confirmed a close phylogenetic relationship between *P. simium* and *P. vivax*, and suggests a very recent American origin for *P. simium.* The presence of the DBP1 deletion in all human-derived isolates tested suggests that this deletion, in combination with other genetic changes in *P. simium*, may facilitate the invasion of human red blood cells and may explain, at least in part, the basis of the recent zoonotic infections.

**Supplementary Information:**

The online version contains supplementary material available at 10.1186/s12915-021-01139-5.

## Background

There are currently eight species of malaria parasites known to cause disease in humans: *Plasmodium falciparum*, *Plasmodium vivax*, *Plasmodium malariae* (considered to be indistinguishable from *P. brasilianum* [1]), *Plasmodium ovale curtisi*, *Plasmodium ovale wallikeri*, *Plasmodium knowlesi*, *Plasmodium cynomolgi* and *Plasmodium simium*. The last three species are non-human primate (NHP) parasites that have recently been shown to infect humans [2–4].

As interventions against human parasites, particularly *P. falciparum* and *P. vivax*, reduce their prevalence, the importance of zoonotic malaria is becoming increasingly apparent. In countries moving towards malaria elimination, the presence of potentially zoonotic parasite populations in NHPs is a significant obstacle.

The propensity of malaria parasites to switch hosts and the consequences of this for human health are highlighted by the fact that both *P. vivax* and *P. falciparum* first arose as human pathogens following host switches from non-human great apes in Africa [5–7]. Contact between humans and the mosquitoes that feed on NHPs is increasing due to habitat destruction and human encroachment into NHP habitats [8]. Therefore, there is increasing danger of zoonotic transmission and the emergence of novel human malaria pathogens. Understanding how malaria parasites adapt to new hosts and new transmission environments allows the risks posed by novel zoonotic malaria outbreaks to be assessed.

The clinical epidemiology of zoonotic malaria varies according to the parasite species and the demographics of the human host population. Severe and fatal outcomes for people infected with *P. knowlesi* in Malaysia have been reported [3], whilst *P. cynomolgi* infection in the same region causes moderate or mild clinical symptoms [9]. Interestingly, both *P. knowlesi* and *P. cynomolgi* infections in the Mekong region appear less virulent than in Malaysia and are often asymptomatic [4, 10]. This may be due to differences in the relative virulence of the circulating parasite strains and/or in the susceptibility of the local human populations. As NHP parasites have adapted to co-evolved with their natural hosts, it is impossible to predict their virulence in zoonotic infections. For example, the pathogenicity of *P. falciparum* has been attributed to its relatively recent emergence as a human pathogen [11], which is thought to have occurred following a single transfer from a gorilla in Africa [6].

Eighty-nine percent of malaria infections in Brazil are caused by *P. vivax* and over 99.9% of these cases occur in the Amazonian region. This region accounts for almost 60% of the area of Brazil and is home to 13% of the human population (https://www.ibge.gov.br/). Around 90% of the malaria cases registered outside the Amazonian region occur in the Atlantic Forest, a region of tropical forest that extends along Brazil’s Atlantic coast. The infections are apparently mild and are caused by a vivax-like malaria parasite transmitted by *Anopheles (Kerteszia) cruzii*, a mosquito species that breeds in the leaf axils of bromeliad plants [12].

A malaria outbreak in the Atlantic Forest of Rio de Janeiro in 2015/2016 was shown to be caused by the NHP parasite *P. simium* [2]. Parasite DNA samples collected from both humans and NHPs in the same region had identical mitochondrial genome sequences, distinct from that of *P. vivax* isolates collected anywhere in the world but identical to that of a *P. simium* parasite isolated from a monkey in the same region in 1966, and to all subsequent *P. simium* isolates recovered from NHPs [13, 14].

Previously, it was thought that *P. vivax* became a human parasite following a host switch from macaques in Southeast Asia, due to its close phylogenetic relationship with a clade of parasites infecting macaques in this region, and due to the high genetic diversity among *P. vivax* isolates from Southeast Asia [15]. However, it is now considered likely that it became a human parasite following a host switch from non-human great apes in Africa [7]. It seems likely that *P. vivax* was introduced to the Americas by European colonisers following Columbus’ journey to the New World towards the end of the fifteenth century, since present-day South American *P. vivax* is closely related to a strain of the parasite present, historically, in Spain [16]. However, there is some evidence to suggest that *P. vivax* may also have been introduced to South America in pre-Columbian times [17], and this, together with the post-Columbian influx of infected people from various regions of the world associated with colonisation, may have contributed to the extensive genetic diversity of present-day *P. vivax* in Central and South America [17].

*Plasmodium simium*, a parasite of various species of Platyrrhini monkey whose range is restricted to the Atlantic Forest from Southeast and South of Brazil [18], is genetically, morphologically and immunologically similar to *P. vivax* [2, 19–22]. Based on this similarity, it is likely that the *P. simium* parasite of NHPs in Brazil originated from *P. vivax* following a host switch from humans. The recent 2015/2016 outbreak of *P. simium* in the local human population of Rio de Janeiro’s Atlantic Forest raises questions about the degree of divergence between *P. vivax* and *P. simium*, and whether adaptation to NHPs has led to the evolution of a parasite with relevance to human health that differs from that of *P. vivax*.

It remains unproven whether the current outbreak of *P. simium* in the human population of Rio de Janeiro was the result of a single parasite transfer from a NHP to a human and its subsequent transfer between people, or whether multiple independent transfers have occurred with different NHP-derived parasites. Furthermore, the nature and degree of adaptation to NHP hosts and a sylvatic transmission cycle that has occurred in *P. simium* following its anthroponotic origin is relevant to understanding how malaria parasites adapt to new hosts. It is of interest to determine whether the current, human-infecting *P. simium* parasites have undergone recent changes at the genomic level which allow them to infect humans, as it has been previously suggested that *P. simium* lacks this ability [23].

In order to address these questions, and so to better understand the epidemiology and natural history of this emerging zoonotic parasite, we analysed whole genome sequences of *P. simium* parasites isolated from both humans and NHPs.

## Results

### Genome assembly and phylogeny

From a single *P. simium* sample collected from Rio de Janeiro state in 2016 [2], short read DNA sequences were obtained and assembled into a draft genome. The assembled genome consists of 2192 scaffolds over 1 kb in length with a combined size of 29 Mb (Additional file 1: Table S1). Two scaffolds corresponding to the apicoplast and mitochondrial genomes were also identified (Additional file 2: Figure S1). Gene content analysis showed a completeness of annotation comparable to previously published *Plasmodium* assemblies (Additional file 2: Figure S2). Hence, although the fragmented nature of the chromosome assemblies prevents precise assessment of synteny conservation with other *Plasmodium* species, the set of gene components in the *P. simium* genome is relatively complete. A phylogenetic tree constructed from 3181 of 1:1 orthologs of the annotated *P. simium* protein-coding genes with the orthologues of *P. vivax*, *P. cynomolgi*, *P. coatneyi*, *P. knowlesi*, *P. malariae*, *P. falciparum*, *P. reichenowi* and *P. gallinaceum* confirmed that *P. simium* is very closely related to *P. vivax* (Additional file 2: Figure S3). Analysis of gene families revealed a *P. simium* gene repertoire largely similar to *P. vivax* (Additional file 1: Table S2; Additional file 2: Figure S4), although the number of *PIR* genes in *P. simium* genome appear to be less than half the number of *VIR* genes in *P. vivax*. This difference could stem from incomplete genome assembly, and the highly uneven read coverage between *PIR* genes suggests that a subset of reads stemming from *PIR* genes may often be mapped to an incorrect *PIR* locus, which is also likely to affect assembly of these redundant sequences (Additional file 2: Figure S4). There is no indication of a recent expansion of the *P. simium PIR* gene family (Additional file 2: Figure S5).

### *P. simium-P. vivax* diversity analysis

To detect single-nucleotide polymorphisms (SNPs) within the *P. vivax*/*P. simium* clade, short Illumina paired-end sequence reads were mapped onto the *P. vivax* P01 reference genome [24]. Reads were collected from eleven human-derived *P. simium* samples, two monkey *P. simium* samples, two *P. vivax* samples from the Brazilian Amazon, and a previously published *P. simium* CDC strain (originally isolated in 1966, see ‘Methods’). Data from a range of *P. vivax* strains representing a global distribution was retrieved from the literature [25]. Including only SNPs with a minimum depth of five reads, a total of 232,780 SNPs were called initially across 79 samples. Sixteen samples were subsequently removed, primarily due to low coverage, resulting in a total of 63 samples for further analysis (Additional file 1: Table S3, Table S4). Since few SNP loci were covered across all samples, and to enable diversity analysis, we restricted all further analysis to the 124,968 SNPs for which data were available from at least 55 samples (Additional file 2: Figure S6).

### *P. simium-P. vivax* population analysis

**Fig. 1:**
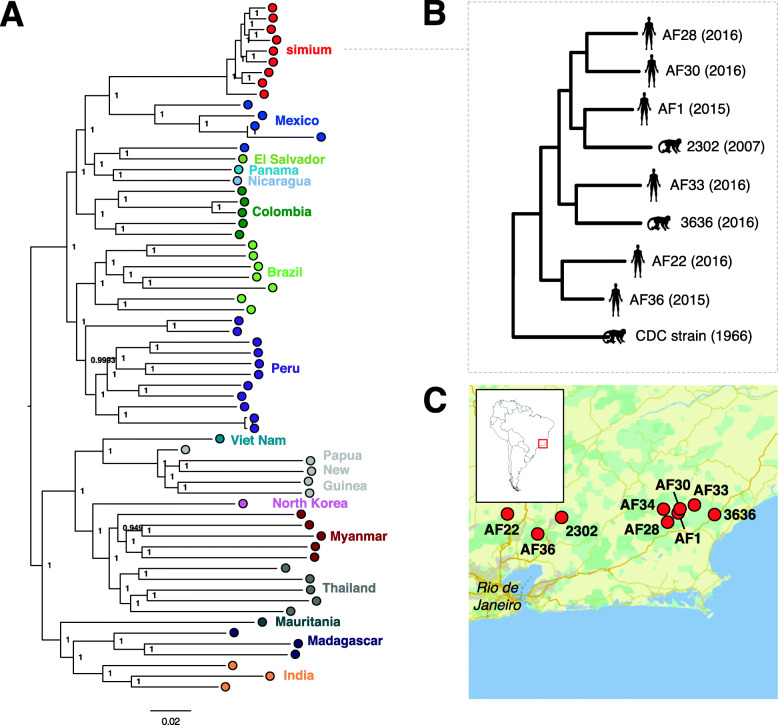
SNP phylogeny. **A** Mid-point rooted maximum likelihood tree produced from 143,123 concatenated SNP positions with data from a minimum of 55 samples. The tree was produced using PhyML with the GTR evolutionary model. Branch support was evaluated with the Bayesian-like transformation of approximate likelihood ratio test (aBayes). Genetic distance is shown below the tree. *Plasmodium vivax* isolates are denoted as coloured circles by their country of sample origin. A tree with specific sample IDs is available in Additional file 2: Figure S9B. **B** Magnification of the *Plasmodium simium* clade (as in **A**). **C** Map showing the geographic locations at which the *P. simium* samples were collected

A principal component analysis (PCA) plot constructed from these genome-wide SNP loci showed a clear separation between American and Asian *P. vivax* samples as well as a distinct grouping of *P. simium* samples (Additional file 2: Figure S7). The latter observation suggests that both human and NHP *P. simium* isolates represent a single population that is genetically different from American *P. vivax* populations. A similar pattern is observed by performing a multidimensional scaling analysis of the SNP data (Additional file 2: Figure S8). To enable a phylogenetic approach, we constructed an alignment from the 124,968 SNP sites. In the resulting phylogenetic tree, *P. vivax* strains generally clustered according to their geographical origin, with clear separation of the Asian and American samples (Fig. 1A, a tree with sample IDs is available in Additional file 2: Figure S9). *Plasmodium simium* samples clustered as a monophyletic group with Mexican *P. vivax* samples (Fig. 1A), consistent with a recent American origin for *P. simium.* This phylogeny, based on genome-wide SNPs, represents an ‘average’ phylogeny across the genome and cannot be considered to reflect a true history of parasite ancestry due to the effects of recombination. It is possible that trees produced from individual genes might reveal different phylogenetic relationships. Yet, the association between *P. simium* samples and *P. vivax* samples from Mexico is also observed in a phylogenetic network (Additional file 2: Figure S10).

To examine whether the *P. simium* isolates we studied were part of a continuous population with local *P. vivax* parasites, we examined population ancestry with the ADMIXTURE programme [26] (Additional file 2: Figure S11). This analysis is consistent with the PCA and MDS analyses (Additional file 2: Figure S7, Figure S8) and the phylogenetic analysis of segregating SNPs (Fig. 1), showing that *P. simium* forms a genetically distinct population separate from *P. vivax*. The absence of *P. simium-P. vivax* hybrids (indicating genetic introgression events) indicates that *P. simium* has undergone a period of independent evolution.

### Genetic diversity and population divergence

**Fig. 2:**
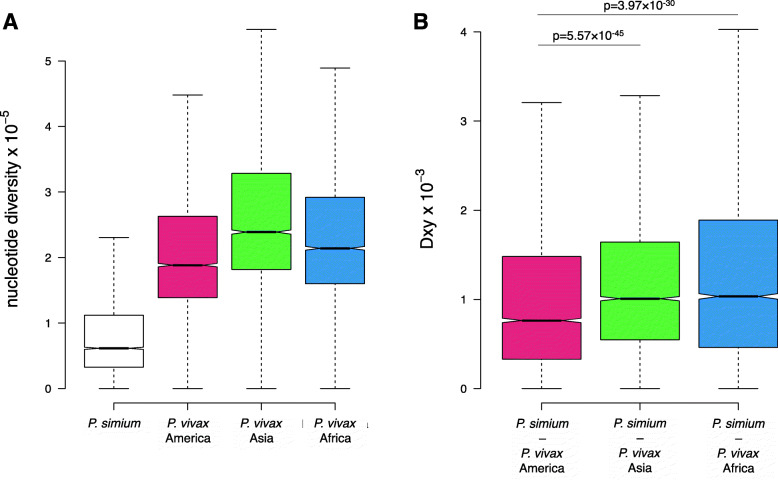
Nucleotide diversity and genetic distance between populations. **A** Diversity within populations. Box plot showing the nucleotide diversity in 10-kb windows between *Plasmodium simium* samples (left), and *Plasmodium vivax* samples from America, Asia and Africa. **B** Genetic distance between populations. Box plot showing *D*_XY_ in 10-kb windows comparing *Plasmodium simium* samples to *P. vivax* samples from America, Asia and Africa. *P* values from one-sided Mann-Whitney tests for difference in medians are shown above boxes. For both plots, boxes denote 25th and 75th percentiles with all outliers removed

To characterise the *P. simium* population further, we estimated the nucleotide diversity in *P. simium* and *P. vivax* samples (see ‘Methods’). *Plasmodium simium* diversity (genome-median: 1.3 × 10^−4^) is approximately six times lower than the diversity observed when comparing all *P. vivax* samples (genome-median: 7.9 × 10^−4^) (Fig. 2A). Diversity measured within coding sequences in *P. vivax* is consistent with previous reports [7]. The median nucleotide divergence between *P. simium* and *P. vivax* genomes of 8.7 × 10^−4^ and the low diversity within *P. simium* suggest that the strains we examined are part of a relatively recent or isolated population.

The difference in diversity within *P. simium* and *P. vivax* populations will influence relative measures of population differentiation such as *F*_ST_ [27], and we therefore calculated *D*_XY_, an absolute measure of diversity that is independent of the levels of diversity within the two populations being compared [27, 28]. When comparing *D*_XY_ in 10-kb windows across the genome, the diversity observed between *P. simium* samples and *P. vivax* samples from America is significantly lower than the diversity between *P. simium* and *P. vivax* populations from Asia and Africa (Fig. 2B), supporting the origin of *P. simium* from American *P. vivax* populations.

The vast majority of genes have an *D*_XY_ of zero, consistent with a recent split between *P. simium* and *P. vivax* (Additional file 2: Figure S12A). We noticed that genes showing the highest *D*_XY_ are from multi-gene families (Additional file 2: Figure S12B). Although polymorphic antigens are expected to display high levels of diversity, this could also be explained by the uncertainties in SNP calling among multi-gene families with high levels of sequence redundancy.

### *P. simium* red blood cell invadome components

Binding and red blood cell invasion is mediated by two key malaria gene families expressed in merozoites: the Duffy-binding proteins (DBPs) and the reticulocyte-binding proteins (RBPs). In *P. vivax*, DBPs bind to the Duffy antigen receptor for chemokines (DARC) [29, 30], which is present on both host normocytes and reticulocytes, and RBPs are known to restrict *P. vivax* to binding and invading reticulocytes [31–33]. Two DBPs, DBP1 and DBP2, are present in *P. vivax* P01 (Additional file 1: Table S5). Recently, the reported protein structure of *P. vivax* RBP2b revealed the conservation of residues involved in the invasion complex formation [33]. RBPs can be divided into three subfamilies, RBP1, RBP2 and RBP3 [34]. The *P. vivax* P01 genome encodes 11 RBPs (including the reticulocyte-binding surface protein, RBSA), of which three are pseudogenes (Additional file 1: Table S5).

**Fig. 3:**
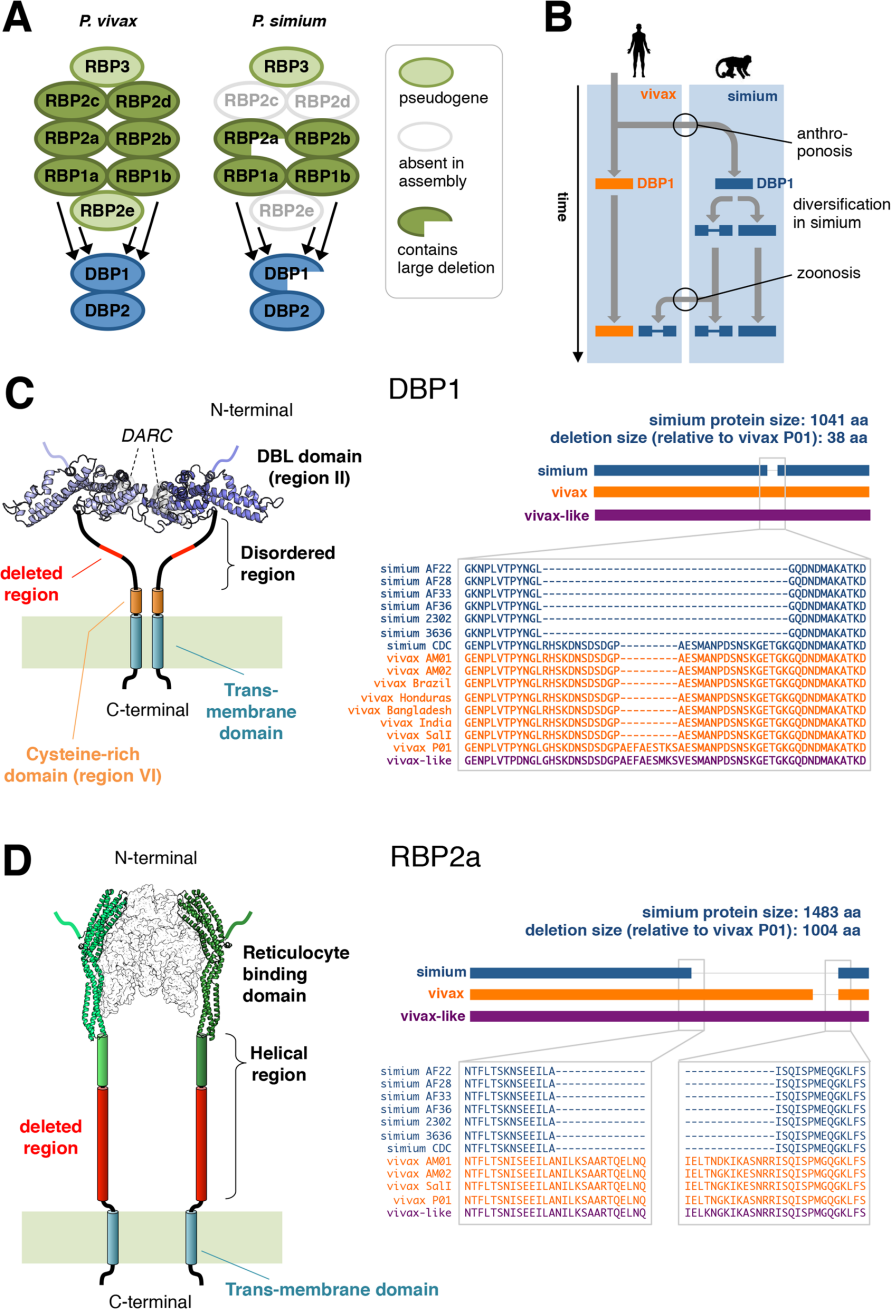
Red blood cell invadome deletions. **A** Overview of the red blood cell invadome gene groups, reticulocyte-binding proteins (RBPs) and Duffy-binding proteins (DBPs) in *Plasmodium vivax* and *Plasmodium simium*. The *P. vivax* genome harbours two RBP2d genes, one of which is a pseudogene (Additional file 1: Table S5). **B** Schematic depiction of the hypothesised scenario in which the DBP1 deletion—along with other accumulated genetic changes in *P. simium*—is a prerequisite for the recently observed zoonosis. **C** Left: Structural rendering of DBP1, showing known structural domains and motifs. The two fragment molecules from the human DARC receptor are shown in grey. The 3-dimensional structure of the DBL-DARC complex was modelled based on the *P. vivax* crystallographic model (PDB 4nuv). The region deleted in sequences from human-infecting *P. simium*, as compared to *P. vivax* P01, is highlighted in red. Right: Details of DBP1 protein alignments. A full alignment is available in Additional file 3: Figure S17. **D** Similar to panel **C** but for RBP2a. The complex between the reticulocyte-binding domain and the human receptor was modelled based on the cryoEM structure of the complex between the *P. vivax* RBP2b and the human transferrin receptor TfR1 (PDB 6d05). A full alignment is available in Additional file 4: Figure S25

The *P. vivax* DBP and RBP protein sequences were used to search for *P. simium* orthologues, resulting in the detection of the two DBP proteins and RBP1a, RBP1b, RBP2a, RBP2b and RBP3, and a failure to detect RBP2c and RBP2d (Fig. 3; Additional file 1: Table S5; Additional file 2: Figure S13, Figure S14) across all sequenced *P. simium* samples. As in *P. vivax* genomes, the *P. simium* RBP3 is a pseudogene [35], indicating that conversion to a pseudogene happened prior to the split between *P. vivax* and *P. simium*.

To determine whether the apparent absences of individual RBP genes in *P. simium* was due to incomplete genome assembly, we examined the coverage of *P. simium* reads mapped onto *P. vivax* RBP gene loci. No *P. simium* coverage was observed at the RBP2c, RBP2d and RBP2e genes in *P. simium* samples, including the previously published CDC strain (Additional file 2: Figure S15).

Coverage of mapped reads across red blood cell invadome-associated gene loci revealed no apparent elevated coverage in genes compared to their flanking genomic regions, which would have been expected if the *P. simium* genome contained multiple (duplicated) copies of non-assembled invasion genes (Additional file 2: Figure S16).

### Structural variation in *P. simium* Duffy-binding protein 1

The *P. simium* assembly revealed that the invasion gene DBP1 contains a large deletion within its coding sequence (Fig. 3; Additional file 3: Figure S17). Intriguingly, the previously published *P. simium* CDC strain DBP1 (GenBank accession: ACB42432) [36] does not contain this deletion (‘simium CDC’ in Fig. 3C). A haplotype network confirms that this previously published DBP1 gene is indeed a *P. simium* sequence (Additional file 2: Figure S18), and the SNP analyses consistently assign the CDC strain to the *P. simium* cluster (Fig. 1; Additional file 2: Figure S7, Figure S8). Compared to the *P. vivax* P01 reference genome, the SalI reference harbours a 27 base pair deletion in DBP1, in contrast to the 115 bp deletion observed in all *P. simium* samples isolated from humans (Fig. 3). The 27 base pair deletion is also present in most *P. vivax* isolates (Additional file 2: Figure S19). Additional deletion patterns exist among isolates, and in a few cases multiple versions are detected within samples (Additional file 2: Figure S19).

The presence of repetitive sequences within the DBP1 gene could potentially result in aberrant assembly across the DBP1 locus, which may appear as an apparent deletion in subsequent bioinformatic analysis. We tested this possibility and showed that the DBP1 gene does not harbour any noticeable degree of repetitiveness at the nucleotide level (Additional file 2: Figure S20). Several read mapping analyses confirmed that the *P. simium*-specific 115 bp deletion was not an assembly artefact (Additional file 2: Figures S21-S23).

We next designed primers for PCR amplification of a nucleotide sequence that spans the deleted region in the *P. simium* DBP1 gene and tested the occurrence of these deletion events in a range of *P. vivax* and *P. simium* field samples from Brazil. All *P. vivax* samples tested by PCR produced bands consistent with absence of the deletion whereas all samples from human-infecting *P. simium* produced bands consistent with the presence of the precise 115 bp deletion (Additional file 2: Figure S24, top & middle). Interestingly, NHP-infecting *P. simium* isolates contained a mix of sequences with and without deletions (Additional file 2: Figure S24, bottom). PCR results are summarised in Additional file 1: Table S6. Under the simplified assumption that the sampled NHP *P. simium* samples reflect the DBP1 allele frequency in the entire *P. simium* population (4 with deletion in DBP1, 8 without the deletion; Additional file 1: Table S6), the probability of randomly sampling 15 human *P. simium* samples all having the DBP1 deletion is 6.97 × 10^−8^ using a binomial distribution. If the *P. simium*-specific deletion in DBP1 is a prerequisite for the ability to infect humans, this suggests that only a subset of NHP-infecting *P. simium* parasites currently possess the ability to infect humans.

A large, 3012 bp additional deletion was observed in the *P. simium* RBP2a gene, the presence of which was also supported by read mapping (Fig. 3; Additional file 4: Figure S25; Additional file 2: Figures S26-S28). PCR-based genotyping results confirmed the presence of this deletion in all *P. simium* isolates irrespective of their host (Additional file 1: Table S6; Additional file 2: Figure S29).

To test for the presence of insertions and deletions (indels) in other protein-coding genes, we curated a set of indels by comparing short indels predicted by the DELLY software [37] to *P. simium* assemblies. This set consisted of 244 indels in 222 genes (Additional file 1: Table S7). Indel sizes were almost exclusively integers of three (hence conserving reading frames) and indels were predominantly found in low-complexity regions and in genes encoding long proteins (Additional file 2: Figure S30). Notably, short indels were recorded in 138 genes with known functional annotations with an overrepresentation of DNA-binding function (GO:0005488 ‘binding’, *p* = 0.0011; GO:0043565 ‘sequence-specific DNA binding’, *p* = 0.0062).

### Potential structural implications of the deletions in DBP1 and RBP2a

We next investigated whether the observed deletions render DBP1 and RBP2a non-functional. DBP1 contains a large extracellular region, which includes the N-terminal DBL region which mediates the association with DARC in *P. vivax* [38], followed by a largely disordered region and a cysteine-rich domain (Fig. 3C). DBP1 has a single-pass transmembrane helix and a short cytoplasmic tail. The deletion observed in the human-infecting *P. simium* only affects the disordered region, leaving the flanking domains intact. We produced homology models of the DBL domains from the *P. vivax* strain P01, the human-infecting *P. simium* strain AF22, and the *P. simium* CDC strain, based on the crystal structure of the > 96% identical DBL domain of *P. vivax* bound to DARC (PDB ID 4nuv). Whereas no significant substitutions were found in the DBL domain between both *P. simium* sequences, our analysis showed that residue substitutions between *P. simium* and *P. vivax* DBL domains cluster in proximity of the DARC binding site (Additional file 2: Figure S31). Based on our models, these substitutions are unlikely to negatively affect the association with DARC, supporting the idea that the DBL domains of both *P. simium* would be capable of binding to human DARC. Hence, the human-infecting *P. simium* DBL1 probably retains the capacity to bind to human DARC, but has the interacting domain positioned closer to the membrane than in the NHP-infecting CDC strain.

The deletion we detected in the *P. simium* RBP2a was larger, resulting in the loss of 1003 amino acid residues. These residues are predicted to form a mostly α-helical extracellular stem-like structure that positions the reticulocyte-binding domain away from the membrane (Fig. 3D). However, given that the deletion affects neither the transmembrane region, nor the receptor-binding domain, our analysis suggests the resulting truncated RBP2a protein could still associate with the human receptor, but that the binding event would occur closer to the merozoite membrane.

## Discussion

We present the genome of *Plasmodium simium*, the eighth malaria parasite species known to infect humans in nature. In recent evolutionary time, *P. simium* has undergone both anthroponosis and zoonosis making it unique for the study of the genetics underlying host switching in malaria parasites. Analysis of its genome confirmed a close phylogenetic relationship between *P. simium* and *P. vivax*, and further analyses on single-nucleotide divergence support a very recent American origin for *P. simium.*

Two proteins involved in host invasion, DBP1 and RBP2a, were found to harbour extensive deletions in *P. simium* compared to *P. vivax*. Given the involvement of RBP2a in *P. vivax* reticulocyte binding [39], the observed deletion in *P. simium* could signify an altered ability to infect reticulocytes. Interestingly, experimental analysis of *P. simium* samples revealed that isolates from human hosts all carried the DBP1 deletion, whereas isolates from NHPs displayed both absence and presence of the deletion. This DBP1 deletion is not present in the *P. simium* isolated from a brown howler monkey in the 1960s, which was previously shown to be incapable of infecting humans [23], although some degree of laboratory adaptation of this parasite may have affected its genome. However, this deletion is also absent in *P. vivax*, so cannot in itself explain the ability of *P. simium* to infect humans in the current outbreak. It is possible, however, that this deletion is required for *P. simium* to invade human red blood cells given the alterations that have occurred elsewhere in its red blood cell invadome following adaptation to non-human primates since the split between *P. simium* and its human-infecting *P. vivax* ancestor (Fig. 3B).

Our data is consistent with the hypothesis that the DBP1 deletion is required for efficient invasion of human RBCs by the *P. simium* parasites present in the Atlantic Forest. However, another study from Espírito Santo suggests that humans may be infected with *P. simium* strains that carry DBP1 without this deletion [40]. This discrepancy may be caused by genetic differences in the *P. simium* strains circulating in each region. Further, the distinction between *P. simium* and *P. vivax* infections based on epidemiological data may be confounded by human migration between the Amazonas and Espírito Santo [41].

Red blood cell invadome proteins are obvious candidates for genetic factors underlying host specificity; an inactivating mutation in a *P. falciparum* erythrocyte-binding antigen has recently been shown to underlie host specificity [42]. Traditionally, functional studies on red blood cell invadome proteins have focused on domains known to bind or interact directly with the host. Although the *P. simium*-specific DBP1 and RBP2a deletions reported here do not cover known structural motifs, these deletions could nevertheless affect host cell recognition as disordered protein regions have known roles in cellular regulation and signal transduction [43]. Further, a shorter, less flexible linker between the merozoite membrane and the receptor-binding DBP1 domain may favour a more rigid and better oriented positioning of the dimeric DBP1, enhancing its capacity to engage the human receptor.

Phylogenetic analyses of the *P. simium* clade give the geographical location of its most closely related *P. vivax* strain as Mexico, and not Brazil. In imported populations, the relationship between geographical and genetic proximity may be weak. Multiple introductions of diverse strains from founder populations may occur independently over large distances, so that two closely related strains may be introduced in distantly located regions. It may be postulated that there occurred the introduction of strains of *P. vivax* to Mexico from the Old World that were closely related (due to similar regions of origin) to strains introduced to the Atlantic Forest which then went on to become *P. simium* in New World monkeys. Strains from a different point of origin were introduced to the Amazonian region of Brazil. This hypothesis necessitates reproductive isolation of the *P. simium* clade from the Brazilian *P. vivax* parasites following their initial introduction; an isolation that would be facilitated, presumably, by their separate host ranges or via adaptation to different vectors.

Due to uncertainties regarding the number of individual genomes that were transferred during the original host switch from human to NHPs that resulted in the formation of the *P. simium* clade, it is impossible to perform dating analyses to determine a time for the split between *P. vivax* and *P. simium* with which we can be confident. The phylogeny shown in Fig. 1 is consistent with the hypothesis that all present-day *P. vivax*/*P. simium* originated from a now extinct, or as yet unsampled, Old World population. The most parsimonious explanation for this is that today’s New World *P. vivax/P. simium* originated from European *P. vivax*, which was itself a remnant of the original Eurasian/African *P. vivax* driven to extinction (or near extinction) in Africa by the evolution of the Duffy negative condition in the local human populations, and from Europe by malaria eradication programmes in the latter half of the twentieth century. This hypothesis is supported by the evidence of a close relationship between historical Spanish *P. vivax* and South American strains of the parasite [16], and by previous analyses of the mitochondrial genome [44]. Therefore, we postulate that the host switch between humans and non-human primates that eventually led to establishment of *P. simium* in howler monkeys must have occurred subsequent to the European colonisation of the Americas, within the last 600 years.

We find no evidence from the nuclear genome, the mitochondrial genome, or the apicoplast genome that any of the *P. vivax /P. simium* strains from the New World considered in our analyses are more closely related to Old World parasites than they are to each other, as previously contended [45].

Given the limited genetic diversity among the *P. simium* isolates considered here compared to that of *P. vivax*, it is almost certain that the original host switch occurred from humans to NHPs, and not the other way around [22]. Similarly, the larger amount of genetic diversity in the current NHP-infecting *P. simium* compared to those *P. simium* strains isolated from humans (as indicated by the higher degree of DBP1 polymorphism in the NHP-infecting *P. simium* compared to the strains infecting humans), suggests that humans are being infected from a pool of NHP parasites in a true zoonotic manner, as opposed to the sharing of a common parasite pool between humans and NHPs.

The biological definition of a species is a group of organisms that can exchange genetic material and produce viable offspring. We have no way of knowing whether this is the case for *P. vivax* and *P. simium*, and genetic crossing experiments would be required to resolve this question. Our phylogenetic analyses, however, clearly show *P. simium* forming a clade on its own within the broader diversity of *P. vivax*, and this strongly suggests, given what we know about its biology, that allopatric speciation has been/is occurring.

*Plasmodium simium* is currently recognised as a species separate from *P. vivax*; it has been well characterised and described in the literature, and there is a type specimen available, with which all the strains sequenced here cluster in one monophyletic group. Therefore, we cannot at present overturn the species status of *P. simium* in the absence of conclusive proof from crossing experiments.

## Conclusions

The recent outbreak of human malaria in the Atlantic Forest of Rio de Janeiro underlines the impact of zoonotic events on human health. Non-human primate malaria parasites must be considered a reservoir of potential infectious human parasites relevant to any malaria eradication strategy. Little is known about the genetic basis for zoonoses, yet the genome sequence of *P. simium* suggests a deletion within the DBP1 gene is a possible facilitator of zoonotic transfer. The genome of *P. simium* will thus form an important resource for the future functional characterizations of the mechanisms underlying zoonotic malaria.

## Methods

### Sample collection and preparation

Human and primate samples of *P. simium* were collected and prepared as part of a previous study [2, 14]. Additionally, two *P. vivax* samples from the Amazon area of Brazil were also collected from human patients (Additional file 1: Table S3). All participants provided informed written consent. The *P. simium* CDC (Howler) strain (Catalog No. MRA-353) from ATCC was obtained *via* the BEI Resources Repository in NIAID-NIH (https://www.beiresources.org/).

### DNA extraction and sequencing

DNA was extracted as previously described [2]. Genomic DNA for each sample was quantified using the Qubit® 2.0 Fluorometer and was used for library preparation. DNA for intact samples was sheared using a Covaris E220 DNA sonicator to fragments of 500 bp. The DNA libraries for intact samples were made using the TruSeq Nano DNA Library Prep kit (Illumina), whereas the DNA libraries for degraded samples were made using Ovation Ultralow Library System V2 kit (Nugen), according to the manufacturers’ instructions. The amplified libraries were stored at − 20 °C. The pooled libraries were sequenced in an Illumina HiSeq4000 instrument (2 × 150 bp PE reads) (Illumina). A PhiX control library was applied to the sequencing run as a base balanced sequence for the calibration of the instrument so that each base type is captured during the entire run. Samples AF22, AF26 and AF36 were additionally sequenced and scaffolded by PacBio RS II platform (Pacific Biosciences, CA, USA) using a SMRT library. Genomic DNA from the *P. vivax* samples was extracted from filter paper as previously described [46].

### Illumina reads preparation and mapping

FastQC v 0.11.6 (http://www.bioinformatics.babraham.ac.uk/projects/fastqc) was used to evaluate the quality of Illumina reads. Illumina adapters were removed, and reads were trimmed using the trimmomatic v0.33 [47] software with the following conditions:

*LEADING:20 TRAILING:20 SLIDINGWINDOW:4:20 MINLEN:36*

To exclude human reads from our analysis, trimmed reads were mapped against the human reference genome (v. hg38) and the *Plasmodium vivax* strain P01 reference genome (v. 36) from PlasmoDB (www.plasmodb.org) with bowtie2 (v 2.3.3.1) [48]. Reads mapping against the human genome were removed from further analysis.

### Genome assembly

*Plasmodium simium* sample AF22 was selected for genome assembly based on read quality and coverage. After removal of human contaminants, Illumina reads were assembled into contigs using the Spades (v 3.70) assembler [49]. Contigs assembled into scaffolds running SSPACE (v 3.0) [50] for 15 rounds and gaps filled with Gapfiller (v 1.10) [51]. Scaffolds were subsequently corrected with Illumina reads using the Pilon (v 1.22) software [52]. Blobtools (v 1.0) (DOI: 10.5281/zenodo.845347) [53] was used to remove any residual contaminant scaffolds. Genome size and GC content was in line with that of *P. vivax* (Additional file 1: Table S1). Genomic scaffolds representing the mitochondrial and apicoplast genome were identified through blastn searches against the corresponding *P. falciparum* and *P. vivax* sequences (Additional file 2: Figure S1). The *P. simium* mitochondrial genome was aligned against a range of previously published *P. vivax* and *P. simium* mitochondrial genomes [54, 55]. A gap-filled region in the alignment where the distal parts of the *P. simium* scaffold were merged was manually deleted. A minimum spanning haplotype network was produced using PopART [56, 57] confirming the authenticity of the *P. simium* mitochondrial genome (Additional file 2: Figure S32).

### Genome annotation

Two approaches were used to annotate the reference *P. simium* AF22 genome. Firstly, the Maker pipeline (v 2.31.8) [58] was run for two rounds, using ESTs and protein evidence from *P. vivax* and *P. cynomolgi* strain B and *P. falciparum* to generate Augustus gene models. Secondly, a separate annotation was produced using the Companion web server [59]. Companion was run using the *P. vivax* P01 reference assembly and default parameters. Basic annotation statistics are provided in Additional file 1: Table S1. The relatively low number of genes (5966) is due to the fragmented and incomplete nature of the *P. simium* assembly (Additional file 1: Table S1). Gene content was estimated using BUSCO [60, 61] (v3.0) revealing a gene annotation completeness comparable to other *Plasmodium* genome assemblies (Additional file 2: Figure S2).

### PlasmoDB genome references and annotations

Genome FASTA files, as well as annotated protein and CDS files were obtained from PlasmoDB [62] for the following species: *P. gallinaceum* 8A, *P. cynomolgi* B and M, *P knowlesi* H, *P. falciparum* 3D7, *P. reichenowi* G01, *P. malariae* UG01, *P. ovale curtisi* GH01, *P. coatneyi* Hackeri, *P. vivax* P01 and *P. vivax* SalI. For each species, PlasmoDB version 36 was used.

### Orthologous group determination

Amino acid sequence-based phylogenetic trees were prepared using protein sequences from the *P. simium* annotation, as well as the protein annotations from 10 malaria species downloaded from PlasmoDB: *P. vivax* P01, *P. cynomolgi* B, *P. knowlesi* H, *P. vivax*-like Pvl01, *P. coatneyi* Hackeri, *P. falciparum* 3D7, *P. gallinaceum* 8A, *P. malariae* UG01, *P. ovale* curtisi GH01 and *P. reichenowi* G01. *P. vivax*-like from PlasmoDB version 43, all other annotations from version 41. A total of 3181 1:1 orthologous genes were identified using the Proteinortho (v 6.0.3) software [63]. Approximately 88% of the predicted genes in *P. simium* have orthologues in *P. vivax* P01 (Additional file 2: Figure S33).

### Short indels in genes

Shorter indels (< 500 bp) were detected from soft-clipping information in read mapping using the ‘-i’ option in DELLY [37] (v 0.7.9). Predicted indels in protein-coding genes were then compared to independent assemblies of *P. simium* samples AF22, AF28, AF33, AF36, 2302 and 3636 (Additional file 1: Table S3) and indels present in both DELLY predictions and all assemblies were kept (Additional file 1: Table S7). Indel boundaries shifted by a maximum of two amino acid positions were allowed between predictions and assemblies. As assemblies were not complete, data for any gene was only required to be present in five of the six *P. simium* assemblies used.

### Protein phylogeny

Protein sequences were aligned using mafft (v 7.222) [64] and alignments were subsequently trimmed with trimAl (v 1.2rev59) [65] using the heuristic ‘automated1’ method to select the best trimming procedure. Trimmed alignments were concatenated, and a phylogenetic tree was constructed using RAxML (v 8.2.3) [66] with the PROTGAMMALG model.

### SNP calling and analysis

Short sequence reads from 15 *P. simium* samples (13 derived from humans and two from NHPs) and two *P. vivax* samples, all from this study (Additional file 1: Table S3), were aligned against a combined human (hg38) and *P. vivax* (strain P01, version 39) genome using NextGenMap (v0.5.5) [67]. This was similarly done for 30 previously published *P. vivax* strains [25] and the Sal1 reference. These data sets were downloaded from ENA (https://www.ebi.ac.uk/ena) (Additional file 1: Table S4). Duplicate reads were removed using samtools (v 1.9) [68], and the filtered reads were realigned using IndelRealigner from the GATK package (v 4.0.11) [69]. SNPs were called independently with GATK HaplotypeCaller and freebayes (v 1.2.0) [70], keeping only SNPs with a QUAL score above 30. The final SNP set was determined from the inter-section between GATK and freebayes. Allele frequencies and mean coverage across SNP sites are shown in Additional file 2: Figure S34. A PCA plot was constructed using plink (v 1.90) [71], and admixture analysis was done with Admixture (v 1.3.0) [26].

### SNP phylogeny

Alleles from SNP positions with data in 55 samples were retrieved, concatenated and aligned using mafft [64]. Tree was produced by PhyML [72, 73] with the GTR substitution model selected by SMS [74]. Branch support was evaluated with the Bayesian-like transformation of approximate likelihood ratio test, aBayes [75]. A phylogenetic network was made in SplitsTree [76] using the NeighborNet network [77].

### Nucleotide diversity

Conventional tools calculating nucleotide diversity directly from the variant call files assume that samples are aligned across the entire reference sequence. But as read coverage across the reference genome was highly uneven between samples (Additional file 2: Figure S34), adjustment for this was required. Coverage across the reference genome was thus calculated for each sample using samtools mpileup (v 1.9) [68]. For each comparison between two samples, the nucleotide divergence was calculated as number of detected bi-allelic SNPs per nucleotide with read coverage of at least 5× in both samples.

### Population divergence

To account for the differences in read coverage between samples we used the pixy software [78], which produces unbiased estimates of *D*_XY_ in the presence of missing data. Population divergence (*D*_XY_) was calculated from this all-site VCF using pixy version 1.0.4.beta1, either in 10-kb windows or in gene coordinate windows.

### Gene sequence deletions

Exploratory Neighbour-Joining phylogenies were produced with CLUSTALW [79, 80] and visualised with FigTree (https://github.com/rambaut/figtree/) after alignment with mafft [79]. Pacbio reads were aligned using Blasr (v 5.3.2) [81], short Illumina reads using NextGenMap (v0.5.5) [67]. Dotplots were produced with FlexiDot (v1.05) [82].

### Gene families and groups

Exported protein-coding gene sets were compiled from the literature [83–85]. Invasion genes were retrieved from Hu et al. (2010) [86]. Gene families were assessed in seven *Plasmodium* genomes (*P. simium*, *P. vivax* SalI, *P. vivax* P01, *P. vivax-like* Pvl01, *P. cynomolgi* M, *P. cynomolgi* B and *P. knowlesi* H) using the following pipeline: for all genomes, annotated genes were collected for each gene family. These ‘seed’ sequences were used to search all proteins from all genomes using BLASTP and best hits for all proteins were recorded. For each gene family ‘seed’ sequences were then aligned with mafft [64], trimmed with trimAl [65], and HMM models were then built using HMMer (http://hmmer.org/).

For PIR/VIR and PHIST genes, models were built for each genome independently, for all other gene families a single model was built from all genomes. These models were then used to search all proteins in all genomes. All proteins with best BLASTP hit to a ‘seed’ sequence from a given genome were sorted according to their bit score. The lowest 5% of hits were discarded and remaining proteins with best hits to a ‘seed’ sequence were assigned one ‘significant’ hit. As all proteins were searched against ‘seeds’ from the six annotated genomes (*P. simium* excluded), a maximum of six ‘significant’ BLAST hits could be obtained. Similarly, for each HMM model, the bottom 25% hits were discarded and remaining hits were considered ‘significant’. The final set of gene families consisted of previously annotated genes and un-annotated genes with at least two ‘significant’ hits (either BLASTP or HMM).

PIR protein sequences were clustered based on BLASTP similarity and visualised using the edge-weighted spring embedded layout in cytoscape [87]. A haplotype network of DBP1 sequences was constructed using PopART [56, 57].

### PCR amplification of DBP1 and RBP2a genes

PCR primers were initially designed from alignments between *P. vivax* and *P. simium* sequences and tested using Primer-BLAST [88] and PlasmoDB (www.plasmodb.org). For DBP1, the reaction was performed in 10 μL volumes containing 0.5 μM of each oligonucleotide primer, 1 μL DNA and 5 μL of Master Mix 2x (Promega) (0.3 units of Taq Polymerase, 200 μM each deoxyribonucleotide triphosphates and 1.5 mM MgCl2). Samples were run with the following settings: 2 min of activation at 95 °C, followed by 35 cycles with 30 s denaturation at 95 °C, 30 s annealing at 57 °C (Δ*T* = − 0.2 °C from 2nd cycle) and 1 min extension at 72 °C, then 5 min final extension at 72 °C.

For the RBP2a PCR, the reaction was performed in 10 μL volumes containing 0.5 μM of each oligonucleotide primer, 1 μL DNA, 0.1 μL PlatinumTaq DNA Polymerase High Fidelity (Invitrogen, 5 U/μL), 0.2 mM each deoxyribonucleotide triphosphates and 2 mM MgSO_4_. The PCR assays were performed with the following cycling parameters: an initial denaturation at 94 °C for 1.5 min followed by 40 cycles of denaturation at 94 °C for 15 s, annealing at 65 °C for 30 s (Δ*T* = − 0.2 °C from 2nd cycle) and extension at 68 °C for 3.5 min. All genotyping assays were performed in the thermocycler Veriti 96 wells, Applied Biosystems, and the amplified fragments were visualised by electrophoresis on agarose gels (2% for DBP1 and 1% for RBP2a) in 1× TAE buffer (40 mM Tris-acetate, 1 mM EDTA) with 5 μg/ mL ethidium bromide (Invitrogen) in a horizontal system (Bio-Rad) at 100 V for 30 min. Gels were examined with a UV transilluminator (UVP - Bio-Doc System).

To prevent cross-contamination, the DNA extraction and mix preparation were performed in ‘parasite DNA-free rooms’ distinct from each other. Furthermore, each of these separate areas has different sets of pipettes and all procedures were performed using filtered pipette tips. DNA extraction was performed twice on different days. Positive (DNA extracted from blood from patients with known *P. vivax* infection) and negative (no DNA and DNA extracted from individuals who have never traveled to malaria-endemic areas) controls were used in each round of amplification. DNA extracted from blood of a patient with high parasitemia for *P*. *vivax* and DNA of *P*. *simium* of a non-human primate with an acute infection and parasitemia confirmed by optical microscopy served as positive controls in the PCR assays. Primer sequences are provided in Additional file 2: Figure S24 and S29.

### Structural modelling of DBP1 and RBP2a genes

RaptorX [89] was used for prediction of secondary structure and protein disorder. Homology models for the DBL domain of *P. vivax* P01 strain, *P. simium* AF22, and the previously published CDC *P. simium* strain were produced by SWISS-MODEL [90], using the crystallographic structure of the DBL domain from *Plasmodium vivax* DBP bound to the ectodomain of the human DARC receptor (PDB ID 4nuv), with an identity of 98%, 96% and 96% for *P. vivax*, *P. simium* AF22 and *P. simium* CDC, respectively. QMEAN values were − 2.27, − 2.04 and − 2.03, respectively. The homology model for the reticulocyte-binding protein 2 (RBP2a) of *P. vivax* strain P01 was produced based on the cryoEM structure of the complex between the *P. vivax* RBP2b and the human transferrin receptor TfR1 (PDB ID 6d05) [33], with an identity of 31% and QMEAN value of − 2.46. The visualization and structural analysis of the produced models was done with PyMOL (https://pymol.org/2/).

## Supplementary Information

**Additional file 1:** Tables S1-S7. **Table S1**: Genome statistics. **Table S2**: *P. simium* gene family members. **Table S3**: *P. simium* and *P. vivax* samples from Brasil *et al*. [2] & de Alvarenga *et al*. [14]. **Table S4**: *P. vivax* samples from Hupalo *et al*. [25]. **Table S5**: DBP and RBP genes. **Table S6**: Overview of PCR genotyping. **Table S7**: Protein-coding genes with short indels.

**Additional file 2: Figure S1**. Apicoplast and mitochondrial genomes. Circos plots of the assembled apicoplast (top) and mitochondrial (bottom) genomes. The presence of protein-coding genes (grey), ribosomal-RNAs (green), and transfer-RNAs (red) is denoted by bars. For each gene category, outermost circle denotes genes on the forward strand, and the innermost circle denotes genes on the reverse strand. The two circular representations nearest to the center represent GC-skew and GC-content, respectively. **Figure S2**. Busco assembly assessment. Gene content in Plasmodium simium and other Plasmodium genome assemblies. BUSCO was run using the eukaryota odb9 data set containing 303 BUSCO groups. **Figure S3**. Protein phylogeny. Maximum likelihood phylogeny based on 3204 concatenated *Plasmodium* simium protein-coding genes with 1:1 orthologs across a selection of *Plasmodium* species (see Methods). Tree was constructed using RaxML with the GTRGAMMA model and *Plasmodium gallinaceum* as outgroup. Branch support from 100 bootstrap replicates. **Figure S4**. Gene families. A) The number of gene family members among *Plasmodium* genomes. Diameters of circles are proportional to the log10-transformed number of family members, as shown on the right. B) Bar plot showing the distribution of average read coverage across all annotated *P. simium* genes. The 5th and 95th percentiles (genes with the highest and lowest read coverage, respectively) are highlighted in dark. Above the plots the proportion of genes belonging to the PIR gene family is shown as pie charts for the two extreme percentiles and the remaining genes ('Rest'). Using a Fisher's exact two-sided test, the proportion of PIR genes in both the 95th and the 5th percentile are significantly higher than in the 'Rest' genes (p = 5.8e-80 and p = 5.1e-67, respectively). **Figure S5**. Pir/Vir gene clustering. Clustering of PIR protein sequences based on BLASTP similarity. Network threshold is a bit-score of 40 and visualized using the edge-weighted spring embedded layout in cytoscape 3. **Figure S6**. SNP counts. Top: Number of high-confidence SNPs detected in each sample. *Plasmodium simium* samples shown in dark red, and *Plasmodium vivax* samples in orange (see also Additional file 1: Tables S3 & S4). Bottom: For a given SNP loci data is not available from all samples. The bar chart shows how many SNP loci (left y-axis) that have data (coverage) in a given number of samples (x-axis). Only SNPs with data from at least 55 samples were used in this study, as indicated by the blue bars. Purple bars represent discarded SNPs. The cumulative fraction of SNPs is shown as red line (right x-axis). For example, slightly more than half of the SNP loci have data from at least 55 samples, whereas app. 75% of samples have data from at least 25 samples. **Figure S7**. PCA plot. A) Plot of the first two dimensions from a principal component analysis of 124,968 SNPs. *Plasmodium vivax* samples are denoted by their geographic origin. B) Magnification of American *vivax* samples. C) Cumulative contribution (in percent) of each eigenvector. The separate clustering of *Plasmodium simium* and American *P. vivax* samples was also observed when plotting 2nd versus 3rd dimensions, and 3rd versus 4th dimensions (not shown). **Figure S8**. MDS plot. Plot of Multidimensional Scaling of *Plasmodium simium* and *Plasmodium vivax* samples. **Figure S9**. SNP phylogeny. Phylogenetic tree constructed from SNP sites. Identical to Fig. 1, but with sample IDs shown at terminal branches. **Figure S10**. Phylogenetic network. Network from nucleotide diversity distances produced in SplitsTree using the NeighborNet network. A magnification of the central hubs (red rectangle) is shown at the bottom right. **Figure S11**. Admixture. Q-estimates from unsupervised ADMIXTURE clustering analysis at K from 2 to 10. *Plasmodium vivax* samples are ordered by geographic origin. The lowest cross-validation error was observed for K = 3. **Figure S12**. Gene DXY diversity. A) For different intervals of average DXY values (x-axis), the number of genes with thee values are plotted (y-axis, log_10_-scaled). B) Box plot showing the distributions of DXY values for members of selected gene families. The right-most, yellow box shows the DXY values for all genes that not member of a gene family. **Figure S13**. DBP phylogeny. Neighbor-Joining tree of *Plasmodium* DBP protein sequences. Sequences were aligned using mafft and tree produced with CLUSTALW. Support from 1000 bootstrap replicates. Tree visualized with FigTree, genetic distance shown below tree. *Plasmodium simium* and *Plasmodium vivax* sequences derived from this study are highlighted in red and blue, respectively. Remaining sequences are suffixed by their genome of origin (PvivP; *P. vivax* P01, PvivS; *P. vivax* SalI, PcynM; *P. cynomolgi* M, PcynB; *P. cynomolgi* B, PknoH; *P. knowlesi* H). **Figure S14**. RBP phylogeny. Neighbor-Joining tree of *Plasmodium* RBP protein sequences. Sequences were aligned using mafft and tree produced with CLUSTALW. Support from 1000 bootstrap replicates. Tree visualized with FigTree, genetic distance shown below tree. *Plasmodium simium* and *Plasmodium vivax* sequences derived from this study are highlighted in red and blue, respectively. Remaining sequences are suffixed by their genome of origin (PvivP; *P. vivax* P01, PvivS; *P. vivax* SalI, PcynM; *P. cynomolgi* M, PcynB; *P. cynomolgi* B, PknoH; *P. knowlesi* H). **Figure S15**. Coverage across RBP gene loci. Average read coverage across gene loci when mapping human *Plasmodium simium* (top) and *Plasmodium vivax* (bottom) reads onto the P. vivax P01 genome. The RBPs genes are shown above plots with the *P. vivax* P01 gene identifiers below plots. Genes absent in *P. simium* are highlighted in grey. Note that the *P. vivax* P01 genome contains two annotated RBP1a and RBP2d genes, respectively. The average read coverage per gene is shown as dots for each individual sample, and the combined distributions are outlined by boxes. A single *P. simium* sample, AF22, has a typical coverage of around 200X but is omitted from this representation for clarity. The coverage of the *P. simium* CDC strain is highlighted as red dots. **Figure S16**. coverage across flanking regions. The average read coverage across *Plasmodium vivax* DBP and RBP genes is compared to the average read coverage across flanking genomic regions. The log2 ratio between coverage at flanking region and coverage at gene was calculated for four regions: 10 kb-5 kb upstream of gene (UP2), 5 kb-0 kb upstream of gene (UP1), 0 kb-5 kb downstream of gene (DOWN1), 5 kb-10 kb downstream of gene (DOWN2). Boxplot denotes the range of ratios for *Plasmodium simium* samples (red boxes), American *Plasmodium vivax* samples (light blue), and remaining *Plasmodium vivax* samples (blue). Up- and downstream are defined based on genome coordinates irrespective of gene orientation. After Bonferroni correction, only the 'UP1' region at RBP3 for *P. simium* samples (p = 0.011, denoted by asterisk) had a probability below 0.05 of the mean being above zero assuming a normal distribution. **Figure S18**. Haplotype network of DBP1 sequences. Haplotype network (minimum spanning network) produced using PopART. Numbers of mutations are indicated by hatch marks on edges. *Plasmodium simium* samples are shown in red, *Plasmodium vivax* in green, and P. vivax-like in purple. The previously published *P. simium* CDC strain sequence (ACB42432) is shown in black and bold. **Figure S19**. Read support for DBP1 deletion patterns. Among *Plasmodium vivax* and *Plasmodium simium* samples multiple deletion patterns in the DBP1 gene are observed (top). Deletions found in *P. vivax* samples are arbitrarily denoted vivax 1-4. For each sample, the number of reads supporting a given deletion is shown (bottom). Samples with reads supporting multiple deletion forms are indicated by asterisks. **Figure S20**. Dotplot. Similarity DNA dot plots of DBP1 genes. Top plots show *Plasmodium simium* and bottom plots show *Plasmodium vivax* DBP1 genes. Introns are denoted by grey rectangles and the deleted region (or site of deleted region in *P. simium*) is highlighted in red. **Figure S21**. Read coverage across the DBP1 deletion in *Plasmodium simium* samples. Artemis representation of read coverage across the *Plasmodium simium* DBP1 deletion. Reads from *Plasmodium vivax* samples AM01 & AM02 (two top panels) and *P. simium* AF22 & AF36 samples (two bottom panels) were mapped onto the *P. simium* AF22 assembly. The site of deletion is indicated by vertical red arrows. Note the lack of *P. vivax* reads spanning the deletion site. **Figure S22**. read coverage across the DBP1 deletion in *Plasmodium vivax* samples. Artemis representation of read coverage across the *Plasmodium simium* DBP1 deletion. Reads from *P. simium* AF22 & AF36 samples were mapped onto the *Plasmodium vivax* P01 genome. The site of deletion is indicated by red squares. Note the lack of *P. simium* coverage across the deleted region. **Figure S23**. PacBio read coverage across the DBP1 deletion in *Plasmodium simium*. Schematic depiction of PacBio reads mapping across the deletion in DBP1 gene. X-axis denotes genomic positions. Black line at the centre of plot shows the extent of exons. Reads mapping on the forward strand are shown above exons, reverse strand reads underneath exons. Reads are colored according to the sample from which they are derived. The site of deletion is indicated by arrows and thin vertical orange lines. **Figure S24**. DBP1 PCR. Top: Schematic overview of DBP1 deletion PCR approach. Gel images shown for human *Plasmodium vivax* samples (top gel image), human *Plasmodium simium* samples (middle image), and non-human primate (NHP) *P. simium* samples (bottom image). Expected band sizes with and without deletion event are indicated by red triangles. Bottom: Primer sequences. **Figure S26**. Read coverage across the RBP2a deletion in *Plasmodium simium* samples. Artemis representation of read coverage across the *Plasmodium simium* RBP2a deletion. Reads from *vivax* samples AM01 & AM02 (two top panels) and *P. simium* AF22 & AF36 samples (two bottom panels) were mapped onto the *P. simium* AF22 assembly. The site of deletion is indicated by vertical red arrows. Note the lack of *Plasmodium vivax* reads spanning the deletion site. **Figure S27**. Read coverage across the RBP2a deletion in *Plasmodium vivax* samples. Artemis representation of read coverage across the *Plasmodium simium* RBP2a deletion. Reads from *P. simium* AF22 & AF36 samples were mapped onto the *P. vivax* P01 genome. The site of deletion is indicated by red squares. Note the lack of *P. simium* coverage across the deleted region. **Figure S28**. PacBio read coverage across RBP2a deletion in *Plasmodium simium*. Schematic depiction of PacBio reads mapping across deletions in the RBP2a gene. X-axis denotes genomic positions. Black line at the centre of plots shows the extent of exons. Reads mapping on the forward strand are shown above exons, reverse strand reads underneath exons. Reads are colored according to the sample from which they are derived. Sites of deletions are indicated by arrows and thin vertical orange lines. **Figure S29**. RBP2a PCR. Top: Schematic overview of RBP2a deletion PCR approach. Gel images shown for *Plasmodium vivax* and *Plasmodium simium* samples. Expected band sizes with and without deletion event are indicated by red triangles. Bottom: Primer sequences. **Figure S30**. Short indels. A) Pie chart showing the percentage of indels being integers of 1-3 base pairs. B) The percentage of insertions (left bar) and deletions (middle) overlapping low-complexity regions in proteins. The percentage of all proteins consisting of low-complexity sequences is shown on the right. Low-complexity annotation downloaded from PlasmoDB. C) Size distributions of genes with and without indels. Genes with indels are listed in Additional file 1: Table S7. **Figure S31**. DBP1 protein structures. *Plasmodium simium* DBP1 is predicted to associate with human DARC. Left: Top-view of modelled DBP1 from *Plasmodium vivax* strain P01, human-infecting *P. simium* AF22 (PsDBP1) and monkey-infecting *P. simium* (CDC PsDBP1) sequences. Models were established based on the crystal structure of the *P. vivax* DBP1, strain Salvador 1, in complex with human DARC (PDB 4nuv). Individual chains of the DBP1 dimer are shown in light and dark blue. DARC (residues 19-30) is coloured in grey. Residue substitutions between the models and the crystal structure are highlighted in magenta. Right: close-up view of the DARC-binding site, as delimited by a dashed square on the left. Colours as in the left panel. The rearrangement of hydrogen-bonds (black dotted lines) is shown for the Lys-Asn substitution. **Figure S32**. Mitochondrial haplotype network. Haplotype network (minimum spanning network) produced using PopART (see Methods). Numbers of mutations are shown on edges. The two identical *Plasmodium simium* samples (AF22 and GenBank accession AY722798) are shown in black. *Plasmodium vivax* haplogroups coloured according to country of origin. Circle sizes indicate the number of sequences in haplogroups. **Figure S33**. Protein orthology. Venn diagram showing the number of shared gene orthogroups between *Plasmodium simium* and three other *Plasmodium* genomes. **Figure S34**. SNP allele frequencies & coverage. Top: Allele frequencies for SNPs with available data from at least 37 *Plasmodium simium* or *Plasmodium vivax* samples. Bottom: Mean read coverage at SNP sites shown for each samples.

**Additional file 3: Figure S17**. DBP1 alignment. Complete alignment of DBP1 protein sequences. The presence of the DBL domain (Pfam: PF0311) and the trans-membrane domain is indicated.

**Additional file 4: Figure S25**. RBP2a alignment. Complete alignment of RBP2a protein sequences.

## Data Availability

The reference genome assembly and short sequence reads have been uploaded to European Nucleotide Archive (https://www.ebi.ac.uk/ena/) under the Study accession number PRJEB34061.
