# Supplementary material for: The genome of the zoonotic malaria parasite Plasmodium simium reveals adaptations to host switching
Source: BMC Biol. 2021 Oct 1;19:219. doi: 10.1186/s12915-021-01139-5 (PMC8485552; doi:10.1186/s12915-021-01139-5)
Supplement: Supplementary file 2 — Additional file 2: Figure S1. Apicoplast and mitochondrial genomes. Circos plots of the assembled apicoplast (top) and mitochondrial (bottom) genomes. The presence of protein-coding genes (grey), ribosomal-RNAs (green), and transfer-RNAs (red) is denoted by bars. For each gene category, outermost circle denotes genes on the forward strand, and the innermost circle denotes genes on the reverse strand. The two circular representations nearest to the center represent GC-skew and GC-content, respectively. Figure S2. Busco assembly assessment. Gene content in Plasmodium simium and other Plasmodium genome assemblies. BUSCO was run using the eukaryota odb9 data set containing 303 BUSCO groups. Figure S3. Protein phylogeny. Maximum likelihood phylogeny based on 3204 concatenated Plasmodium simium protein-coding genes with 1:1 orthologs across a selection of Plasmodium species (see Methods). Tree was constructed using RaxML with the GTRGAMMA model and Plasmodium gallinaceum as outgroup. Branch support from 100 bootstrap replicates. Figure S4. Gene families. A) The number of gene family members among Plasmodium genomes. Diameters of circles are proportional to the log10-transformed number of family members, as shown on the right. B) Bar plot showing the distribution of average read coverage across all annotated P. simium genes. The 5th and 95th percentiles (genes with the highest and lowest read coverage, respectively) are highlighted in dark. Above the plots the proportion of genes belonging to the PIR gene family is shown as pie charts for the two extreme percentiles and the remaining genes ('Rest'). Using a Fisher's exact two-sided test, the proportion of PIR genes in both the 95th and the 5th percentile are significantly higher than in the 'Rest' genes (p = 5.8e-80 and p = 5.1e-67, respectively). Figure S5. Pir/Vir gene clustering. Clustering of PIR protein sequences based on BLASTP similarity. Network threshold is a bit-score of 40 and visualized using the edge-weighted [file 12915_2021_1139_MOESM2_ESM.pdf]

Figure S1

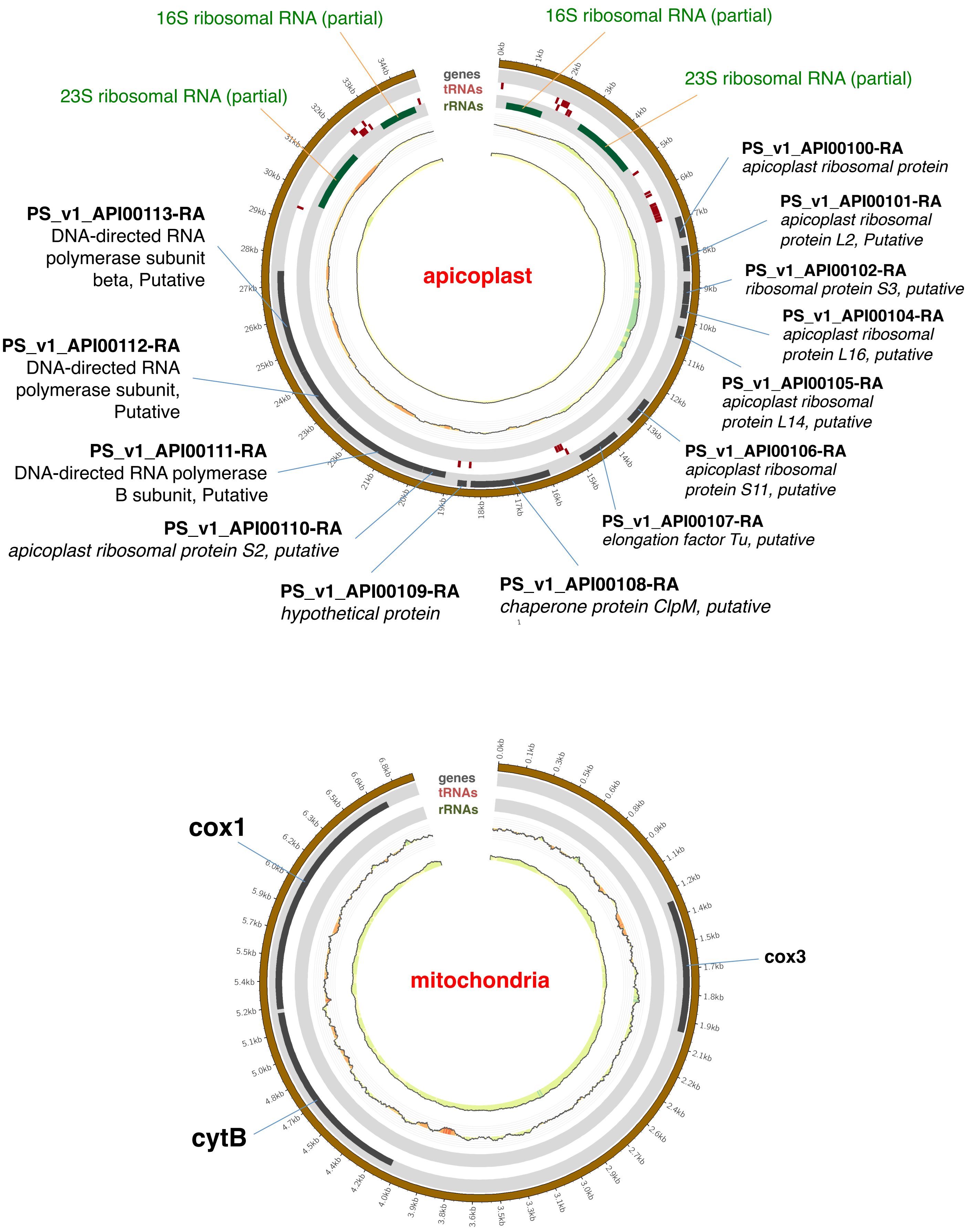
16S ribosomal RNA (partial)
16S ribosomal RNA (partial)
23S ribosomal RNA (partial)
23S ribosomal RNA (partial)
genes
tRNAs
rRNAs
apicoplast
PS_v1_API00100-RA
apicoplast ribosomal protein
PS_v1_API00101-RA
apicoplast ribosomal protein L2, Putative
PS_v1_API00102-RA
ribosomal protein S3, putative
PS_v1_API00104-RA
apicoplast ribosomal protein L16, putative
PS_v1_API00105-RA
apicoplast ribosomal protein L14, putative
PS_v1_API00106-RA
apicoplast ribosomal protein S11, putative
PS_v1_API00107-RA
elongation factor Tu, putative
PS_v1_API00108-RA
chaperone protein ClpM, putative
PS_v1_API00109-RA
hypothetical protein
PS_v1_API00110-RA
apicoplast ribosomal protein S2, putative
PS_v1_API00111-RA
DNA-directed RNA polymerase B subunit, Putative
PS_v1_API00112-RA
DNA-directed RNA polymerase subunit, Putative
PS_v1_API00113-RA
DNA-directed RNA polymerase subunit beta, Putative
genes
tRNAs
rRNAs
mitochondria
cox1
cox3
cytB

Figure S2

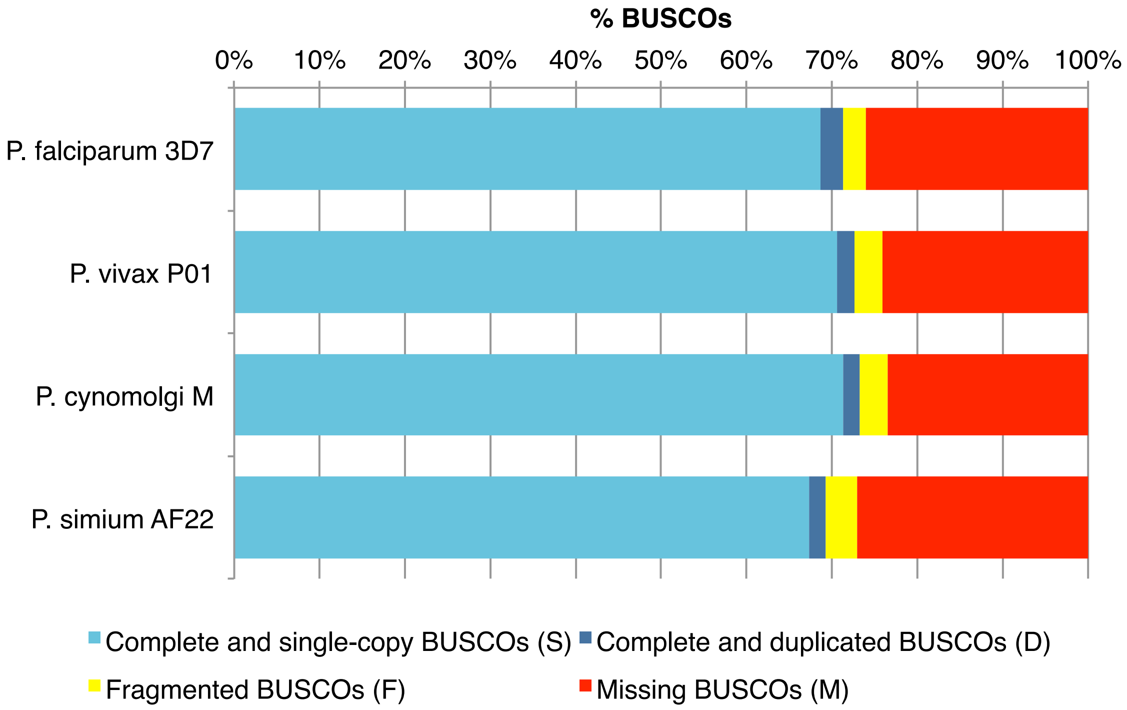
% BUSCOs
0%
10%
20%
30%
40%
50%
60%
70%
80%
90%
100%
P. falciparum 3D7
P. vivax P01
P. cynomolgi M
P. simium AF22
Complete and single-copy BUSCOs (S)
Complete and duplicated BUSCOs (D)
Fragmented BUSCOs (F)
Missing BUSCOs (M)

Figure S3

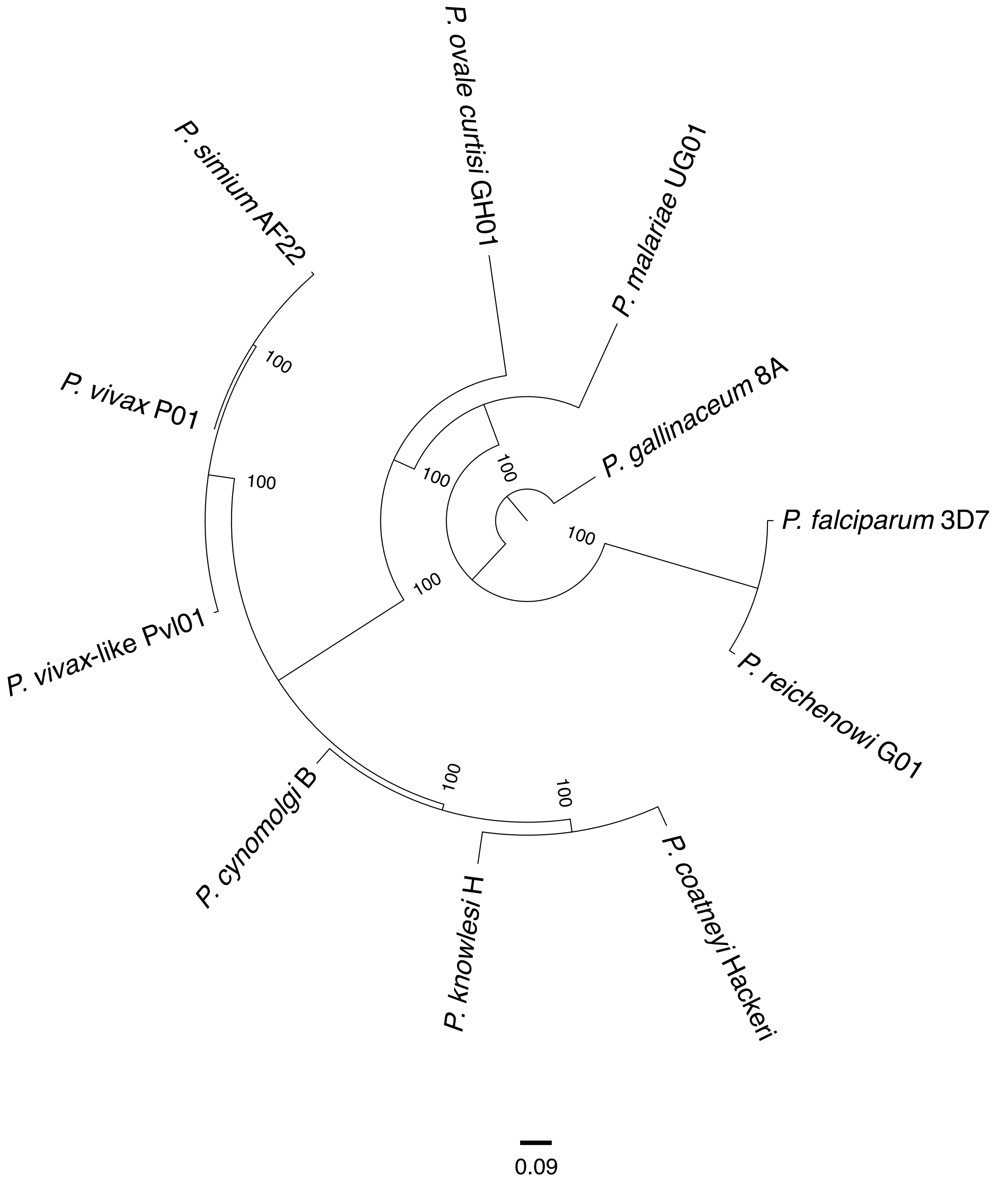
P. ovale curtisi GH01
P. malariae UG01
P. simium AF22
P. vivax P01
P. gallinaceum 8A
P. falciparum 3D7
P. vivax-like PvI01
P. reichenowi G01
P. cynomolgi B
P. knowlesi H
P. coatneyi Hackeri
100
100
100
100
100
100
100
100
0.09

Figure S4

A

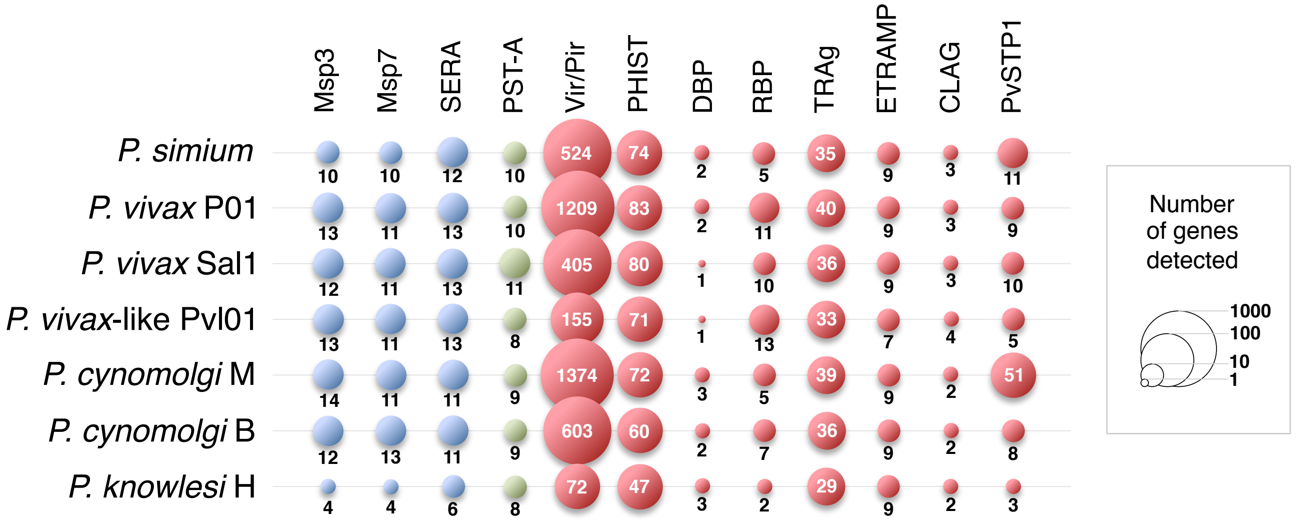

Msp3
Msp7
SERA
PST-A
Vir/Pir
PHIST
DBP
RBP
TRAg
ETRAMP
CLAG
PvSTP1
P. simium
P. vivax P01
P. vivax Sal1
P. vivax-like PvI01
P. cynomolgi M
P. cynomolgi B
P. knowlesi H
Number of genes detected
1000
100
10
1

B

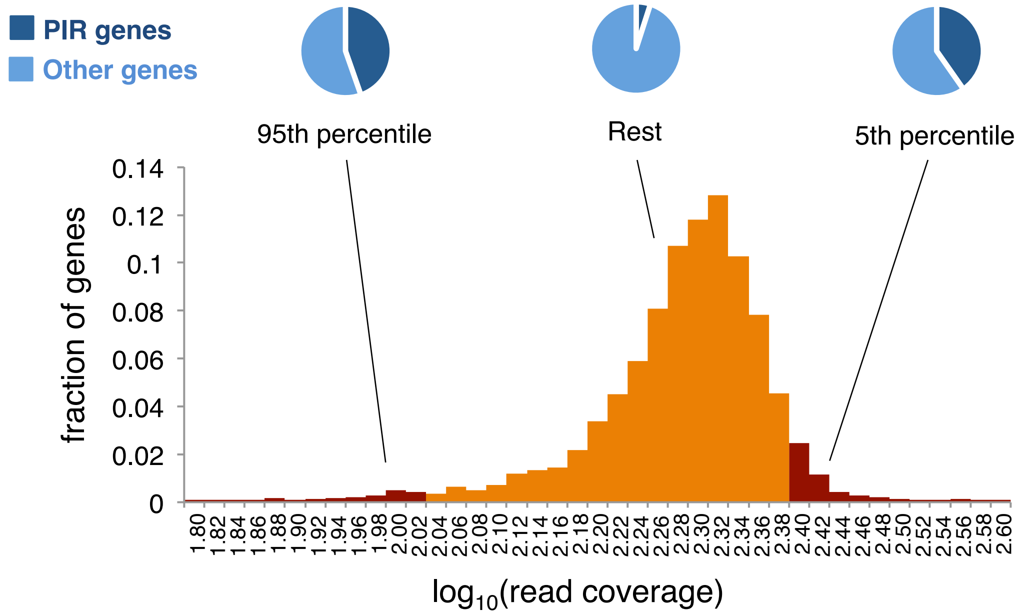

PIR genes
Other genes
95th percentile
Rest
5th percentile
fraction of genes
log10(read coverage)

Figure S5

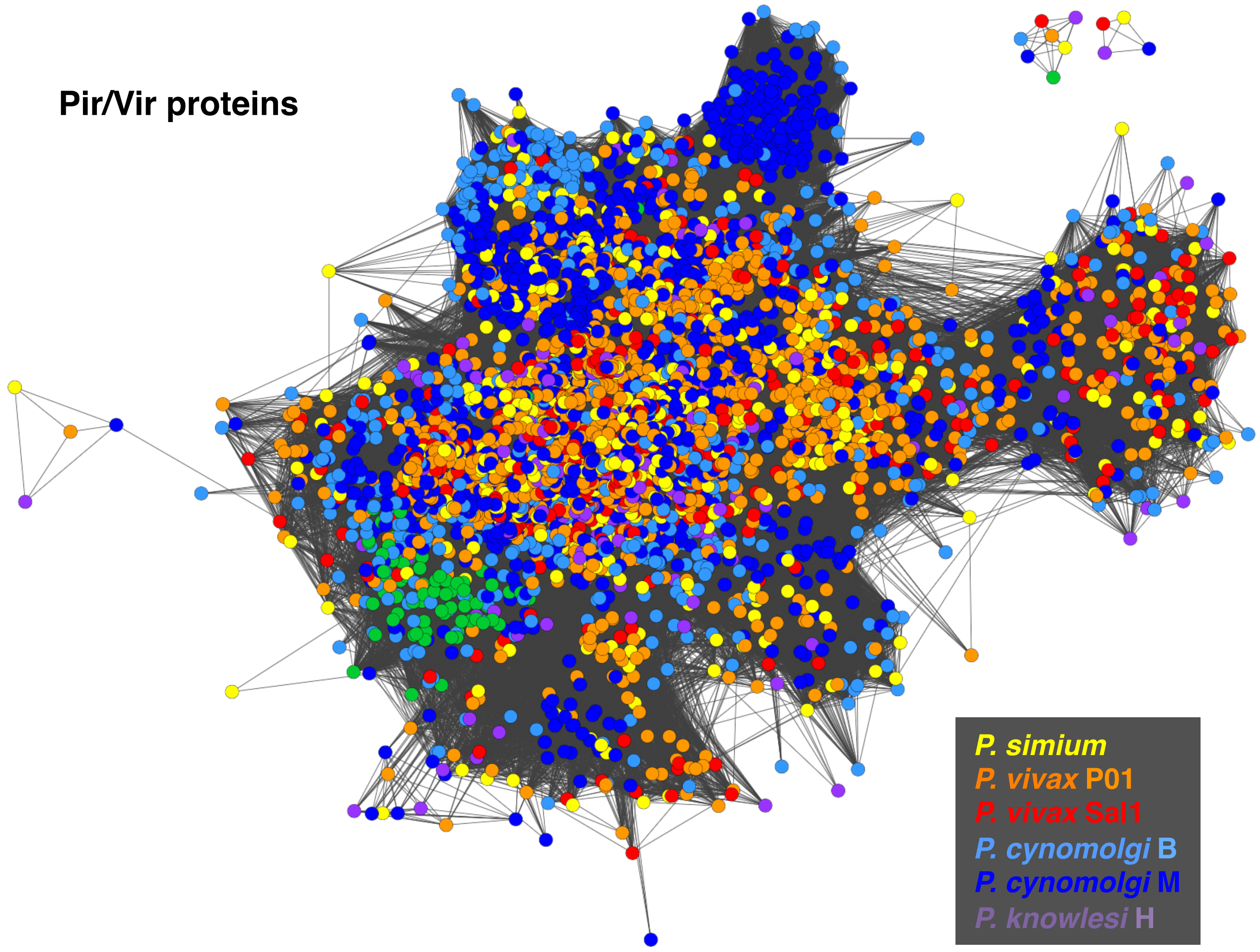

Pir/Vir proteins
P. simium
P. vivax P01
P. vivax Sal1
P. cynomolgi B
P. cynomolgi M
P. knowlesi H

Figure S6

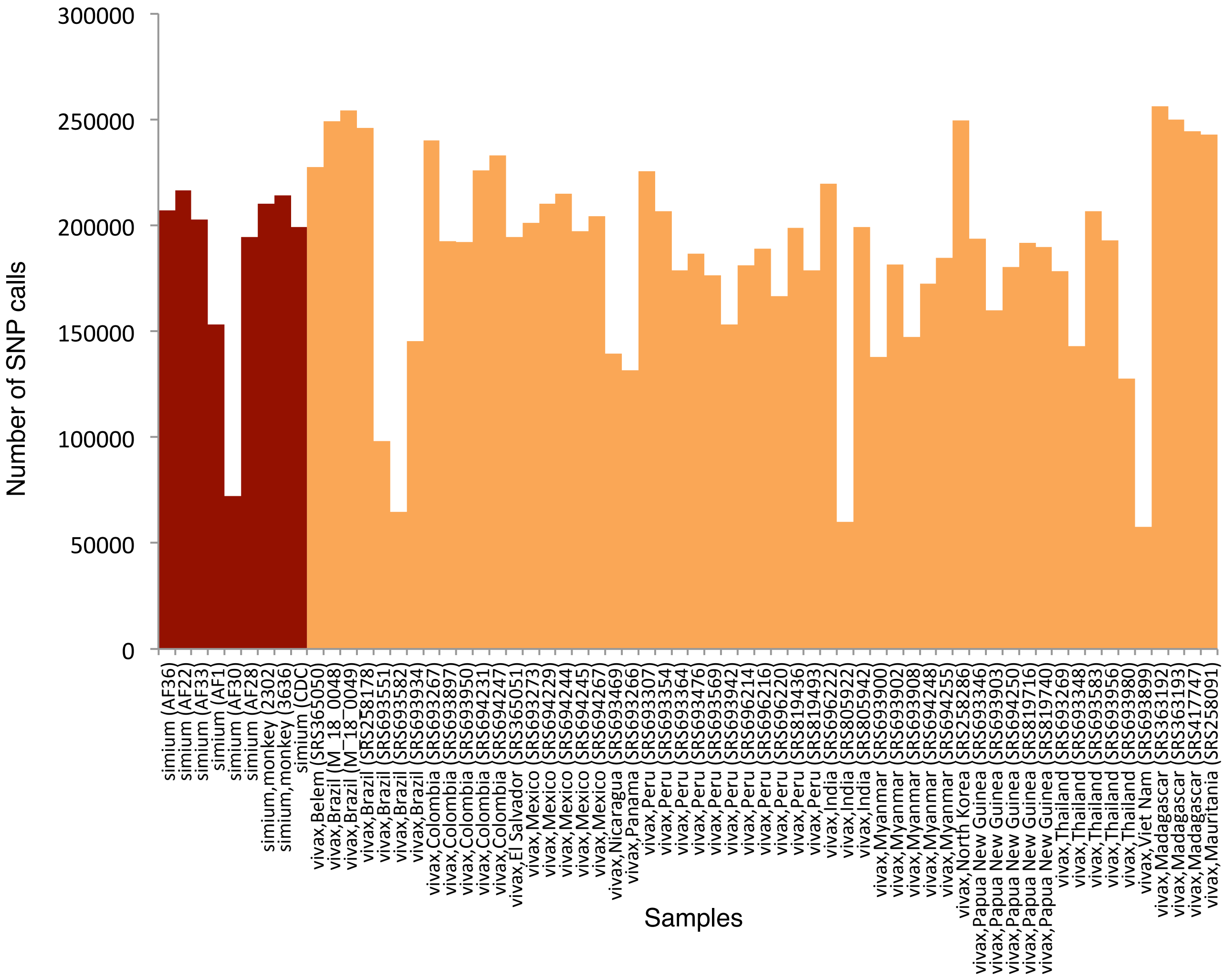
Number of SNP calls
300000
250000
200000
150000
100000
50000
0
simium (AF36)
simium (AF22)
simium (AF33)
simium (AF1)
simium (AF30)
simium (AF28)
simium,monkey (2302)
simium,monkey (3636)
simium (CDC)
vivax,Belem (SRS365050)
vivax,Brazil (M_18_0048)
vivax,Brazil (M_18_0049)
vivax,Brazil (SRS258178)
vivax,Brazil (SRS693551)
vivax,Brazil (SRS693582)
vivax,Brazil (SRS693934)
vivax,Colombia (SRS693267)
vivax,Colombia (SRS693897)
vivax,Colombia (SRS693950)
vivax,Colombia (SRS694231)
vivax,Colombia (SRS694247)
vivax,El Salvador (SRS365051)
vivax,Mexico (SRS693273)
vivax,Mexico (SRS694229)
vivax,Mexico (SRS694244)
vivax,Mexico (SRS694245)
vivax,Mexico (SRS694267)
vivax,Nicaragua (SRS693469)
vivax,Panama (SRS693266)
vivax,Peru (SRS693307)
vivax,Peru (SRS693354)
vivax,Peru (SRS693364)
vivax,Peru (SRS693476)
vivax,Peru (SRS693569)
vivax,Peru (SRS693942)
vivax,Peru (SRS696214)
vivax,Peru (SRS696216)
vivax,Peru (SRS696220)
vivax,Peru (SRS819436)
vivax,Peru (SRS819493)
vivax,India (SRS696222)
vivax,India (SRS805922)
vivax,India (SRS805942)
vivax,Myanmar (SRS693900)
vivax,Myanmar (SRS693902)
vivax,Myanmar (SRS693908)
vivax,Myanmar (SRS694248)
vivax,Myanmar (SRS694255)
vivax,North Korea (SRS258286)
vivax,Papua New Guinea (SRS693346)
vivax,Papua New Guinea (SRS693903)
vivax,Papua New Guinea (SRS694250)
vivax,Papua New Guinea (SRS819716)
vivax,Papua New Guinea (SRS819740)
vivax,Thailand (SRS693269)
vivax,Thailand (SRS693348)
vivax,Thailand (SRS693583)
vivax,Thailand (SRS693956)
vivax,Thailand (SRS693980)
vivax,Viet Nam (SRS693899)
vivax,Madagascar (SRS363192)
vivax,Madagascar (SRS363193)
vivax,Madagascar (SRS417747)
vivax,Mauritania (SRS258091)
Samples

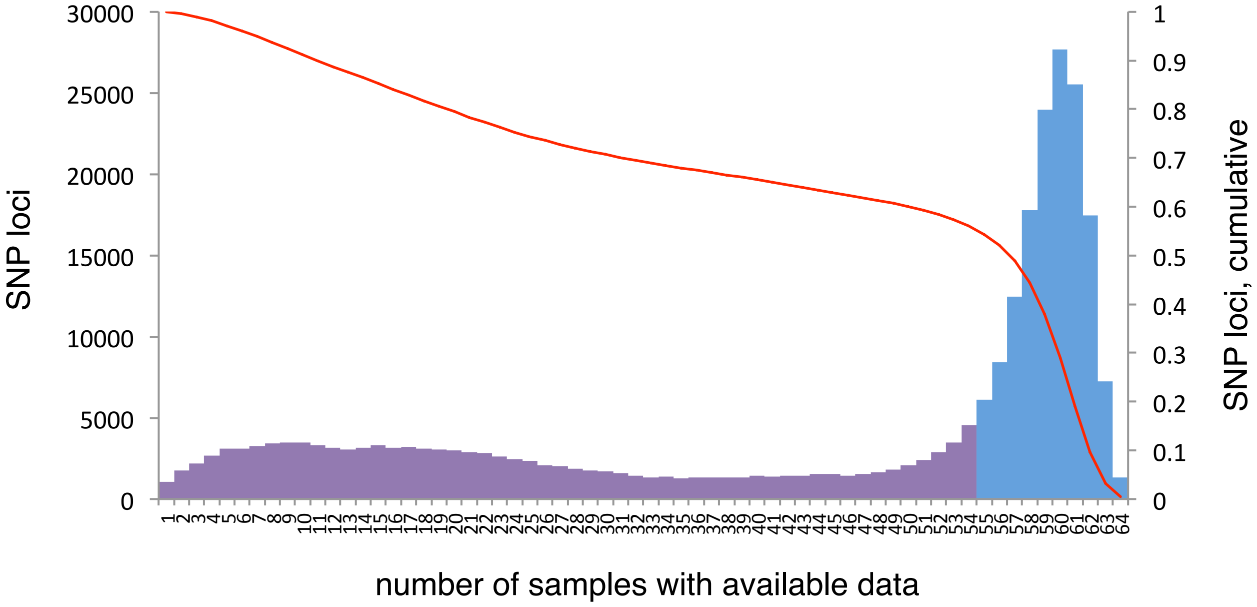
SNP loci
30000
25000
20000
15000
10000
5000
0
1 2 3 4 5 6 7 8 9 10 11 12 13 14 15 16 17 18 19 20 21 22 23 24 25 26 27 28 29 30 31 32 33 34 35 36 37 38 39 40 41 42 43 44 45 46 47 48 49 50 51 52 53 54 55 56 57 58 59 60 61 62 63 64
SNP loci, cumulative
1
0.9
0.8
0.7
0.6
0.5
0.4
0.3
0.2
0.1
0
number of samples with available data

Figure S7

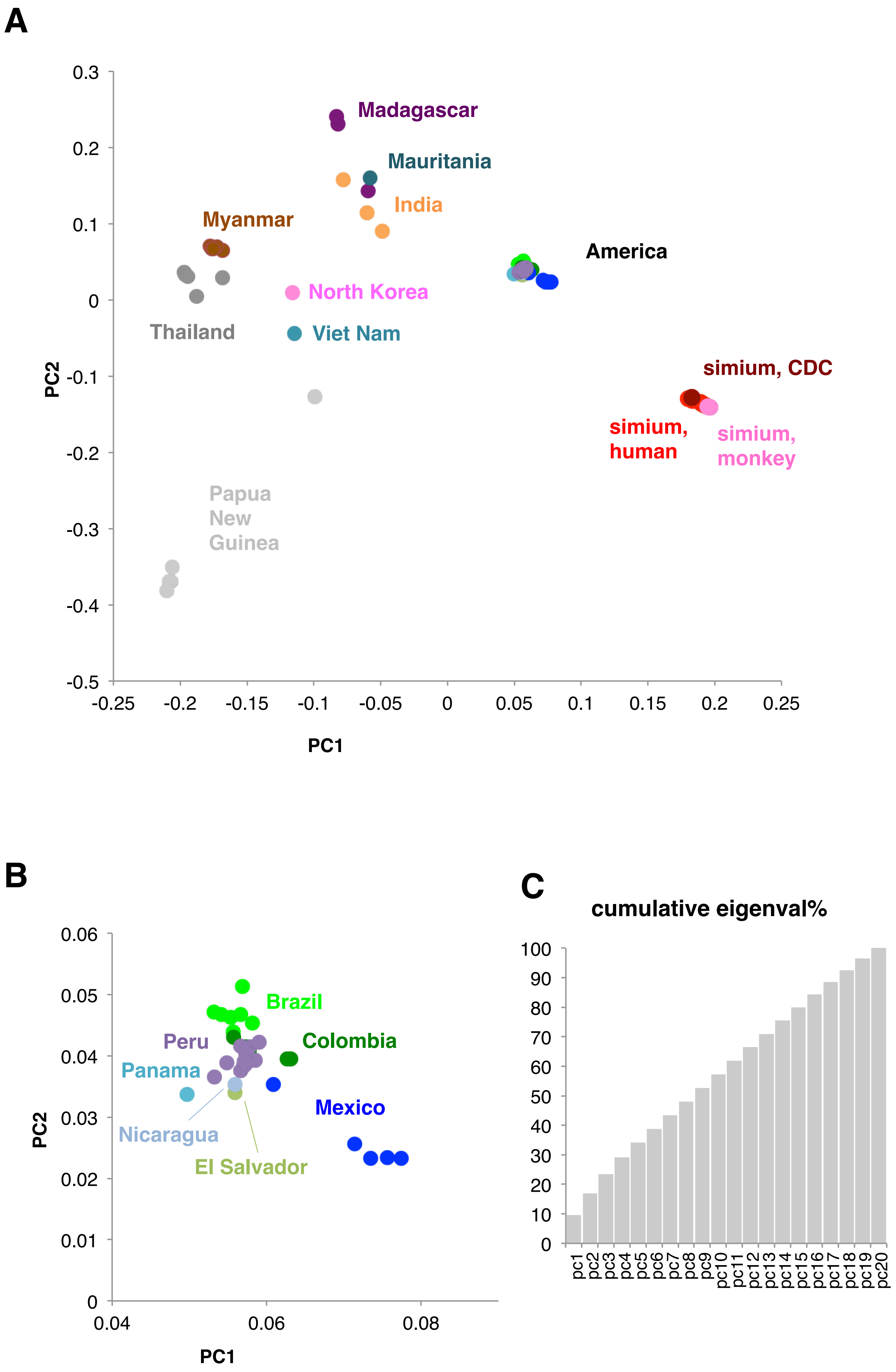
A
Madagascar
Mauritania
India
Myanmar
America
North Korea
Thailand
Viet Nam
simium, CDC
simium, human
simium, monkey
Papua New Guinea
PC2
PC1
0.3
0.2
0.1
0
-0.1
-0.2
-0.3
-0.4
-0.5
-0.25
-0.2
-0.15
-0.1
-0.05
0
0.05
0.1
0.15
0.2
0.25
B
Brazil
Peru
Colombia
Panama
Mexico
Nicaragua
El Salvador
PC2
PC1
0.06
0.05
0.04
0.03
0.02
0.01
0
0.04
0.06
0.08
C
cumulative eigenval%
100
90
80
70
60
50
40
30
20
10
0
pc1
pc2
pc3
pc4
pc5
pc6
pc7
pc8
pc9
pc10
pc11
pc12
pc13
pc14
pc15
pc16
pc17
pc18
pc19
pc20

Figure S8

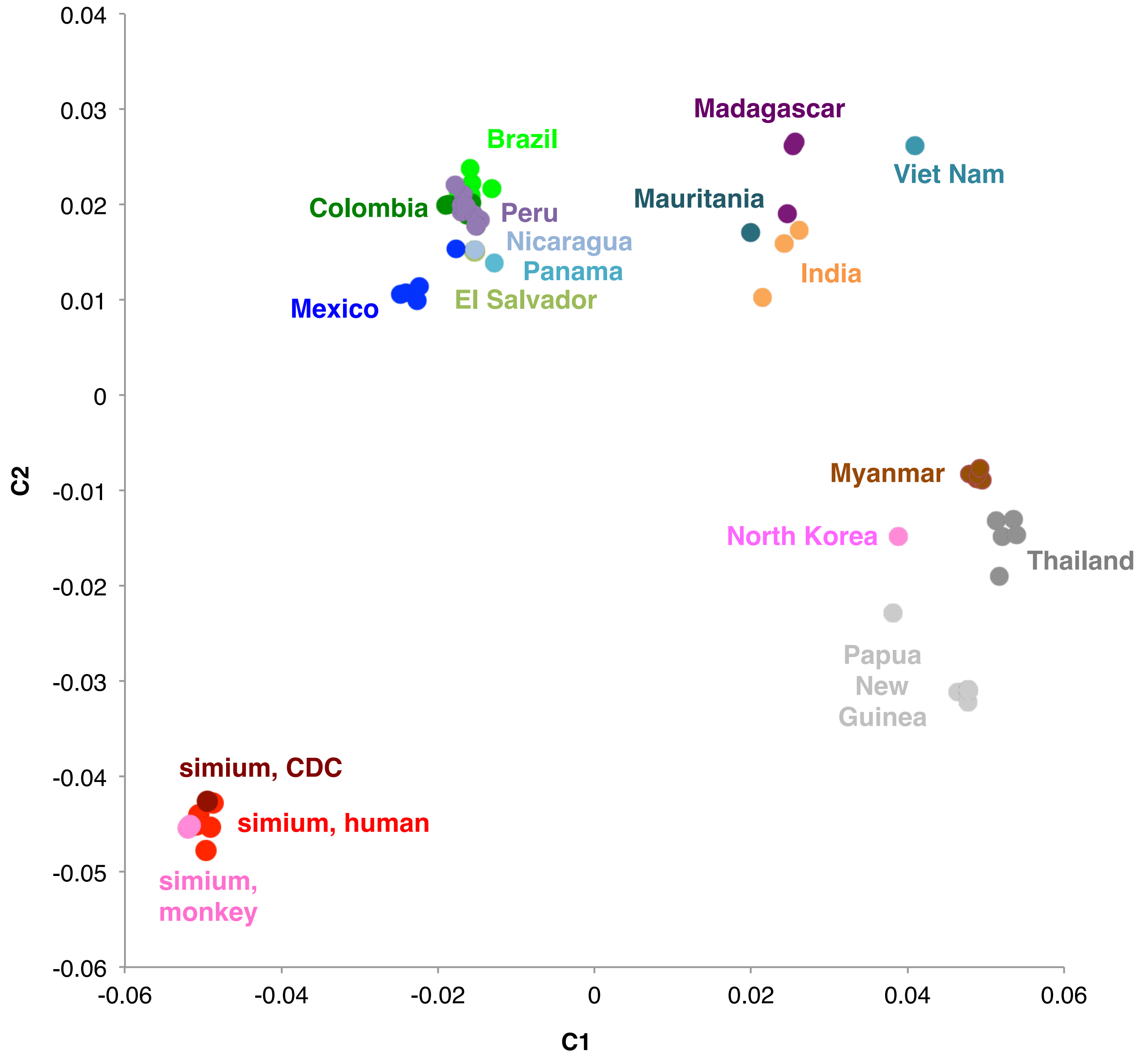
0.04
0.03
0.02
0.01
0
-0.01
-0.02
-0.03
-0.04
-0.05
-0.06
C2
-0.06
-0.04
-0.02
0
0.02
0.04
0.06
C1
Brazil
Colombia
Peru
Nicaragua
Panama
El Salvador
Mexico
Madagascar
Viet Nam
Mauritania
India
Myanmar
North Korea
Thailand
Papua
New
Guinea
simium, CDC
simium, human
simium,
monkey

Figure S9

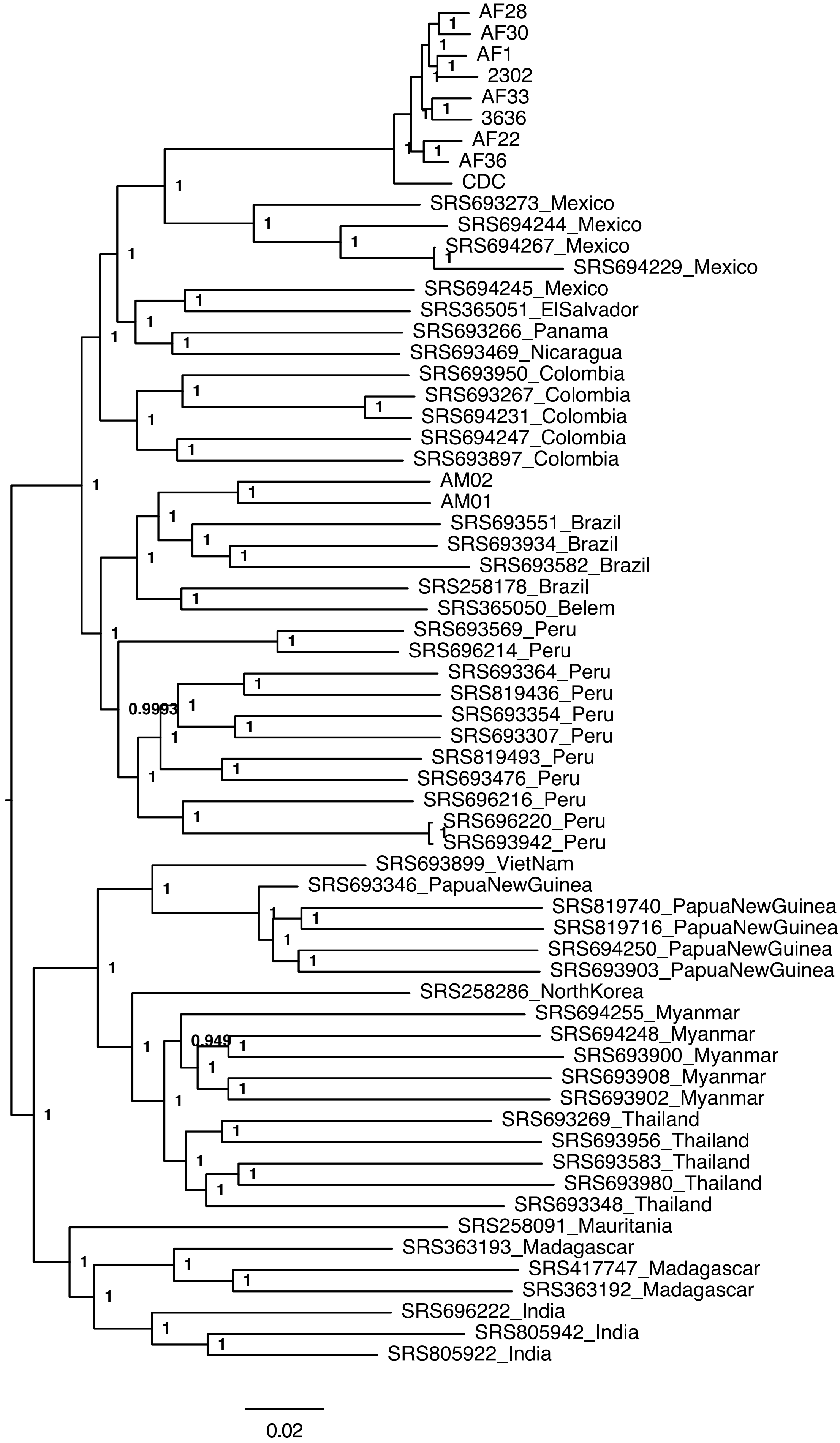
AF28
AF30
AF1
2302
AF33
3636
AF22
AF36
CDC
SRS693273_Mexico
SRS694244_Mexico
SRS694267_Mexico
SRS694229_Mexico
SRS694245_Mexico
SRS365051_ElSalvador
SRS693266_Panama
SRS693469_Nicaragua
SRS693950_Colombia
SRS693267_Colombia
SRS694231_Colombia
SRS694247_Colombia
SRS693897_Colombia
AM02
AM01
SRS693551_Brazil
SRS693934_Brazil
SRS693582_Brazil
SRS258178_Brazil
SRS365050_Belem
SRS693569_Peru
SRS696214_Peru
SRS693364_Peru
SRS819436_Peru
SRS693354_Peru
SRS693307_Peru
SRS819493_Peru
SRS693476_Peru
SRS696216_Peru
SRS696220_Peru
SRS693942_Peru
SRS693899_VietNam
SRS693346_PapuaNewGuinea
SRS819740_PapuaNewGuinea
SRS819716_PapuaNewGuinea
SRS694250_PapuaNewGuinea
SRS693903_PapuaNewGuinea
SRS258286_NorthKorea
SRS694255_Myanmar
SRS694248_Myanmar
SRS693900_Myanmar
SRS693908_Myanmar
SRS693902_Myanmar
SRS693269_Thailand
SRS693956_Thailand
SRS693583_Thailand
SRS693980_Thailand
SRS693348_Thailand
SRS258091_Mauritania
SRS363193_Madagascar
SRS417747_Madagascar
SRS363192_Madagascar
SRS696222_India
SRS805942_India
SRS805922_India
0.9993
0.949
1
0.02

Figure S10

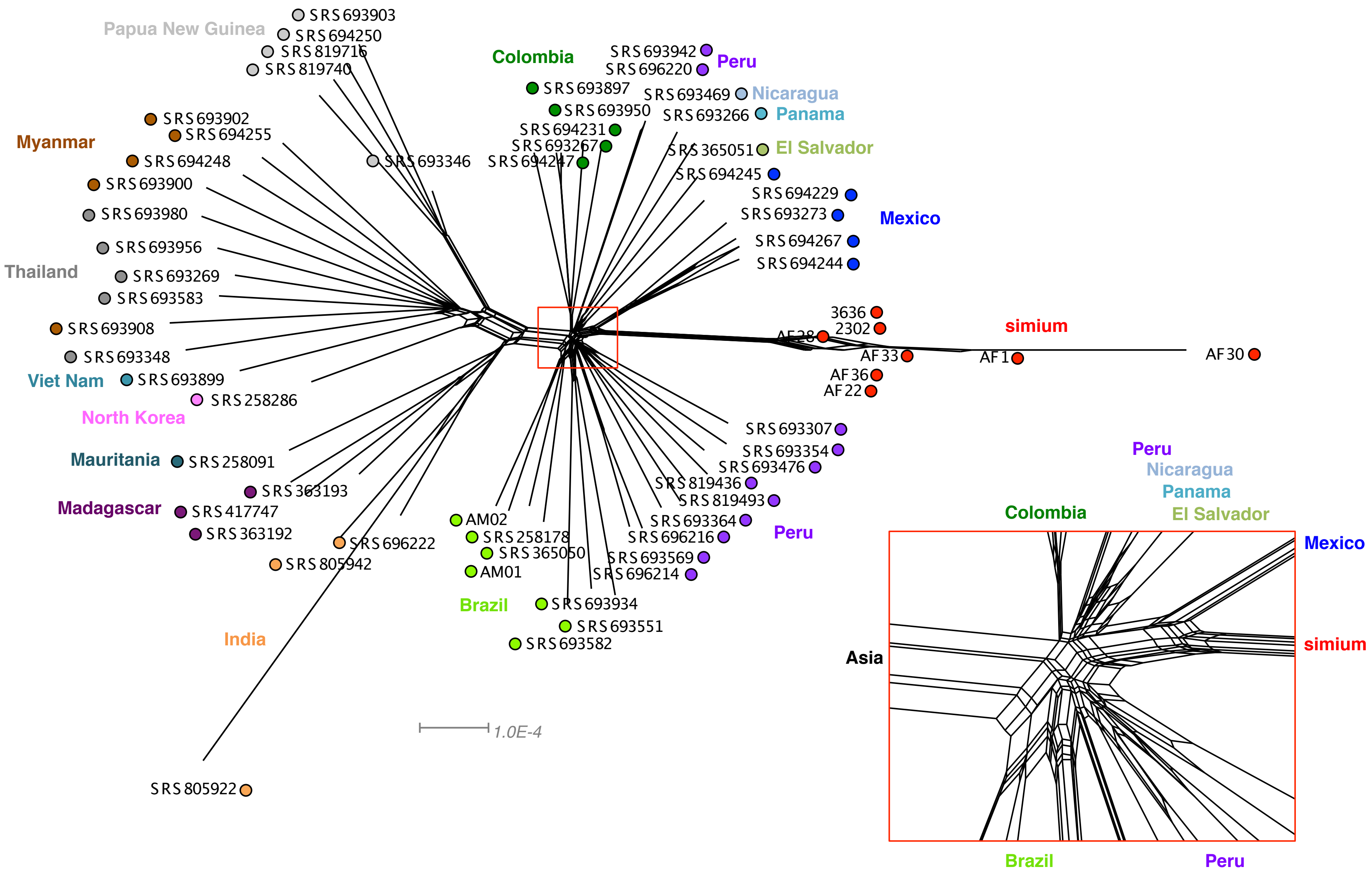

Papua New Guinea
SRS693903
SRS694250
SRS819716
SRS819740
SRS693346
Myanmar
SRS693902
SRS694255
SRS694248
SRS693900
SRS693980
SRS693956
Thailand
SRS693269
SRS693583
SRS693908
SRS693348
Viet Nam
SRS693899
SRS258286
North Korea
Mauritania
SRS258091
SRS363193
Madagascar
SRS417747
SRS363192
SRS696222
SRS805942
India
SRS805922
Colombia
SRS693897
SRS693950
SRS694231
SRS693267
SRS694247
SRS693942
SRS696220
Peru
SRS693469
Nicaragua
SRS693266
Panama
SRS365051
El Salvador
SRS694245
SRS694229
SRS693273
Mexico
SRS694267
SRS694244
3636
2302
AF28
simium
AF33
AF1
AF30
AF36
AF22
SRS693307
SRS693354
SRS693476
SRS819436
SRS819493
SRS693364
SRS696216
Peru
SRS693569
SRS696214
AM02
SRS258178
SRS365050
AM01
Brazil
SRS693934
SRS693551
SRS693582
1.0E-4
Peru
Nicaragua
Panama
Colombia
El Salvador
Mexico
Asia
simium
Brazil
Peru

Figure S11

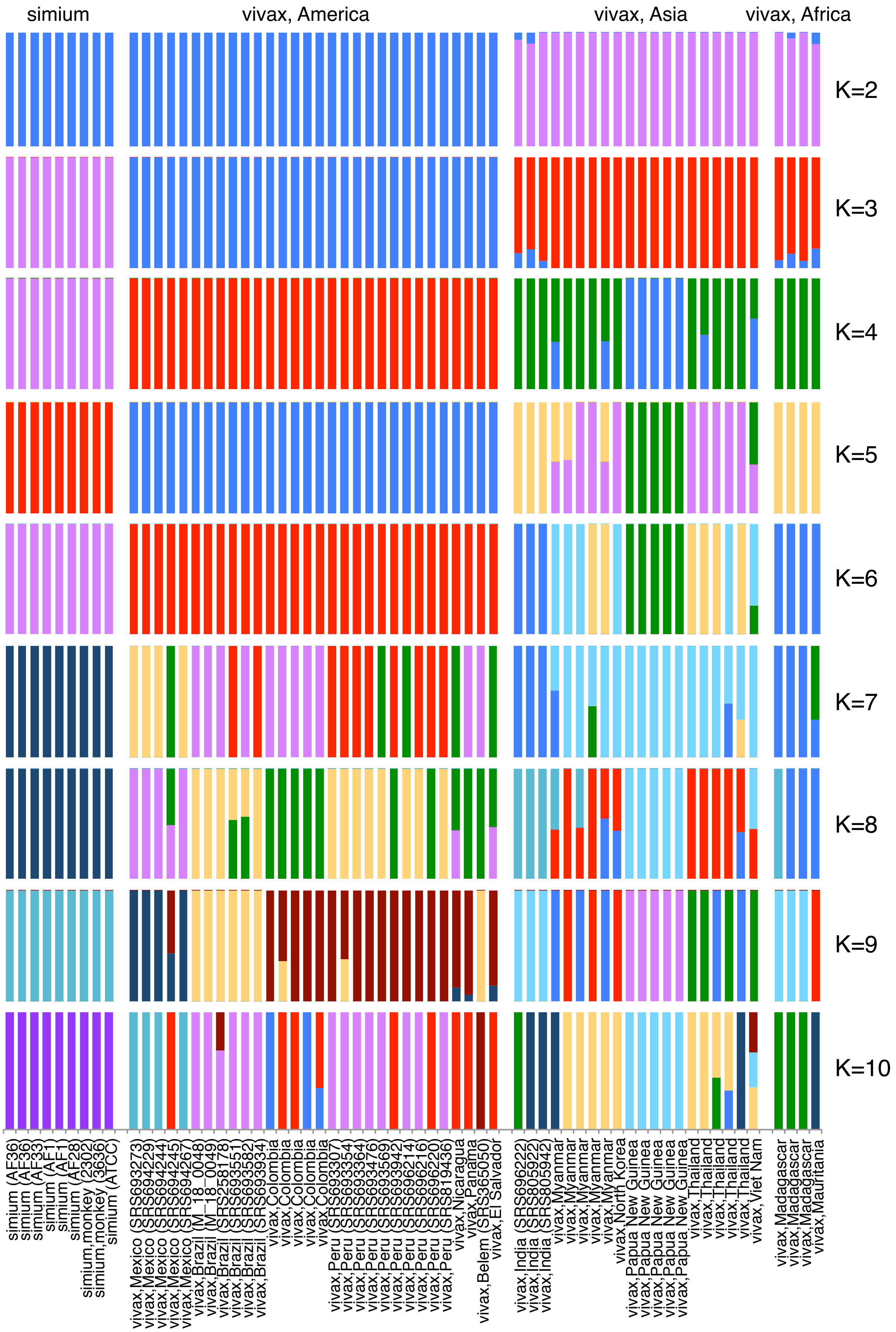
simium
vivax, America
vivax, Asia
vivax, Africa
K=2
K=3
K=4
K=5
K=6
K=7
K=8
K=9
K=10
simium (AF36)
simium (AF36)
simium (AF33)
simium (AF1)
simium (AF1)
simium (AF28)
simium,monkey (2302)
simium,monkey (3636)
simium (ATCC)
vivax,Mexico (SRS693273)
vivax,Mexico (SRS694229)
vivax,Mexico (SRS694244)
vivax,Mexico (SRS694245)
vivax,Mexico (SRS694267)
vivax,Brazil (M_18_0048)
vivax,Brazil (M_18_0049)
vivax,Brazil (SRS258178)
vivax,Brazil (SRS693551)
vivax,Brazil (SRS693582)
vivax,Brazil (SRS693934)
vivax,Colombia
vivax,Colombia
vivax,Colombia
vivax,Colombia
vivax,Peru (SRS693307)
vivax,Peru (SRS693354)
vivax,Peru (SRS693364)
vivax,Peru (SRS693476)
vivax,Peru (SRS693569)
vivax,Peru (SRS693942)
vivax,Peru (SRS696214)
vivax,Peru (SRS696216)
vivax,Peru (SRS696220)
vivax,Peru (SRS819436)
vivax,Nicaragua
vivax,Panama
vivax,Belem (SRS365050)
vivax,El Salvador
vivax,India (SRS696222)
vivax,India (SRS805922)
vivax,India (SRS805942)
vivax,Myanmar
vivax,Myanmar
vivax,Myanmar
vivax,Myanmar
vivax,Myanmar
vivax,North Korea
vivax,Papua New Guinea
vivax,Papua New Guinea
vivax,Papua New Guinea
vivax,Papua New Guinea
vivax,Papua New Guinea
vivax,Thailand
vivax,Thailand
vivax,Thailand
vivax,Thailand
vivax,Thailand
vivax,Viet Nam
vivax,Madagascar
vivax,Madagascar
vivax,Madagascar
vivax,Mauritania

Figure S12

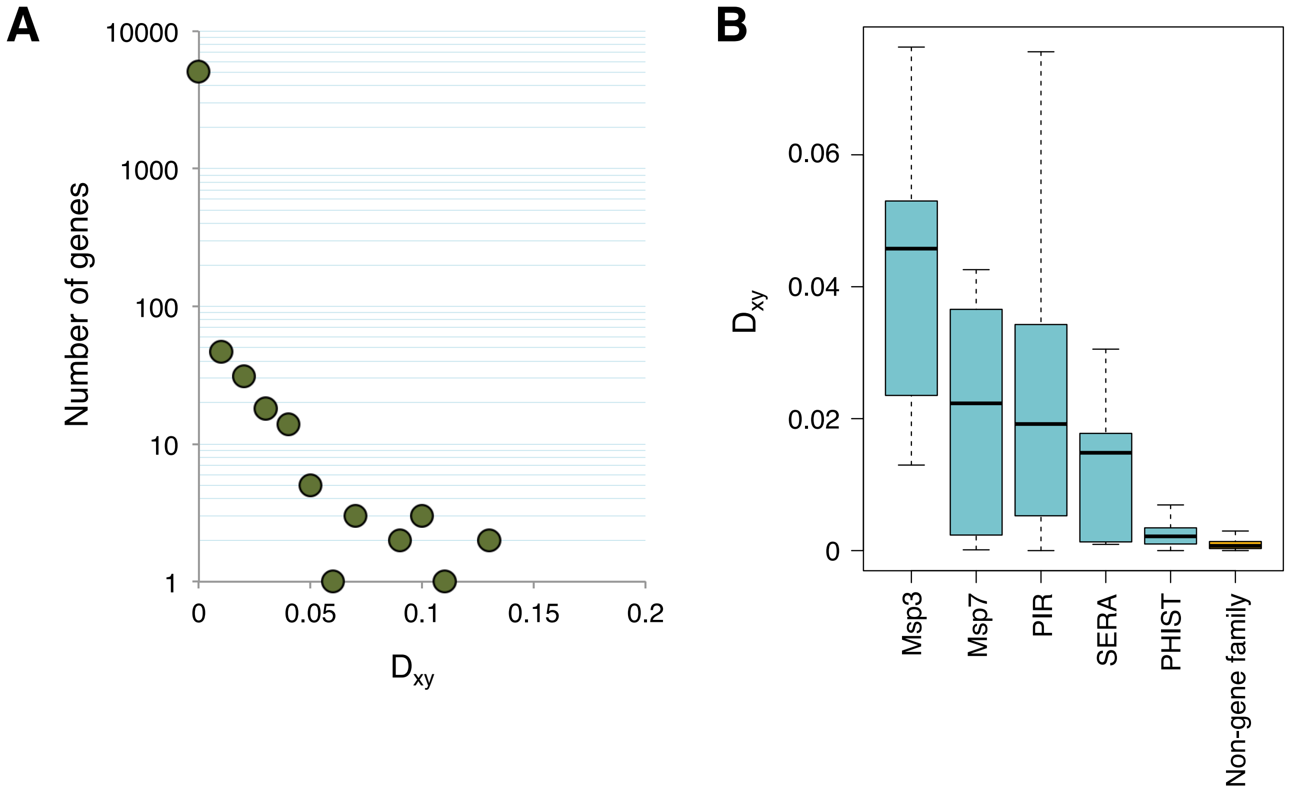
A
10000
1000
100
10
1
Number of genes
0
0.05
0.1
0.15
0.2
D_{xy}
B
0.06
0.04
0.02
0
D_{xy}
Msp3
Msp7
PIR
SERA
PHIST
Non-gene family

Figure S13

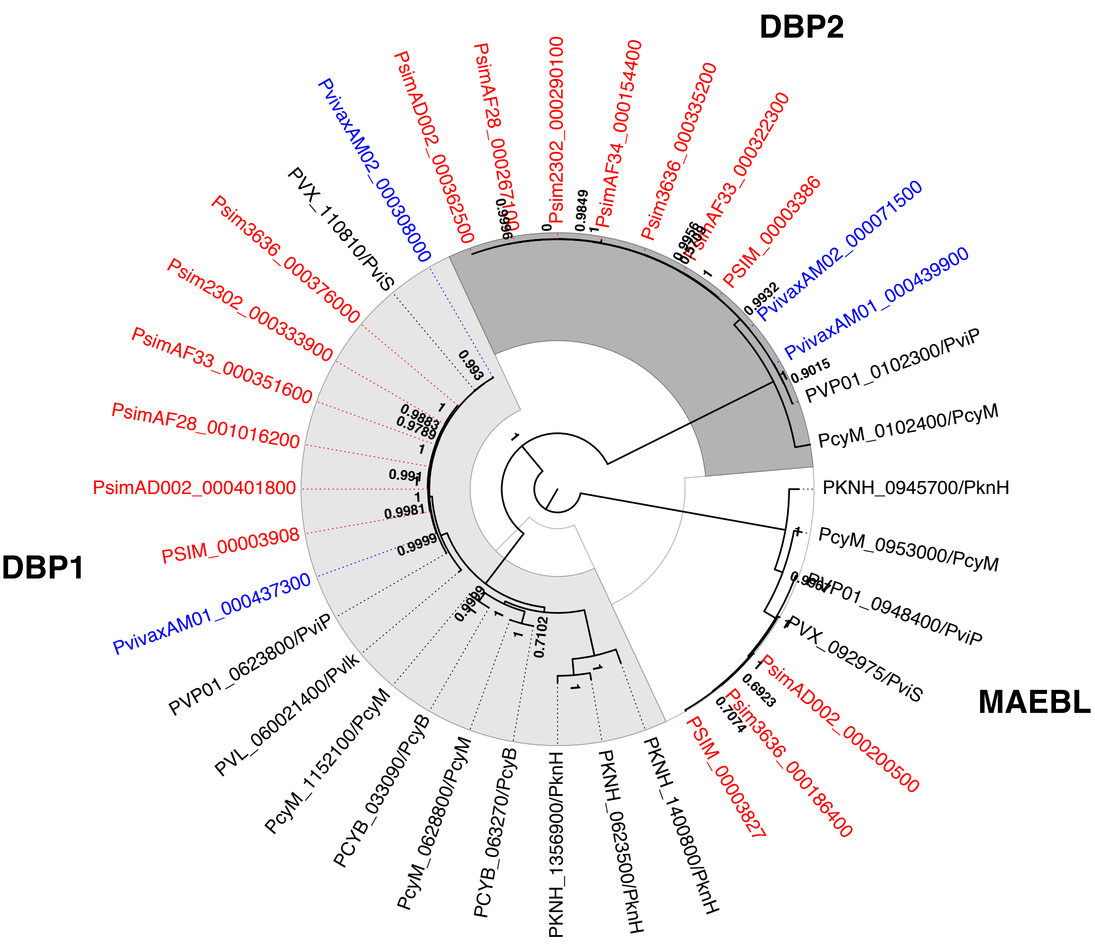
DBP2
DBP1
MAEBL
PvivaxAM02_000308000
PsimAD002_000362500
PsimAF28_000267100
Psim2302_000290100
PsimAF34_000154400
Psim3636_000335200
PsimAF33_000322300
PSIM_00003386
PvivaxAM02_000071500
PvivaxAM01_000439900
PVP01_0102300/PviP
PcyM_0102400/PcyM
PKNH_0945700/PknH
PcyM_0953000/PcyM
PVP01_0948400/PviP
PVX_092975/PviS
PsimAD002_000200500
Psim3636_000186400
PSIM_00003827
PKNH_1400800/PknH
PKNH_0623500/PknH
PKNH_1356900/PknH
PCYB_063270/PcyB
PcyM_0628800/PcyM
PCYB_033090/PcyB
PcyM_1152100/PcyM
PVL_060021400/PvIk
PVP01_0623800/PviP
PvivaxAM01_000437300
PSIM_00003908
PsimAD002_000401800
PsimAF28_001016200
PsimAF33_000351600
Psim2302_000333900
Psim3636_000376000
PVX_110810/PviS

Figure S14

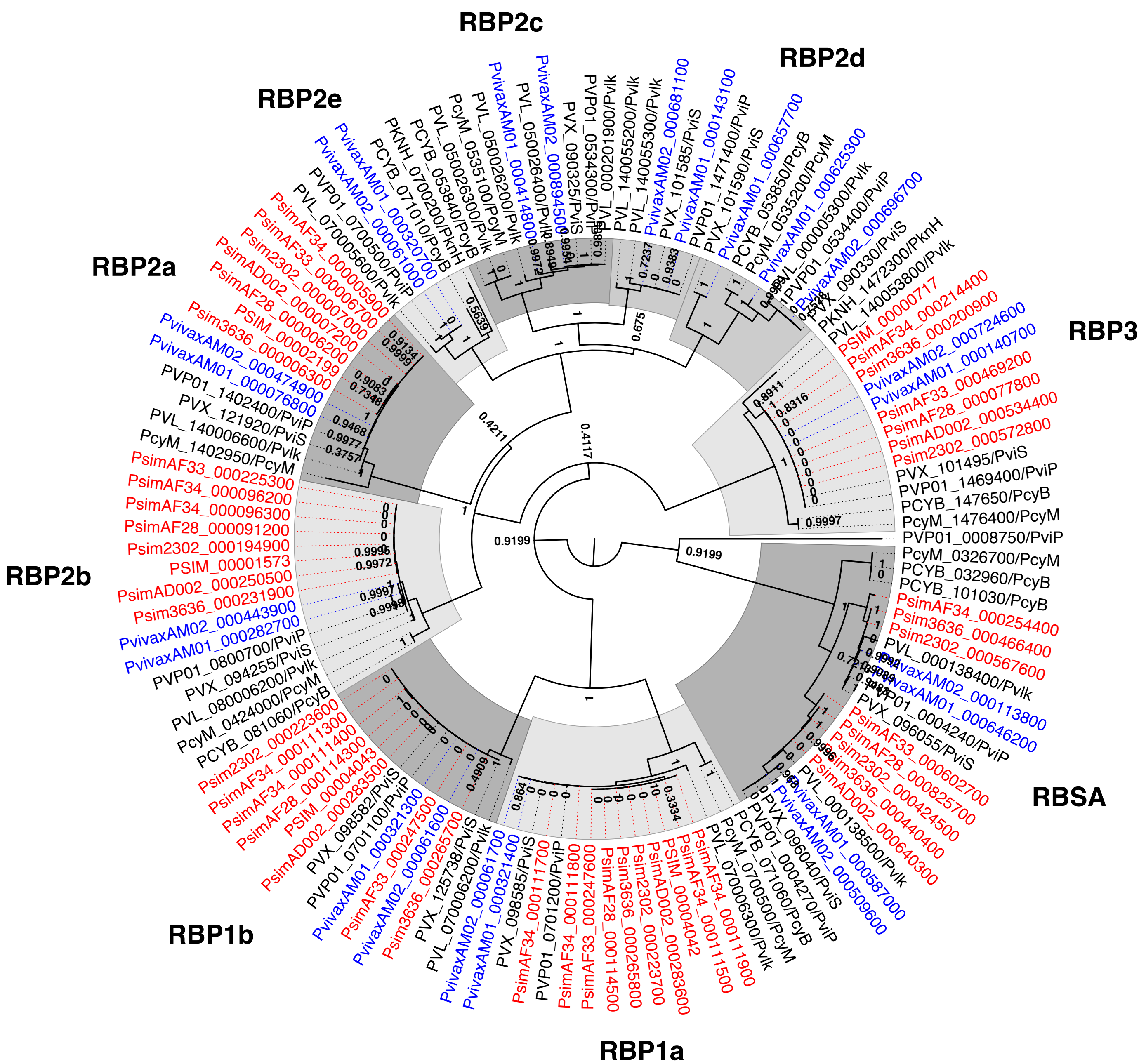

Figure S15

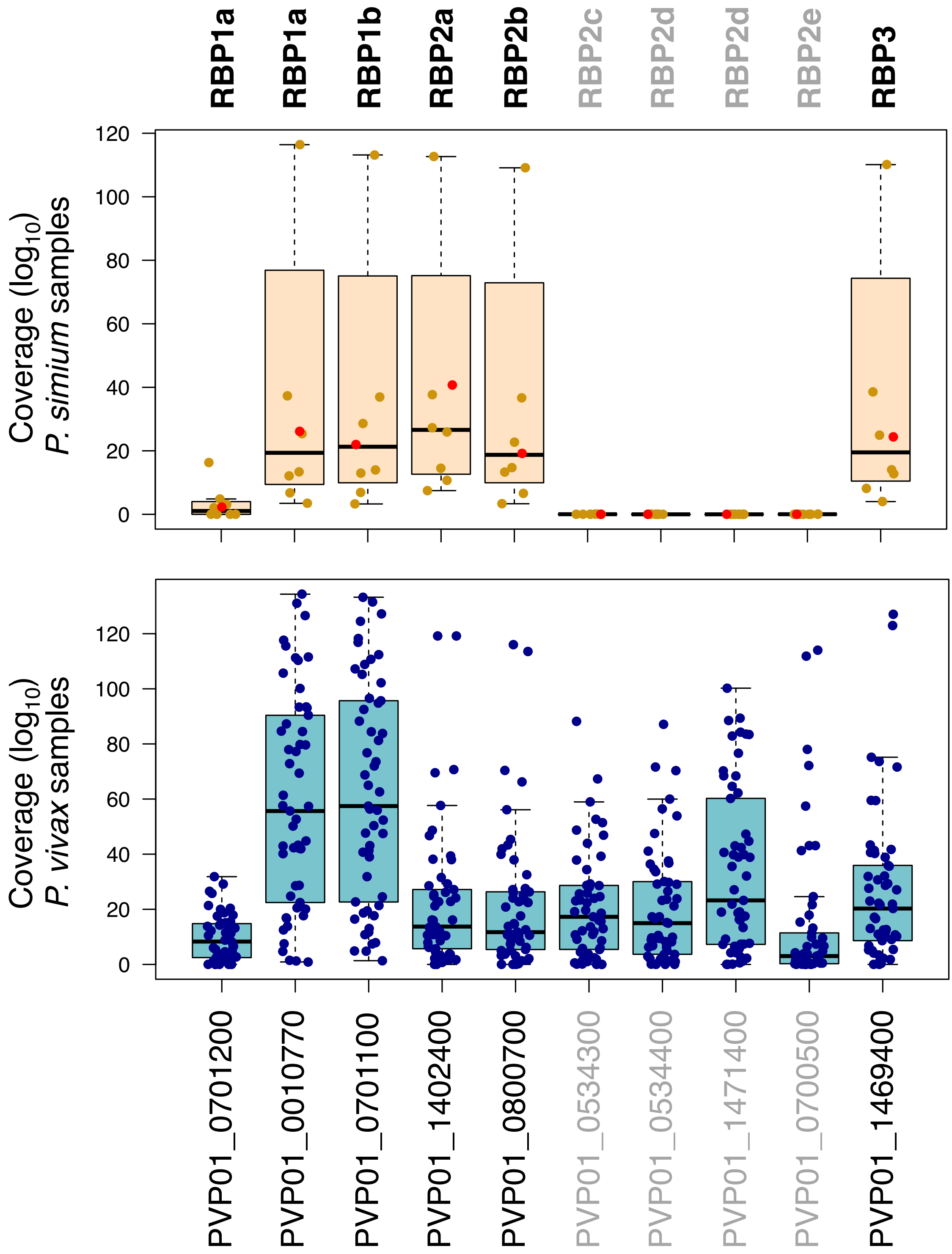

RBP1a
RBP1a
RBP1b
RBP2a
RBP2b
RBP2c
RBP2d
RBP2d
RBP2e
RBP3
Coverage (log10)
P. simium samples
Coverage (log10)
P. vivax samples
PVP01_0701200
PVP01_0010770
PVP01_0701100
PVP01_1402400
PVP01_0800700
PVP01_0534300
PVP01_0534400
PVP01_1471400
PVP01_0700500
PVP01_1469400

Figure S16

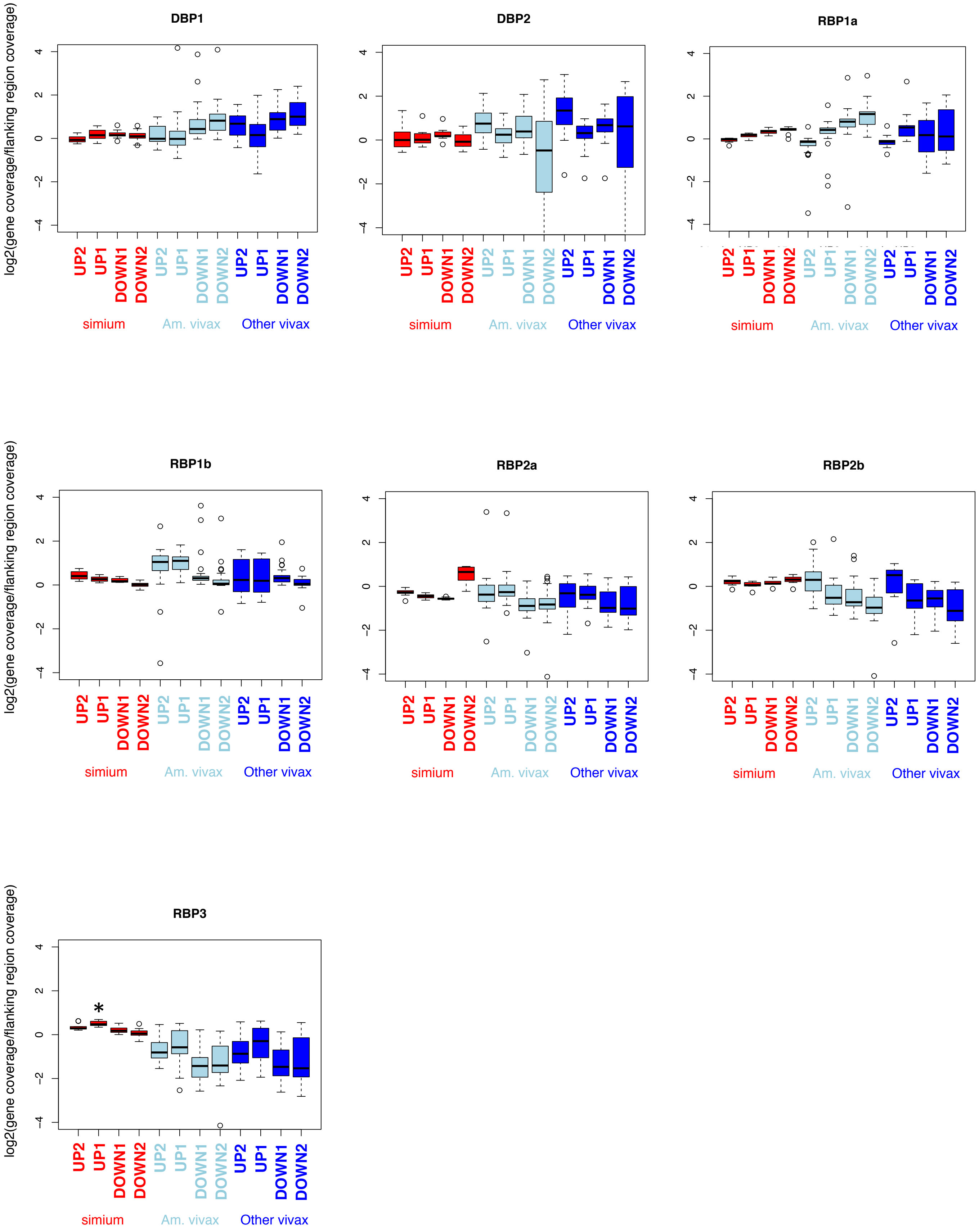
DBP1
DBP2
RBP1a
RBP1b
RBP2a
RBP2b
RBP3
log2(gene coverage/flanking region coverage)
4
2
0
-2
-4
UP2
UP1
DOWN1
DOWN2
simium
Am. vivax
Other vivax
*

Figure S18

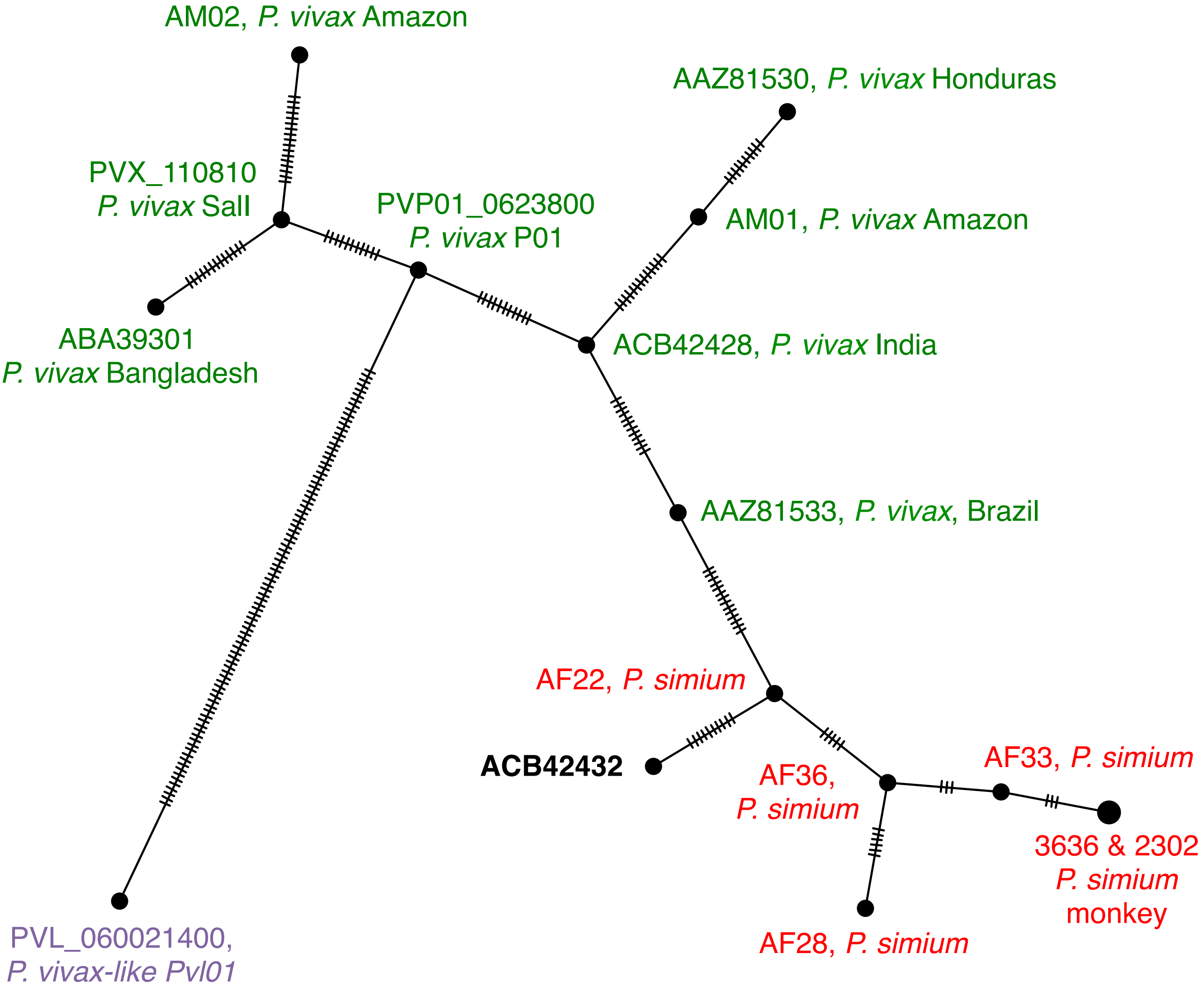

AM02, *P. vivax* Amazon
AAZ81530, *P. vivax* Honduras
PVX_110810
P. vivax SalI
PVP01_0623800
P. vivax P01
AM01, *P. vivax* Amazon
ABA39301
P. vivax Bangladesh
ACB42428, *P. vivax* India
AAZ81533, *P. vivax*, Brazil
AF22, *P. simium*
ACB42432
AF36,
P. simium
AF33, *P. simium*
3636 & 2302
P. simium
monkey
AF28, *P. simium*
PVL_060021400,
P. vivax-like Pvl01

Figure S19

```
simium           GGTAACCCCCTATAATGGTTT------------------------------------------------------------------------------------------------------------------GGGGCAAGATAATGAT
vivax 1, P01     GGTAACCCCCTATAATGGTTTGGGGCATTCGAAAGACAATAGTGATAGCGATGGACCTGCGGAATTTGCAGAATCTACGAAATCTGCGGAATCAATGGCGAATCCTGATTCAAATAGTAAAGGTGAGACGGGAAAGGGGCAAGATAATGAT
vivax 2, SalI    GGTAACCCCCTATAATGGTTTGGGGCATTCGAAAGACAATAGTGATAGCGATGGAC---------------------------CTGCGGAATCAATGGCGAATCCTGATTCAAATAGTAAAGGTGAGACGGGAAAGGGGCAAGATAATGAT
vivax 3,         GGTAACCCCCTATAATGGTTTGGGGCATTCGAAAGACAATAGT------GATGGACCTGCGGAATTTGCAGAATCTACGAAATCTGCGGAATCAATGGCGAATCCTGATTCAAATAGTAAAGGTGAGACGGGAAAGGGGCAAGATAATGAT
vivax 4          GGTAACCCCCTATAATGGTTTGGGGCATTCGAAAGACAATAGT------GATGGAC---------------------------CTGCGGAATCAATGGCGAATCCTGATTCAAATAGTAAAGGTGAGACGGGAAAGGGGCAAGATAATGAT
```

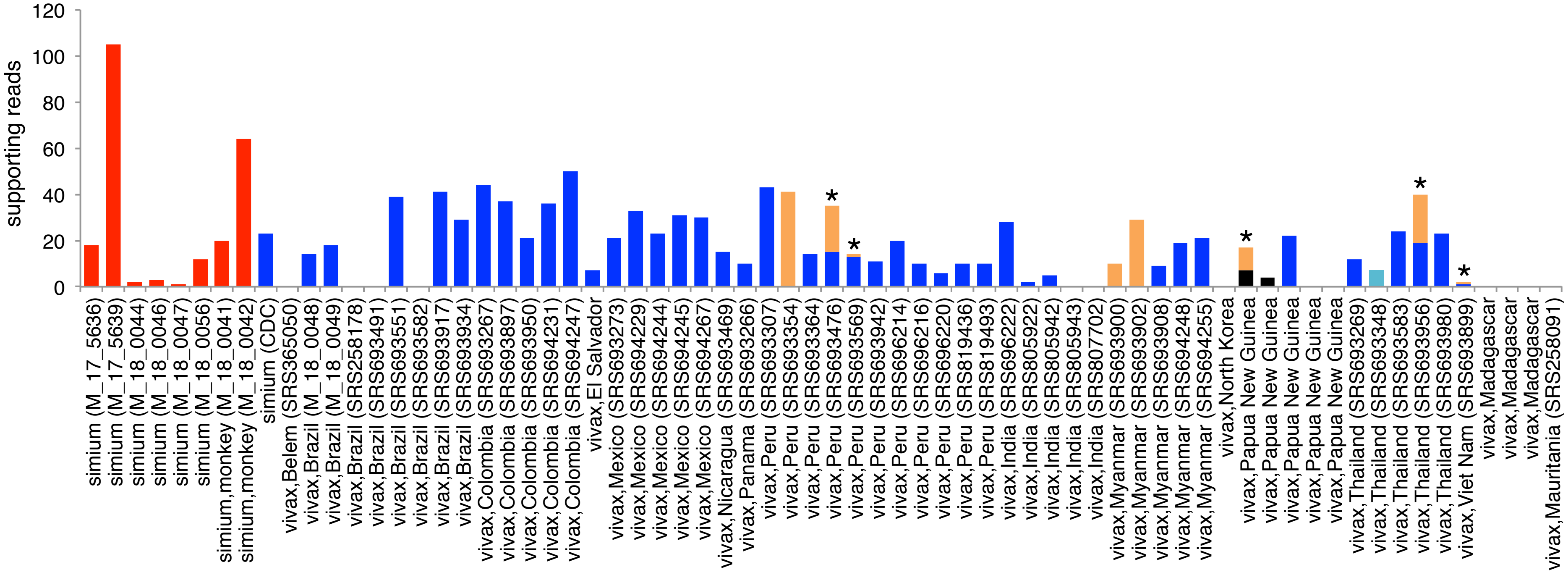

Figure S20

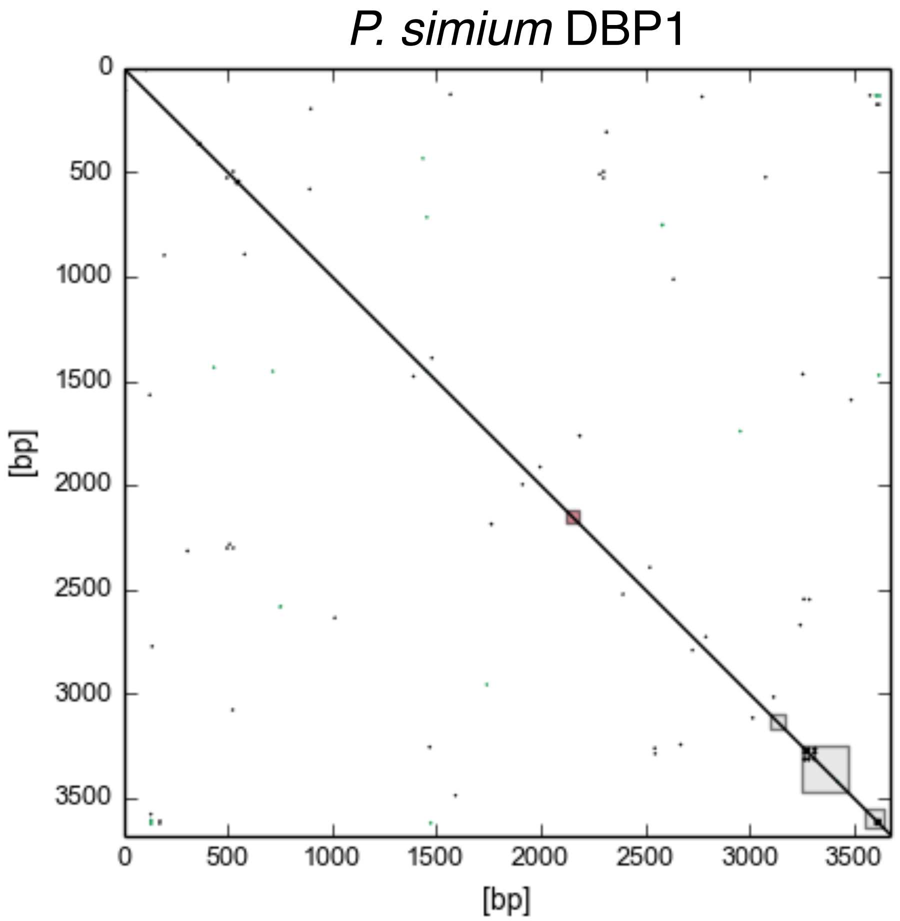
P. simium DBP1
[bp]
[bp]
0
500
1000
1500
2000
2500
3000
3500

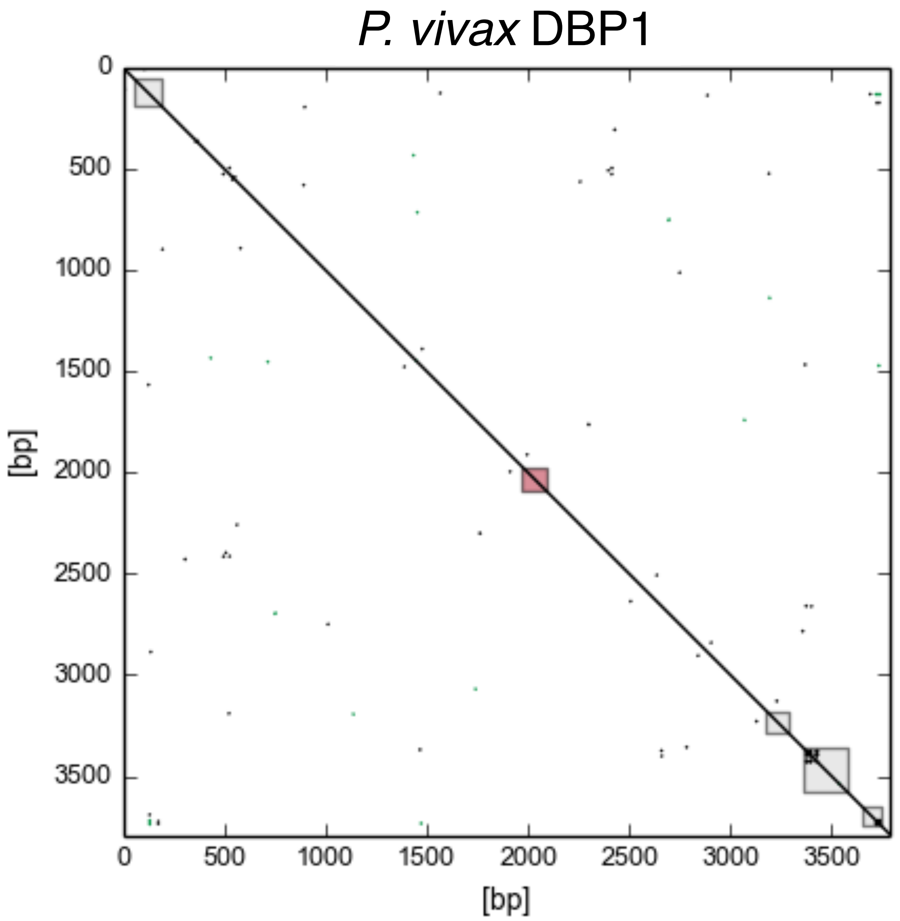
P. vivax DBP1
[bp]
[bp]
0
500
1000
1500
2000
2500
3000
3500

Figure S21

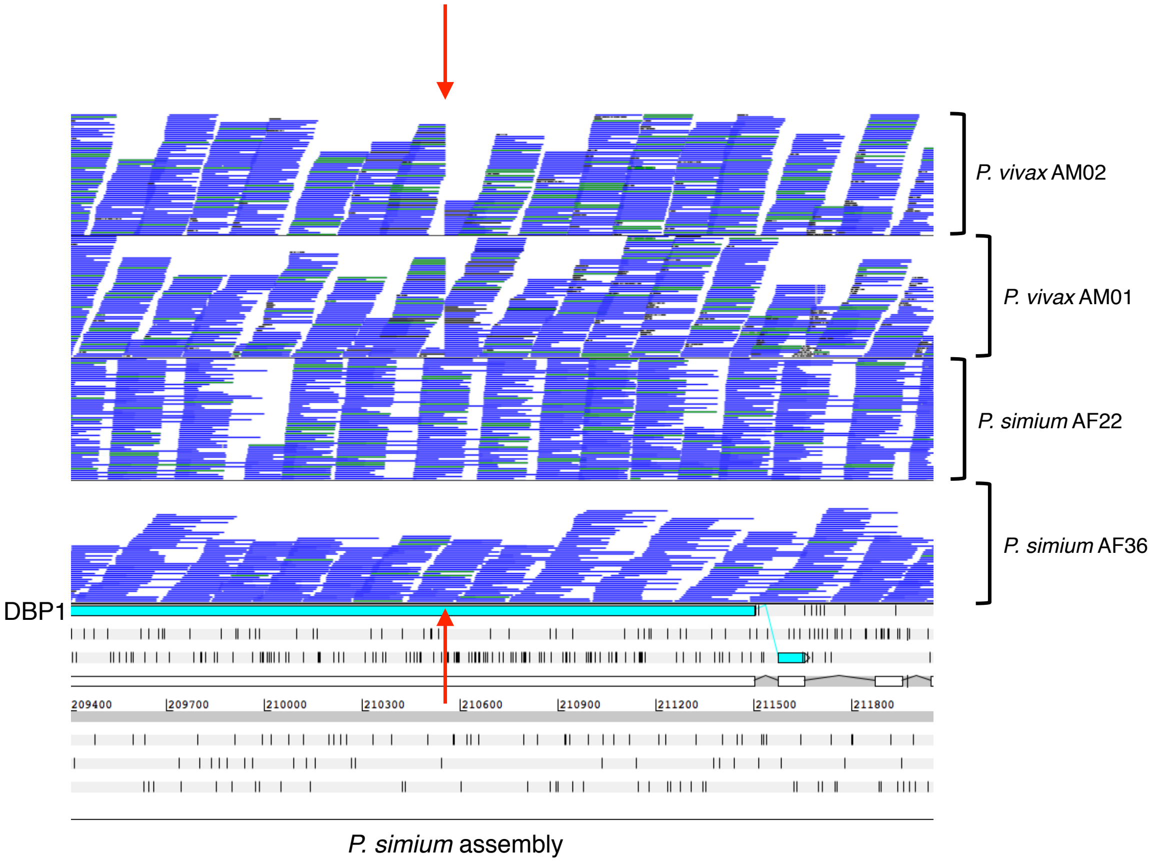

P. vivax AM02
P. vivax AM01
P. simium AF22
P. simium AF36
DBP1
209400
209700
210000
210300
210600
210900
211200
211500
211800
P. simium assembly

Figure S22

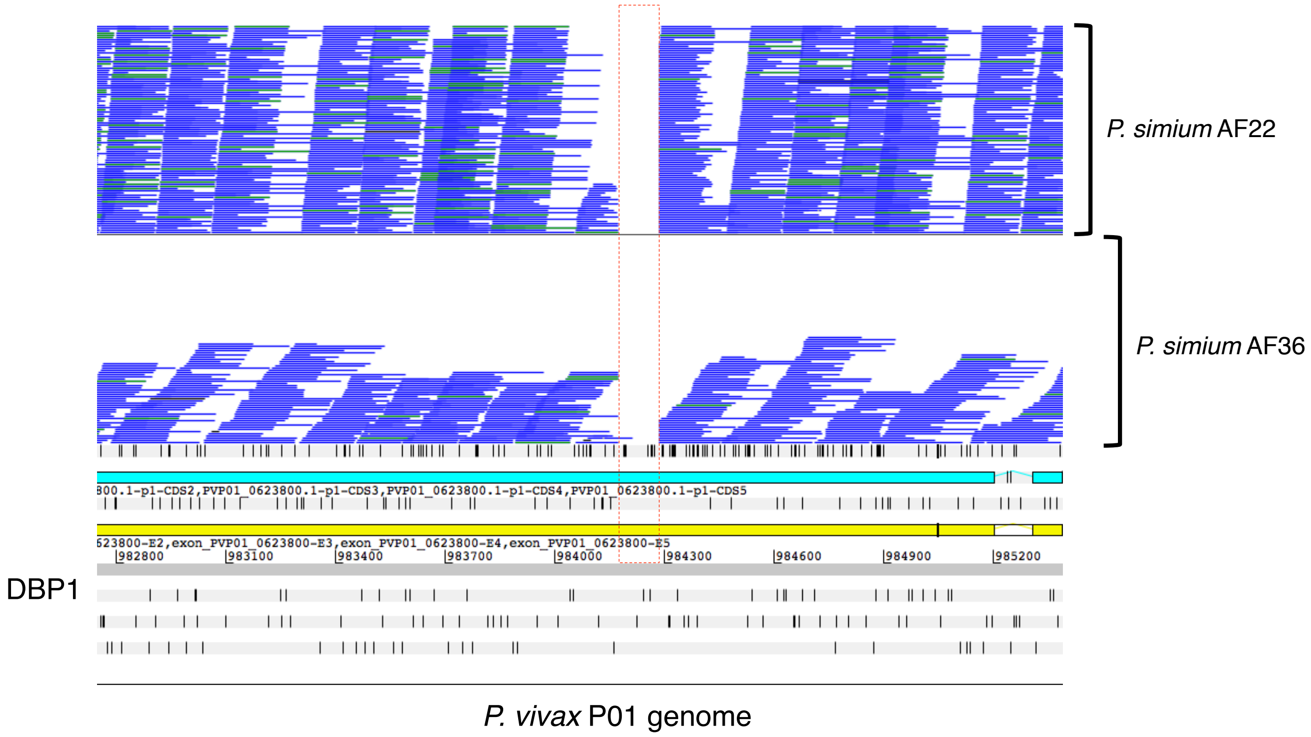

Figure S23

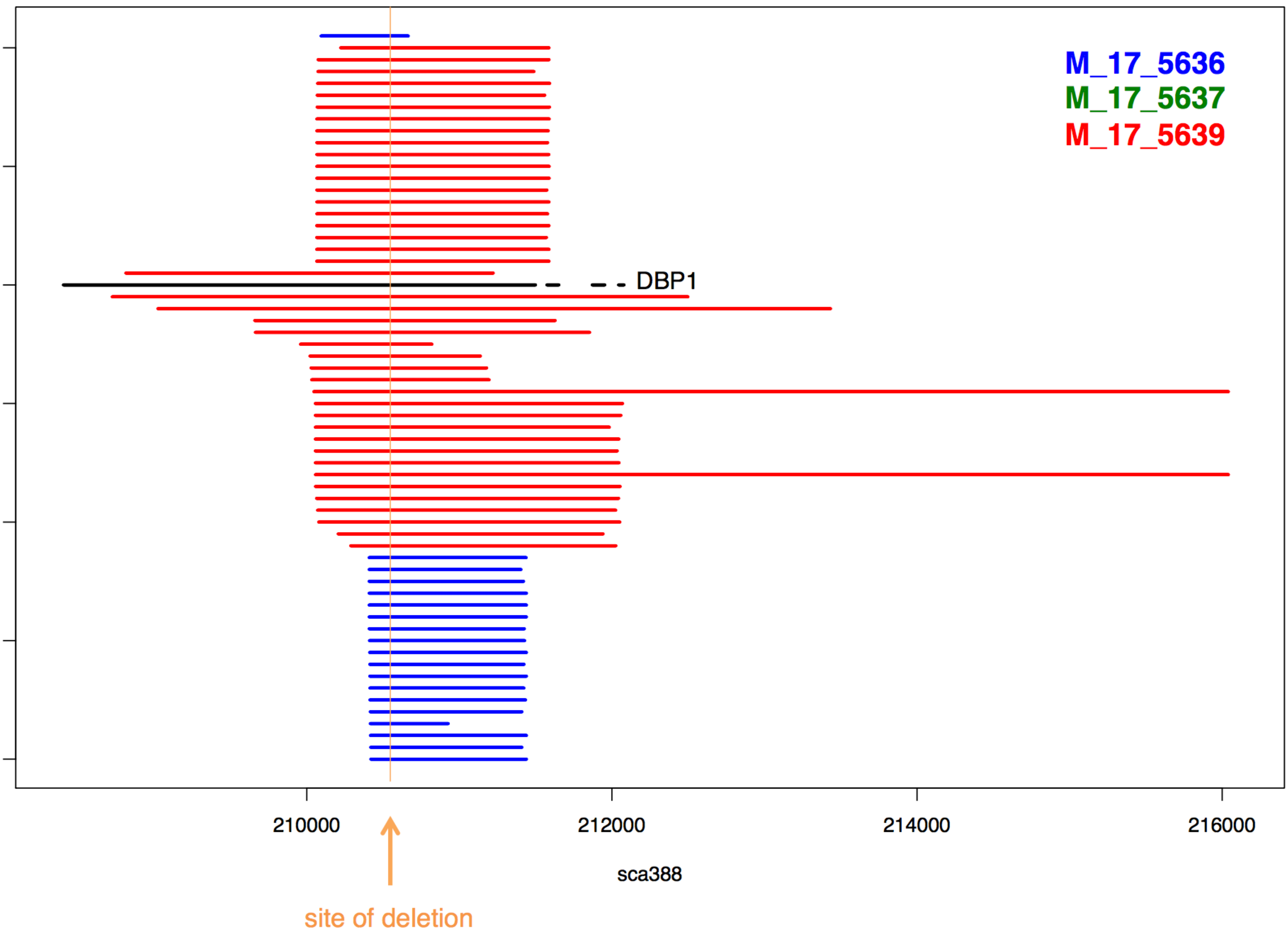

M_17_5636
M_17_5637
M_17_5639
DBP1
210000
212000
214000
216000
sca388
site of deletion

Figure S24

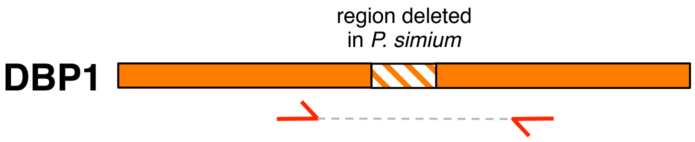

Human cases of *P. vivax*

Expected
band sizes:

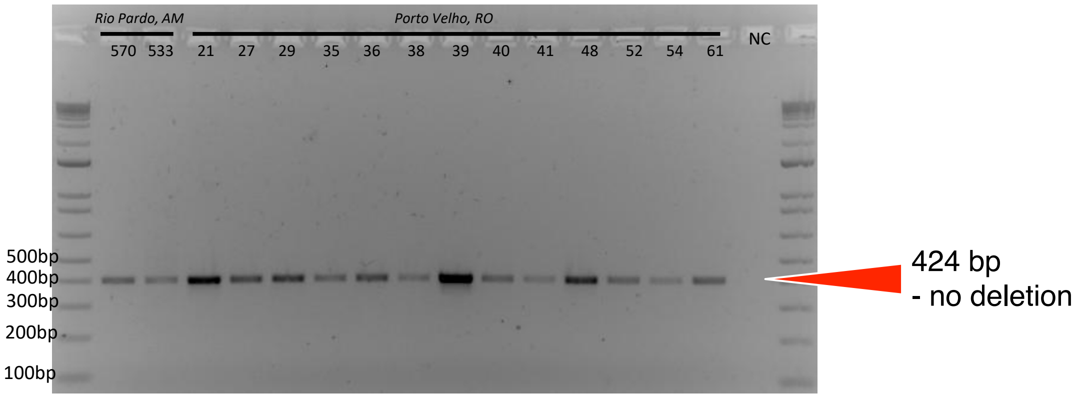

Human cases of *P. simium*

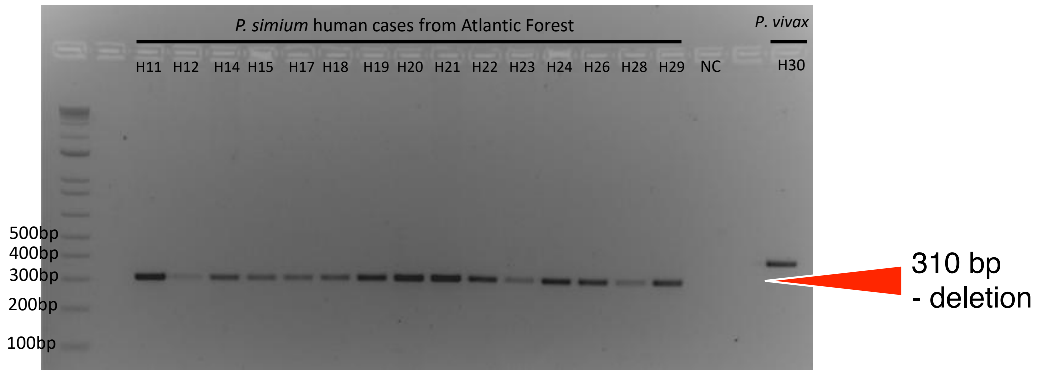

***P. simium* from NHPs**

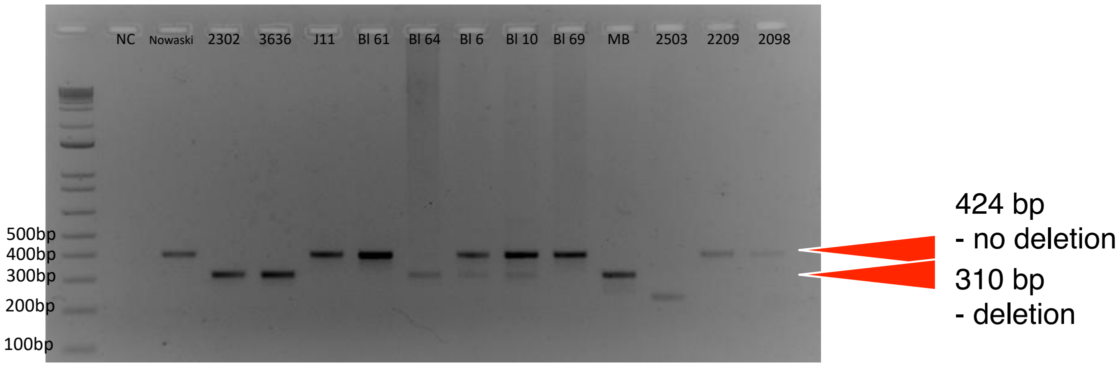

forward primer: ATCGCGACAAAGCTACTGCC
reverse primer: TCCCACAACAATCCCTGCTG

Figure S26

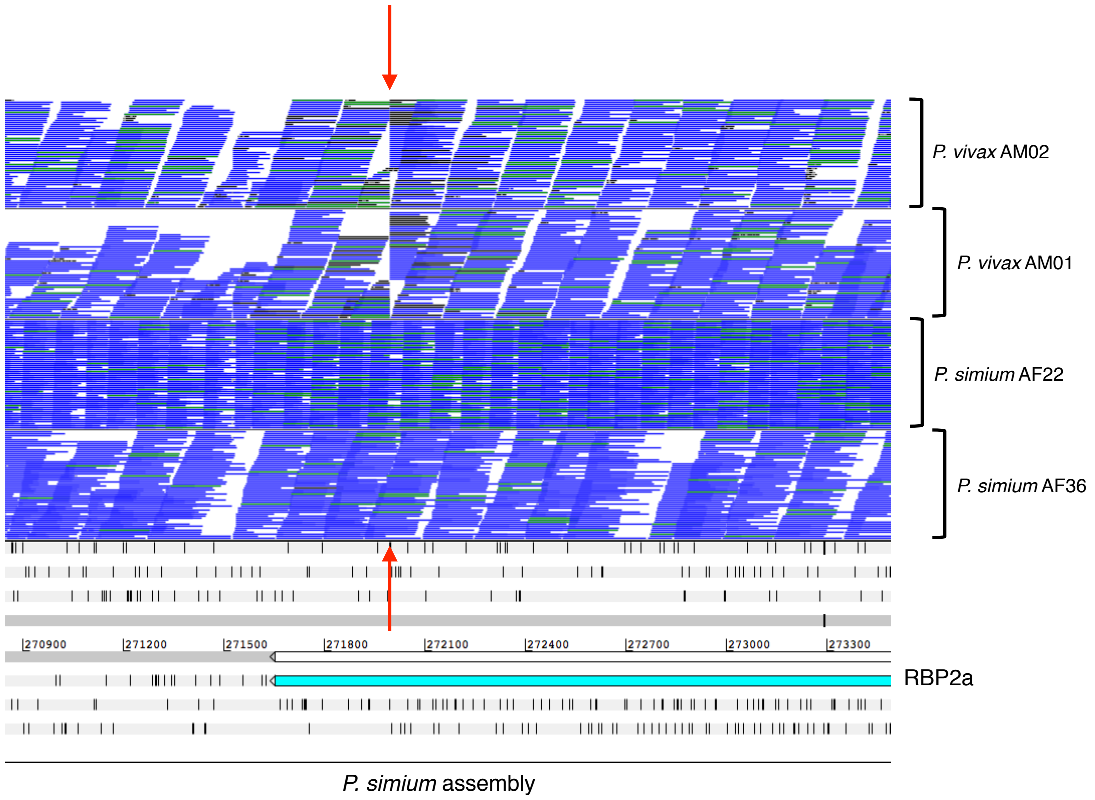
P. vivax AM02
P. vivax AM01
P. simium AF22
P. simium AF36
270900
271200
271500
271800
272100
272400
272700
273000
273300
RBP2a
P. simium assembly

Figure S27

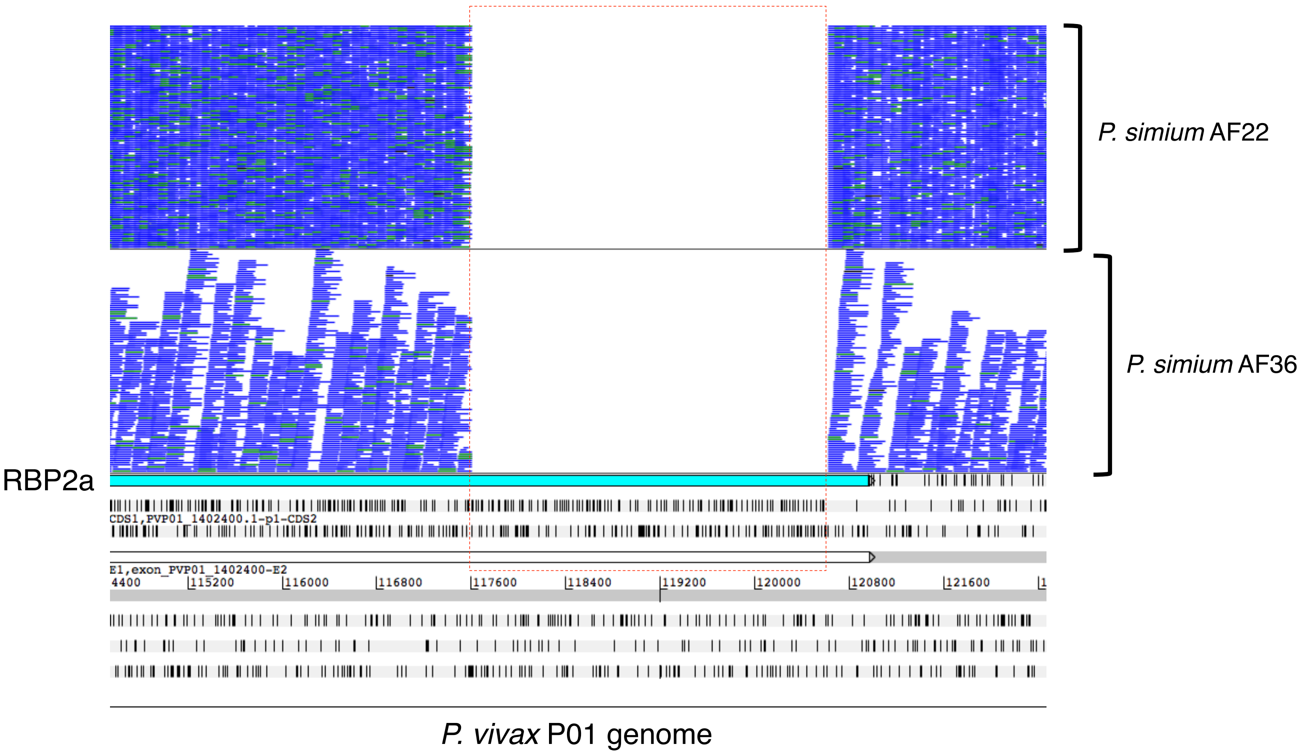
P. simium AF22
P. simium AF36
RBP2a
CDS1,PVP01_1402400.1-p1-CDS2
E1,exon_PVP01_1402400-E2
4400
115200
116000
116800
117600
118400
119200
120000
120800
121600
P. vivax P01 genome

Figure S28

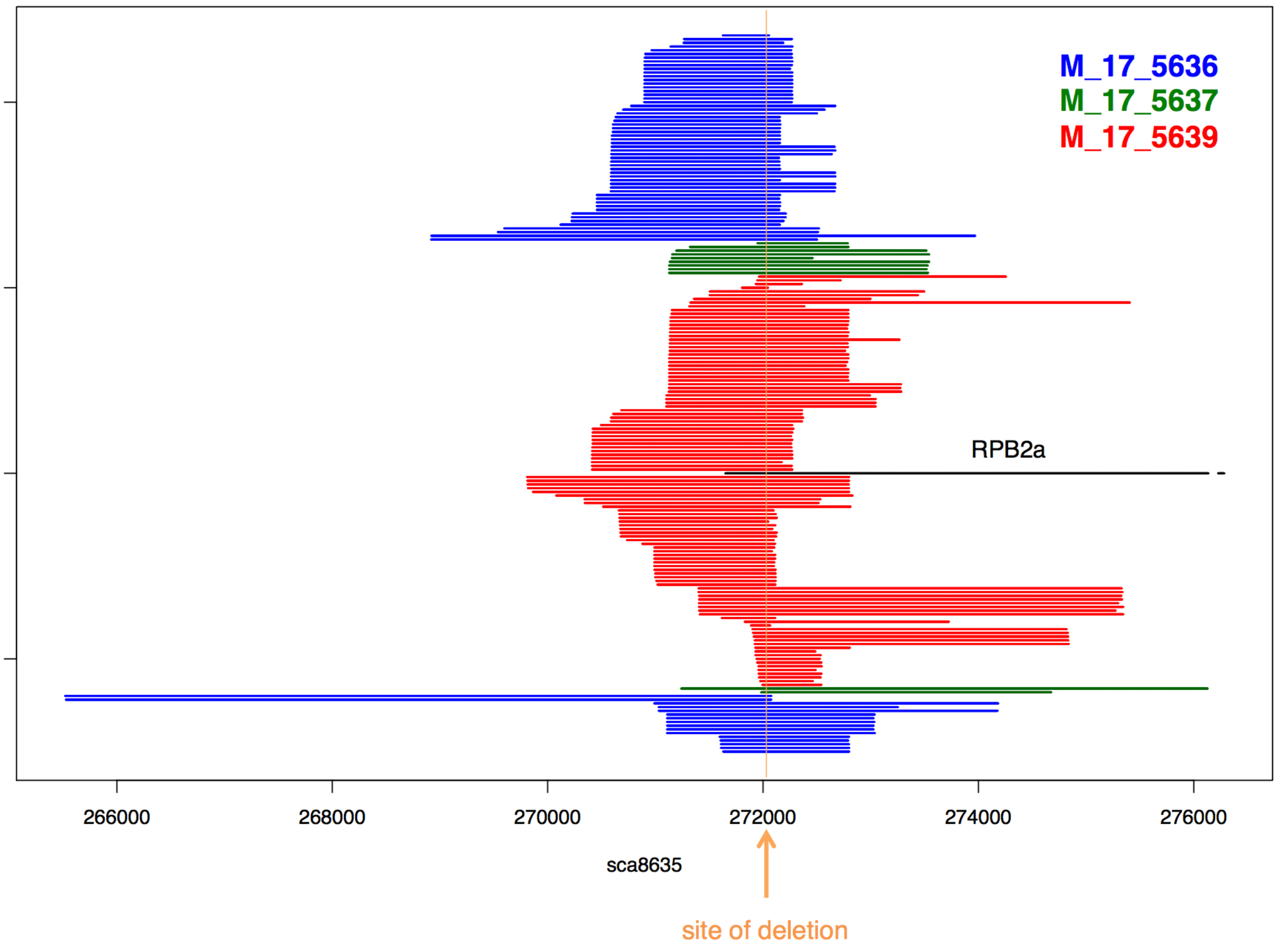
M_17_5636
M_17_5637
M_17_5639
RPB2a
266000
268000
270000
272000
274000
276000
sca8635
site of deletion

Figure S29

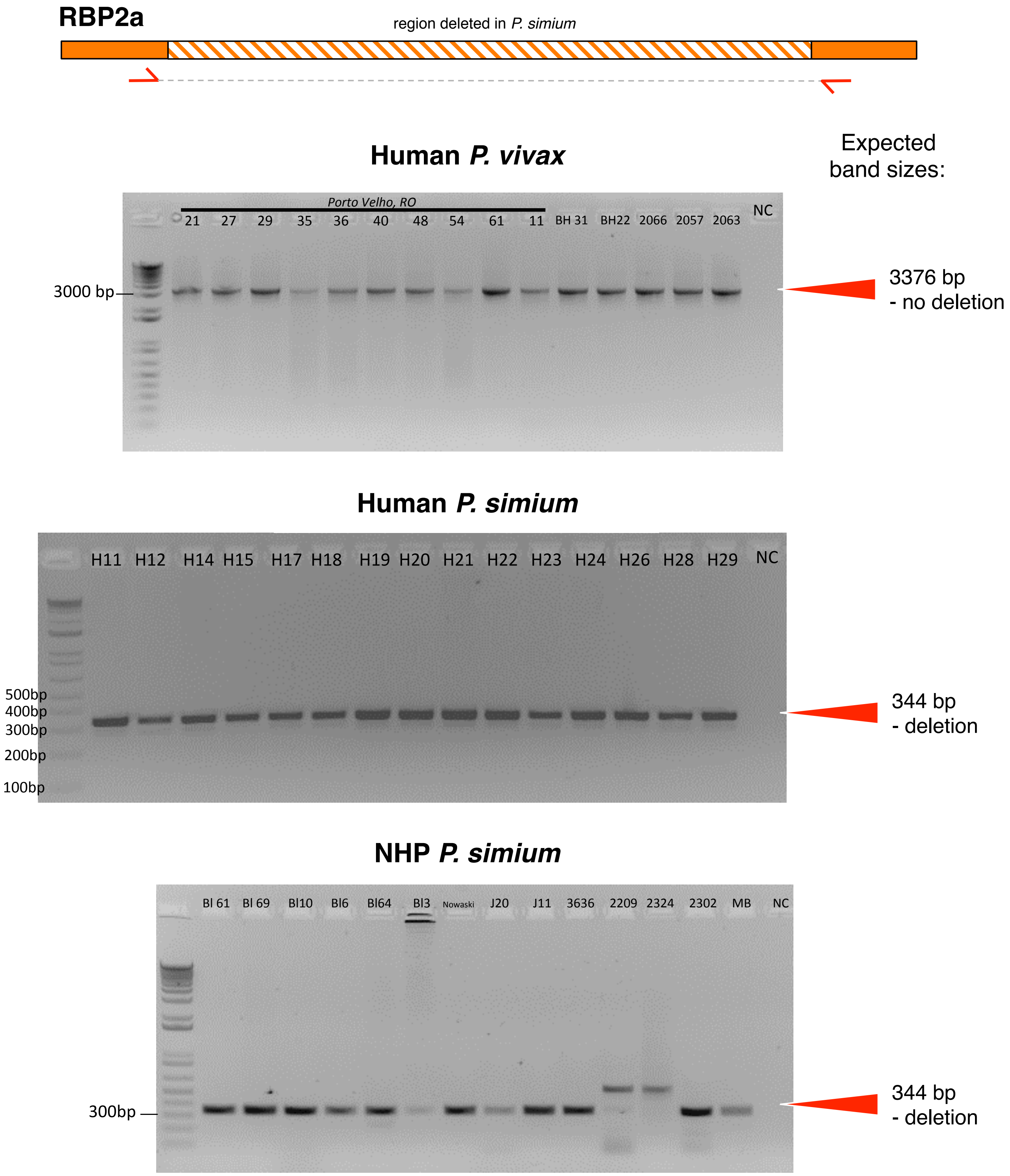

forward primer: AGTGCAAAAGCAGAGGAGG
reverse primer: CCTGCTGCAAAACGGACATT

Figure S30

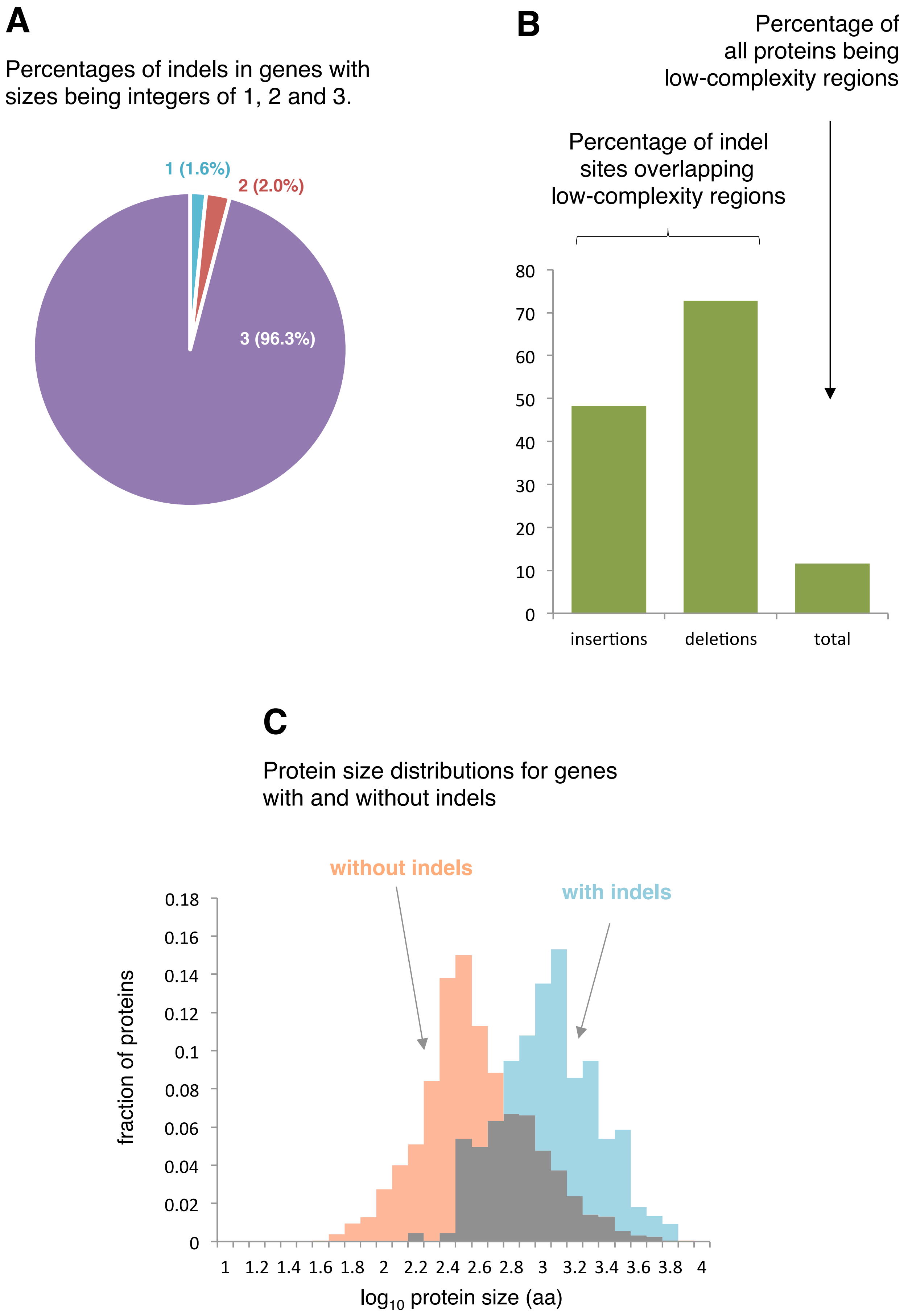
A
Percentages of indels in genes with sizes being integers of 1, 2 and 3.
1 (1.6%)
2 (2.0%)
3 (96.3%)
B
Percentage of all proteins being low-complexity regions
Percentage of indel sites overlapping low-complexity regions
80
70
60
50
40
30
20
10
0
insertions
deletions
total
C
Protein size distributions for genes with and without indels
without indels
with indels
0.18
0.16
0.14
0.12
0.1
0.08
0.06
0.04
0.02
0
fraction of proteins
1
1.2
1.4
1.6
1.8
2
2.2
2.4
2.6
2.8
3
3.2
3.4
3.6
3.8
4
log10 protein size (aa)

Figure S31

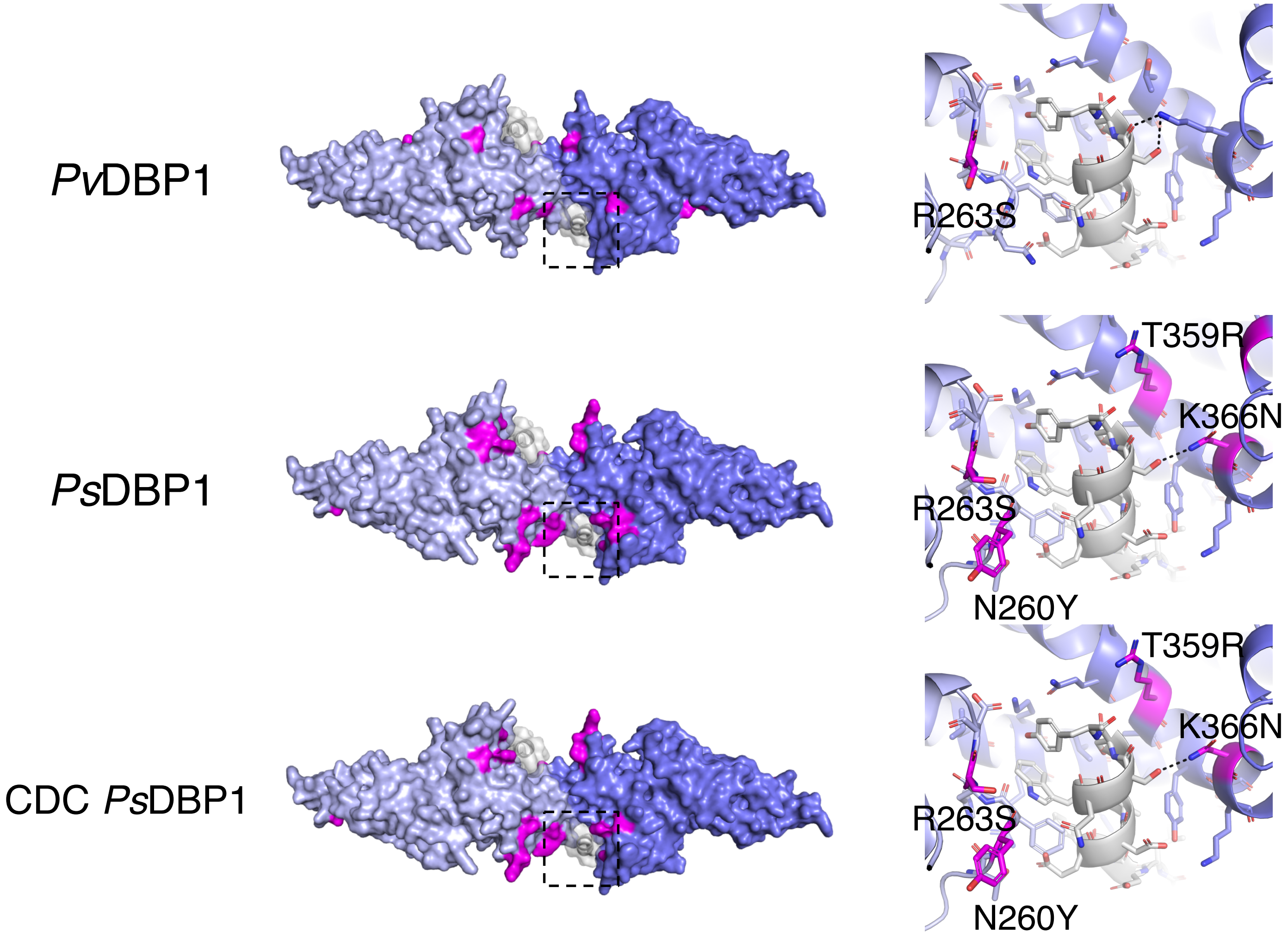

*Pv*DBP1
*Ps*DBP1
CDC *Ps*DBP1
R263S
T359R
K366N
R263S
N260Y
T359R
K366N
R263S
N260Y

Figure S32

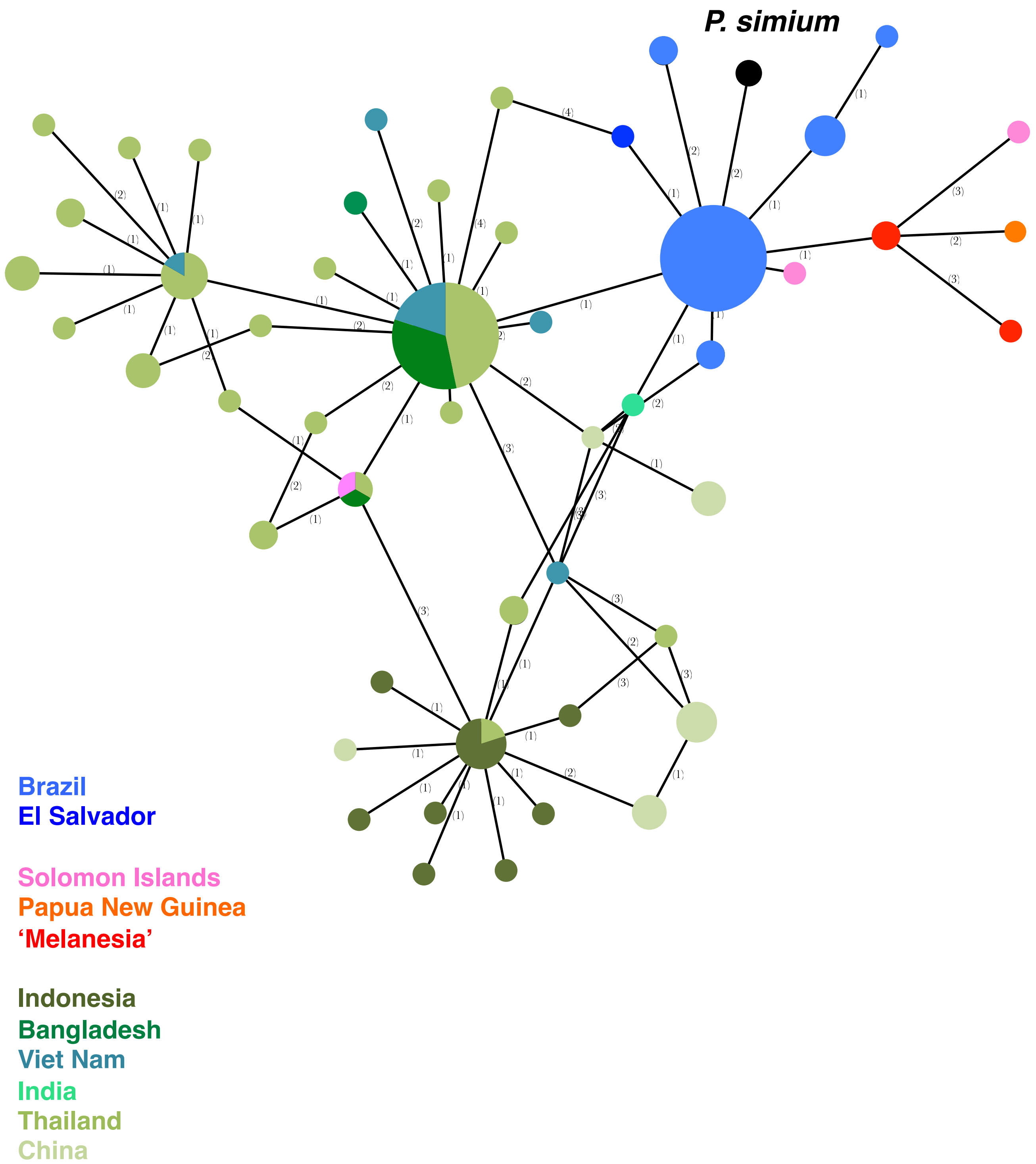

P. simium
Brazil
El Salvador
Solomon Islands
Papua New Guinea
'Melanesia'
Indonesia
Bangladesh
Viet Nam
India
Thailand
China

Figure S33

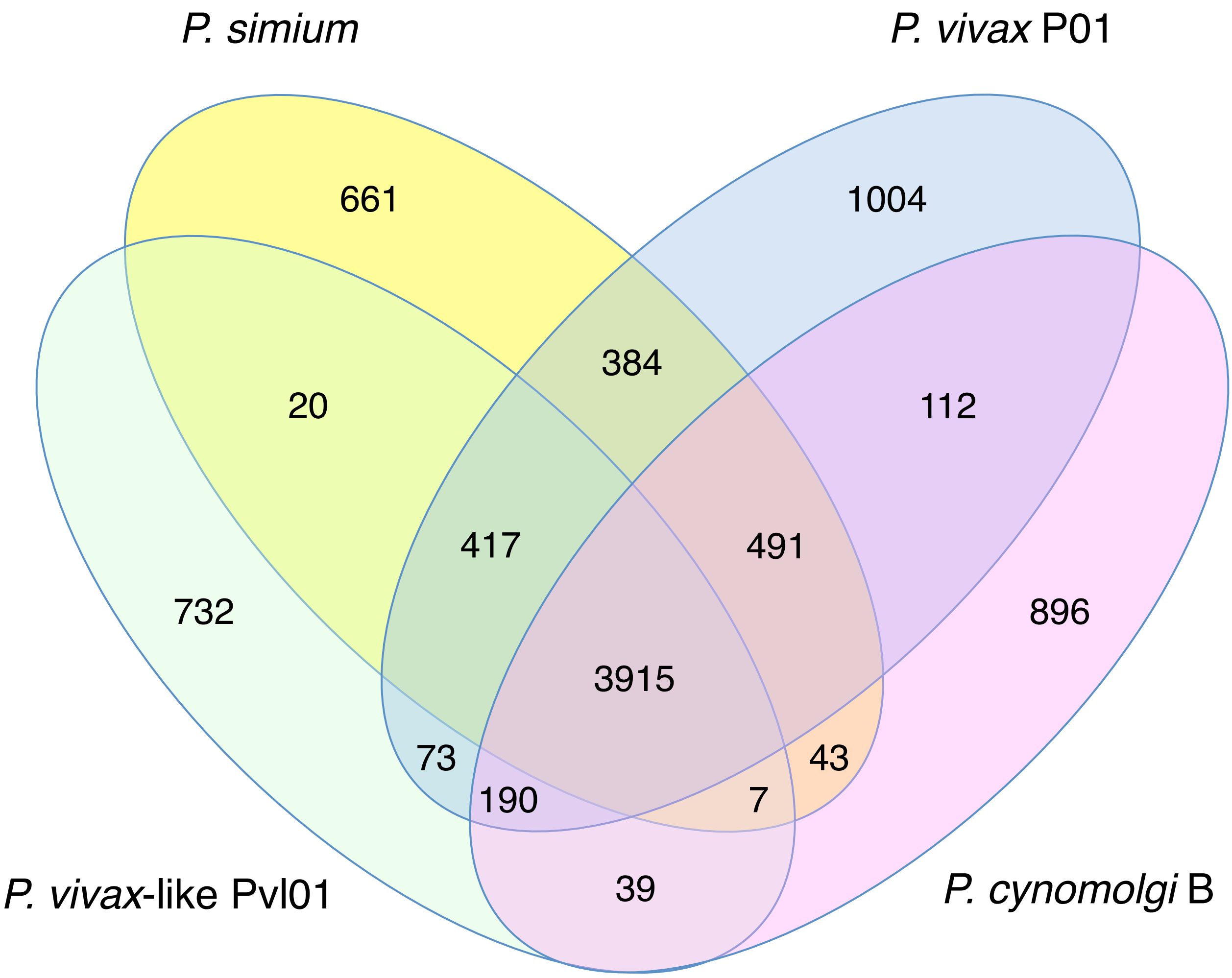
P. simium
P. vivax P01
P. vivax-like PvI01
P. cynomolgi B
661
1004
384
20
112
417
491
732
896
3915
73
43
190
7
39

Figure S34

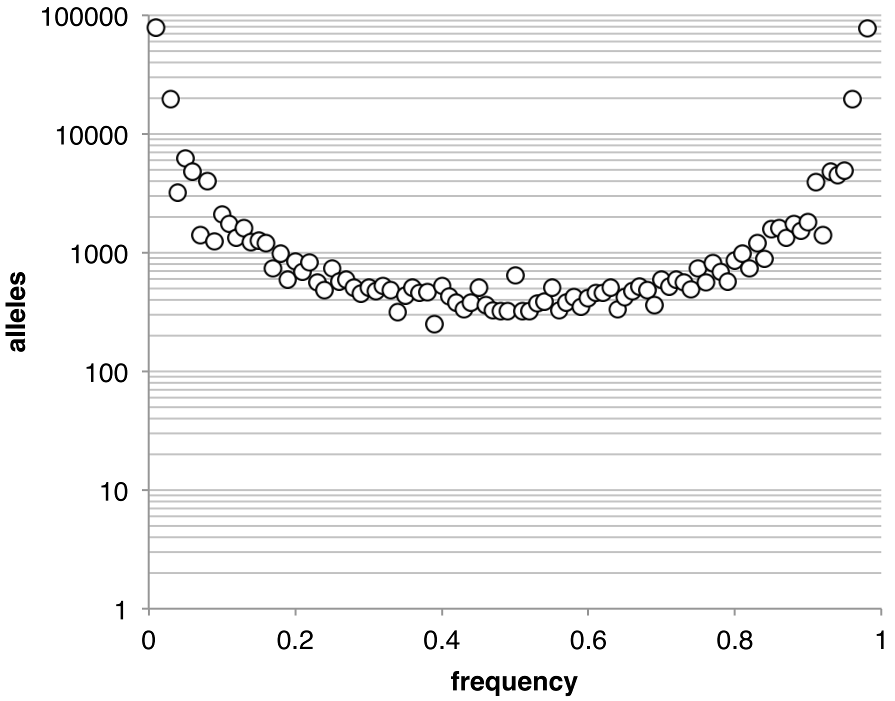

alleles
frequency
100000
10000
1000
100
10
1
0
0.2
0.4
0.6
0.8
1

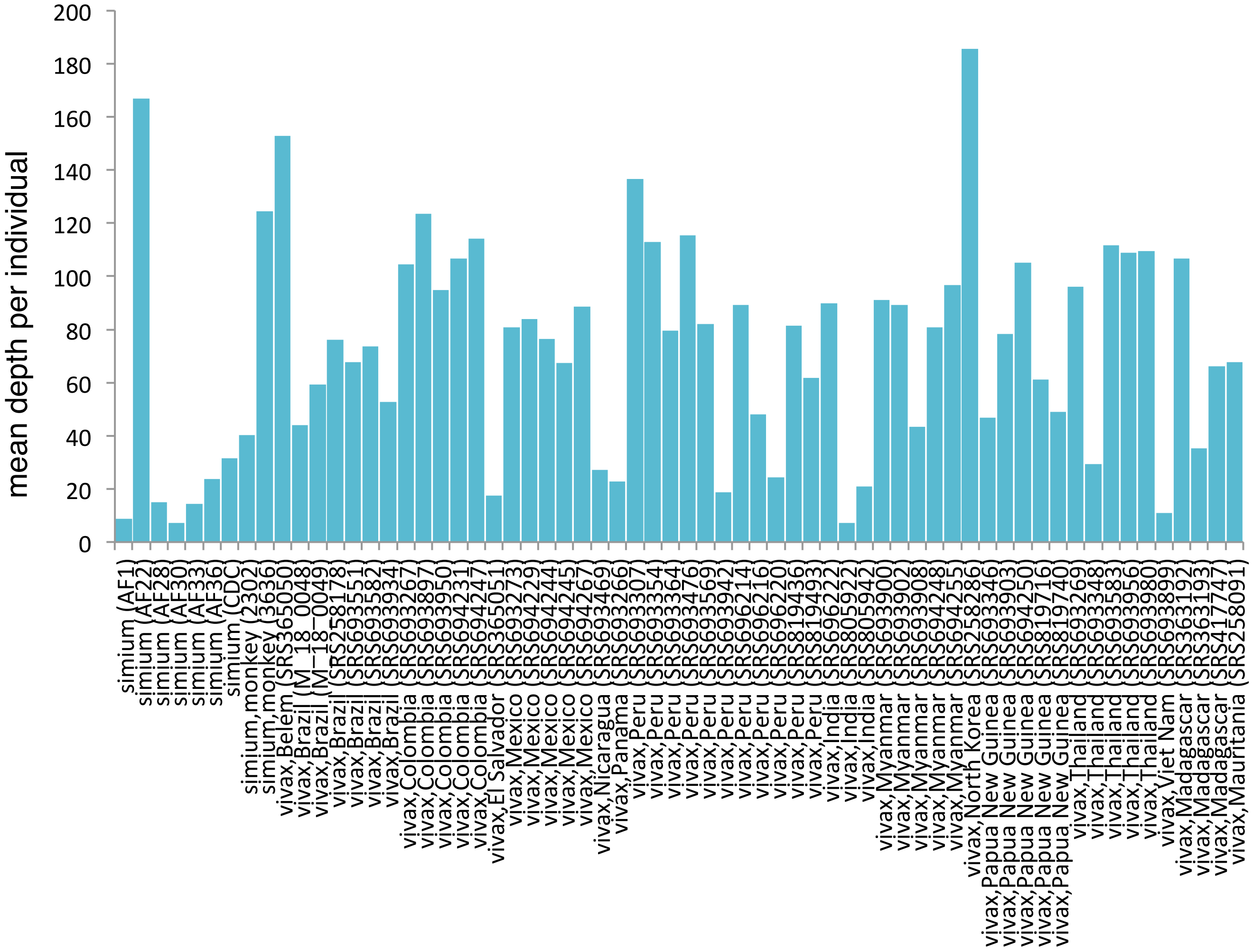

mean depth per individual
200
180
160
140
120
100
80
60
40
20
0
simium (AF1)
simium (AF22)
simium (AF28)
simium (AF30)
simium (AF33)
simium (AF36)
simium (CDC)
simium,monkey (2302)
simium,monkey (3636)
vivax,Belem (SRS365050)
vivax,Brazil (M_18_0048)
vivax,Brazil (M_18_0049)
vivax,Brazil (SRS258178)
vivax,Brazil (SRS693551)
vivax,Brazil (SRS693582)
vivax,Brazil (SRS693934)
vivax,Colombia (SRS693267)
vivax,Colombia (SRS693897)
vivax,Colombia (SRS693950)
vivax,Colombia (SRS694231)
vivax,Colombia (SRS694247)
vivax,El Salvador (SRS365051)
vivax,Mexico (SRS693273)
vivax,Mexico (SRS694229)
vivax,Mexico (SRS694244)
vivax,Mexico (SRS694245)
vivax,Mexico (SRS694267)
vivax,Nicaragua (SRS693469)
vivax,Panama (SRS693266)
vivax,Peru (SRS693307)
vivax,Peru (SRS693354)
vivax,Peru (SRS693364)
vivax,Peru (SRS693476)
vivax,Peru (SRS693569)
vivax,Peru (SRS693942)
vivax,Peru (SRS696214)
vivax,Peru (SRS696216)
vivax,Peru (SRS696220)
vivax,Peru (SRS819436)
vivax,Peru (SRS819493)
vivax,India (SRS696222)
vivax,India (SRS805922)
vivax,India (SRS805942)
vivax,Myanmar (SRS693900)
vivax,Myanmar (SRS693902)
vivax,Myanmar (SRS693908)
vivax,Myanmar (SRS694248)
vivax,Myanmar (SRS694255)
vivax,North Korea (SRS258286)
vivax,Papua New Guinea (SRS693346)
vivax,Papua New Guinea (SRS693903)
vivax,Papua New Guinea (SRS694250)
vivax,Papua New Guinea (SRS819716)
vivax,Papua New Guinea (SRS819740)
vivax,Thailand (SRS693269)
vivax,Thailand (SRS693348)
vivax,Thailand (SRS693583)
vivax,Thailand (SRS693956)
vivax,Thailand (SRS693980)
vivax,Viet Nam (SRS693899)
vivax,Madagascar (SRS363192)
vivax,Madagascar (SRS363193)
vivax,Madagascar (SRS417747)
vivax,Mauritania (SRS258091)
