# Supplementary material for: The genome of the zoonotic malaria parasite Plasmodium simium reveals adaptations to host switching
Source: BMC Biol. 2021 Oct 1;19:219. doi: 10.1186/s12915-021-01139-5 (PMC8485552; doi:10.1186/s12915-021-01139-5)
Supplement: Supplementary file 3 — Additional file 3: Figure S17. DBP1 alignment. Complete alignment of DBP1 protein sequences. The presence of the DBL domain (Pfam: PF0311) and the trans-membrane domain is indicated. [file 12915_2021_1139_MOESM3_ESM.pdf]

Figure S17

Complete alignment of DBP1 protein sequences

```
PCYB_063270-t26_1-p1       MKGKNRPLFFLLVLLLSHKVNNVLLERTIETLQECKNECVKGENCYKLSKGHHYIEEDNIERWLQGTNERRSEENIKYKY
PcyM_0628800-t36_1-p1      MKGKNRPLFFLLVLLLSHKVNNVLLERTIETLQECKNECVKGENCYKLSKGHHYIEEDNIERWLQGTNERRSEENIKYKY
PVP01_0623800.1-p1         MKGKNRSLFVLLVLLLSHKVNNVLLERTIETLLECKNEYVKGENGYKLAKGHHCVEEDNLERWLQGTNERRSEENIKYKY
PVX_110810.1-p1            MKGKNRSLFVLLVLLLLHKVNNVLLERTIETLLECKNEYVKGENGYKLAKGHHCVEEDNLERWLQGTNERRSEENIKYKY
Psim2302_000333900.1       MKGKNRSLFVLLVLLLSHKVNNVLLERTIETLLECKNEYVKGENGYKLAKGHHCVEEDNLERWLQGTNERRSEENIKYKY
Psim3636_000376000.1       MKGKNRSLFVLLVLLLSHKVNNVLLERTIETLLECKNEYVKGENGYKLAKGHHCVEEDNLERWLQGTNERRSEENIKYKY
PsimAD002_000401800.1      MKGKNRSLFVLLVLLLSHKVNNVLLERTIETLLECKNEYVKGENGYKLAKGHHCVEEDNLERWLQGTNERRSEENIKYKY
PsimAD005_000131600.1      MKGKNRSLFVLLVLLLSHKVNNVLLERTIETLLECKNEYVKGENGYKLAKGHHCVEEDNLERWLQGTNERRSEENIKYKY
PsimAF28_001016200.1       MKGKNRSLFVLLVLLLSHKVNNVLLERTIETLLECKNEYVKGENGYKLAKGHHCVEEDNLERWLQGTNERRSEENIKYKY
PsimAF33_000351600.1       MKGKNRSLFVLLVLLLSHKVNNVLLERTIETLLECKNEYVKGENGYKLAKGHHCVEEDNLERWLQGTNERRSEENIKYKY
PvivaxAM01_000437300.1     MKGKNRSLFVLLVLLLLHKVNNVLLERTIETLLECKNEYVKGENGYKLAKGHHCVEEDNLERWLQGTNERRSEENIKYKY
PvivaxAM02_000308000.1     MKGKNRSLFVLLVLLLLHKVNNVLLERTIETLLECKNEYVKGENGYKLAKGHHCVEEDNLERWLQGTNERRSEENIKYKY
AAZ81533.1                 MKGKNRSLFVLLVLLLLHKVNNVLLERTIETLLECKNEYVKGENGYKLAKGHHCVEEDNLERWLQGTNERRSEENIKYKY
AAZ81530.1                 MKGKNRSLFVLLVLLLLHKVNNVLLERTIETLLECKNEYVKGENGYKLAKGHHCVEEDNLERWLQGTNERRSEENIKYKY
ABA39301.1                 MKGKNRSLFVLLVLLLLHKVNNVLLERTIETLLECKNEYVKGENGYKLAKGHHCVEEDNLERWLQGTNERRSEENIKYKY
ACB42428.1                 MKGKNRSLFVLLVLLLLHKVNNVLLERTIETLLECKNEYVKGENGYKLAKGHHCVEEDNLERWLQGTNERRSEENIKYKY
ACB42432                   MKGKNRSLFVLLVLLLLHKVNNVLLERTIETLLECKNEYVKGENGYKLAKGHHCVEEDNLERWLQGTNERRSEENIKYKY
PVL_060021400-t42_1-p1     MKGKNRSLFVLLVLLLSHKVNNVLLERTIETLLECKNEYVKGENGYKLAKGHHCVEEDNLERWLQGTNERRSEENVKYKY
ACB42432.1                 MKGKNRSLFVLLVLLLLHKVNNVLLERTIETLLECKNEYVKGENGYKLAKGHHCVEEDNLERWLQGTNERRSEENIKYKY


PCYB_063270-t26_1-p1       GVTELKIKYEQMNGKRTSRILKESIYEAQNFRDNNYR----------EEKDGEHKTDSKTDNGRSANNLVMLDYDTSSSG
PcyM_0628800-t36_1-p1      GVTELKIKYEQMNGKRTSRILKESIYEAQNFRDNNYR----------EEKDGEHKTDSKTDNGRSANNLVMLDYDTSSSG
PVP01_0623800.1-p1         GVTELKIKYAQMNGKRSSRILKESIYGAHNFGGNSYMEGKDGGDKTGEEKDGEHKTDSKTDNGKGANNLVMLDYETSSNG
PVX_110810.1-p1            GVTELKIKYAQMNGKRSSRILKESIYGAHNFGGNSYMEGKDGGDKTGEEKDGEHKTDSKTDNGKGANNLVMLDYETSSNG
Psim2302_000333900.1       GVTELKIKYAQMNGKRSSRILKESIYGAHNFGGNSYMEGKDGGDKTGEEKDGEHKTDSKTDNGKGANNLVMLDYETSSNG
Psim3636_000376000.1       GVTELKIKYAQMNGKRSSRILKESIYGAHNFGGNSYMEGKDGGDKTGEEKDGEHKTDSKTDNGKGANNLVMLDYETSSNG
PsimAD002_000401800.1      GVTELKIKYAQMNGKRSSRILKESIYGAHNFGGNSYMEGKDGGDKTGEEKDGEHKTDSKTDNGKGANNLVMLDYETSSNG
PsimAD005_000131600.1      GVTELKIKYAQMNGKRSSRILKESIYGAHNFGGNSYMEGKDGGDKTGEEKDGEHKTDSKTDNGKGANNLVMLDYETSSNG
PsimAF28_001016200.1       GVTELKIKYAQMNGKRSSRILKESIYGAHNFGGNSYMEGKDGGDKTGEEKDGEHKTDSKTDNGKGANNLVMLDYETSSNG
PsimAF33_000351600.1       GVTELKIKYAQMNGKRSSRILKESIYGAHNFGGNSYMEGKDGGDKTGEEKDGEHKTDSKTDNGKGANNLVMLDYETSSNG
PvivaxAM01_000437300.1     GVTELKIKYAQMNGKRSSRILKESIYGAHNFGGNSYMEGKDGGDKTGEEKDGEHKTDSKTDNGKGANNLVMLDYETSSNG
PvivaxAM02_000308000.1     GVTELKIKYAQMNGKRSSRILKESIYGAHNFGGNSYMEGKDGGDKTGEEKDGEHKTDSKTDNGKGANNLVMLDYETSSNG
AAZ81533.1                 GVTELKIKYAQMNGKRSSRILKESIYGAHNFGGNSYMEGKDGGDKTGEEKDGEHKTDSKTDNGKGANNLVMLDYETSSNG
AAZ81530.1                 GVTELKIKYAQMNGKRSSRILKESIYGAHNFGGNSYMEGKDGGDKTGEEKDGEHKTDSKTDNGKGENNLVMLDYETSSNG
ABA39301.1                 GVTELKIKYAQMNGKRSSRILKESIYGAHNFGGNSYMEGKDGGDKTGEEKDGEHKTDSKTDNGKGANNLVMLDYETSSNG
ACB42428.1                 GVTELKIKYAQMNGKRSSRILKESIYGAHNFGGNSYMEGKDGGDKTGEEKDGEHKTDSKTDNGKGANNLVMLDYETSSNG
ACB42432                   GVTELKIKYAQMNGKRSSRILKESIYGAHNFGGNSYMEGKDGGDKTGEEKDGEHKTDSKTDNGKGANNLVMLDYETSSNG
PVL_060021400-t42_1-p1     DVTELKIKYAQINGKRSSRILKESIYGAHNFGGNSYMEGKDGGDKIGEEKNGEHKTDSETDNGKGANNLVMLDYGTSSNG
ACB42432.1                 GVTELKIKYAQMNGKRSSRILKESIYGAHNFGGNSYMEGKDGGDKTGEEKDGEHKTDSKTDNGKGANNLVMLDYETSSNG


PCYB_063270-t26_1-p1       HPAVTLDNVLEFVTDHGENSLENSSNGGNLYDIVHKKTMSSGVINHAFLQNSEIVTCNDKRKRQVRDWDCSTKKDVCIPD
PcyM_0628800-t36_1-p1      HPAVTLDNVLEFVTDHGENSLENSSNGGNLYDIVHKKTMSSGVINHAFLQNSEIVTCNDKRKRQVRDWDCSTKKDVCIPD
PVP01_0623800.1-p1         QPAGTLDNVLEFVTGHEGNSRKNSSNGGNPYDIDHKKTISSAIINHAFLQNTVMKNCNYKRKRRERDWDCNTKKDVCIPD
PVX_110810.1-p1            QPAGTLDNVLEFVTGHEGNSRKNSSNGGNPYDIDHKKTISSAIINHAFLQNTVMKNCNYKRKRRERDWDCNTKKDVCIPD
Psim2302_000333900.1       QPAGTLDNVLEFVTGHEGNSRKNSSNGGNPYDIDHKKTISSAIINHAFLQNTVMNNCNYKRKRRERDWDCNTKKDVCIPD
Psim3636_000376000.1       QPAGTLDNVLEFVTGHEGNSRKNSSNGGNPYDIDHKKTISSAIINHAFLQNTVMNNCNYKRKRRERDWDCNTKKDVCIPD
PsimAD002_000401800.1      QPAGTLDNVLEFVTGHEGNSRKNSSNGGNPYDIDHKKTISSAIINHAFLQNTVMNNCNYKRKRRERDWDCNTKKDVCIPD
PsimAD005_000131600.1      QPAGTLDNVLEFVTGHEGNSRKNSSNGGNPYDIDHKKTISSAIINHAFLQNTVMKNCNYKRKRRERDWDCNTKKDVCIPD
PsimAF28_001016200.1       QPAGTLDNVLEFVTGHEGNSRKNSSNGGNPYDIDHKKTISSAIINHAFLQNTVMNNCNYKRKRRERDWDCNTKKDVCIPD
PsimAF33_000351600.1       QPAGTLDNVLEFVTGHEGNSRKNSSNGGNPYDIDHKKTISSAIINHAFLQNTVMNNCNYKRKRRERDWDCNTKKDVCIPD
PvivaxAM01_000437300.1     QPAGTLDNVLEFVTGHEGNSRKNSSNGGNPYDIDHKKTISSAIINHAFLQNTVMKNCNYKRKRRERDWDCNTKKDVCIPD
PvivaxAM02_000308000.1     EPAGTLDNVLEFVTGHEGNSRKNSSNGGNPYDIDHKKTISSAIINHAFLQNTVMKNCNYKRKRRERDWDCNTKKDVCIPD
AAZ81533.1                 QPAGTLDNVLEFVTGHEGNSRKNSSNGGNPYDIDHKKTISSAIINHAFLQNTVMKNCNYKRKRRERDWDCNTKKDVCIPD
AAZ81530.1                 QPAGTLDNVLEFVTEHEGNSRKNSSNGGNPYDIDHKKTISSAIINHAFLQNTVMKNCNYKRKRRERDWDCNTKKDVCIPD
ABA39301.1                 QPAGTLDNVLEFVTGHEGNSRKNSSNGGNPYDIDHKKTISSAIINHAFLQNTVMKNCNYKRKRRERDWDCNTKKDVCIPD
ACB42428.1                 QPAGTLDNVLEFVTGHEGNSRKNSSNGGNPYDIDHKKTISSAIINHAFLQNTVMKNCNYKRKRRERDWDCNTKKDVCIPD
ACB42432                   QPAGTLDNVLEFVTGHEGNSRKNSSNGGNPYDIDHKKTISSAIINHAFLQNTVMKNCNHKRKRRERDWDCNTKKDVCIPD
PVL_060021400-t42_1-p1     QPTGTLDNVLEFVTGHGGNSRENSSNGGNPYDIDHKKTISSAIINHAFLQNSVMKNCNDKRKRRERDWDCNTKKDVCIPD
ACB42432.1                 QPAGTLDNVLEFVTGHEGNSRKNSSNGGNPYDIDHKKTISSAIINHAFLQNTVMKNCNHKRKRRERDWDCNTKKDVCIPD
```

Figure S17 (cont.)

```
PCYB_063270-t26_1-p1        RRYQLCMKEVTNLVNNTDTHFHGDITFRKLDFKRKLMYDAAIEGELLYKKNNYKYNKDLCRDIRWSLGDFGDIIMGTDME
PcyM_0628800-t36_1-p1       RRYQLCMKEVTNLVNNTDTHFHGDITFRKLDFKRKLMYDAAIEGELLYKKNNYKYNKDLCRDIRWSLGDFGDIIMGTDME
PVP01_0623800.1-p1          RRYQLCMKELTNLVNNTDTNFHSDITFRKLYLKRKLIYDAAVEGDLLLKLNNYRYNKDFCKDIRWSLGDFGDIIMGTDME
PVX_110810.1-p1             RRYQLCMKELTNLVNNTDTNFHRDITFRKLYLKRKLIYDAAVEGDLLLKLNNYRYNKDFCKDIRWSLGDFGDIIMGTDME
Psim2302_000333900.1        RRYQLCMKELTNLVNNTDTYFHSDITFRKLYLKRKLMYDAAVEGDLLFKLNNYRYNKDFCKDIRWSLGDFGDIIMGTDME
Psim3636_000376000.1        RRYQLCMKELTNLVNNTDTYFHSDITFRKLYLKRKLMYDAAVEGDLLFKLNNYRYNKDFCKDIRWSLGDFGDIIMGTDME
PsimAD002_000401800.1       RRYQLCMKELTNLVNNTDTYFHSDITFRKLYLKRKLIYDAAVEGDLLFKLNNYRYNKDFCKDIRWSLGDFGDIIMGTDME
PsimAD005_000131600.1       RRYQLCMKELTNLVNNTDTYFHSDITFRKLYLKRKLIYDAAVEGDLLFKLNNYRYNKDFCKDIRWSLGDFGDIIMGTDME
PsimAF28_001016200.1        RRYQLCMKELTNLVNNTDTYFHSDITFRKLYLKRKLIYDAAVEGDLLFKLNNYRYNKDFCKDIRWSLGDFGDIIMGTDME
PsimAF33_000351600.1        RRYQLCMKELTNLVNNTDTYFHSDITFRKLYLKRKLIYDAAVEGDLLFKLNNYRYNKDFCKDIRWSLGDFGDIIMGTDME
PvivaxAM01_000437300.1      RRYQLCMKELTNLVNNTDTNFHRDITFRKLYLKRKLIYDAAVEGDLLFKLNNYRYNKDFCKDIRWSLGDFGDIIMGTDME
PvivaxAM02_000308000.1      RRYQLCMKELTNLVNNTDTNFHRDITFRKLYLKRKLIYDAAVEGDLLLKLNNYRYNKDFCKDIRWSLGDFGDIIMGTDME
AAZ81533.1                  RRYQLCMKELTNLVNNTDTNFHSDITFRKLYLKRKLIYDAAVEGDLLLKLNNYRYNKDFCKDIRWSLGDFGDIIMGTDME
AAZ81530.1                  RRYQLCMKELTNLVNNTDTNFHSDITFRKLYLKRKLIYDAAVEGDLLLKLNNYRYNKDFCKDIRWSLGDFGDIIMGTDME
ABA39301.1                  RRYQLCMKELTNLVNNTDTNFHRDITFRKLYLKRKLIYDAAVEGDLLLKLNNYRYNKDFCKDIRWSLGDFGDIIMGTDME
ACB42428.1                  RRYQLCMKELTNLVNNTDTNLHSDITFRKLYLKRKLIYDAAVEGDLLLKLNNYRYNKDFCKDIRWSLGDFGDIIMGTDME
ACB42432                    RRYQLCMKELTNLVNNTDTYFHSDITFRKLYLKRKLIYDAAVEGDLLFKLNNYRYNKDFCKDIRWSLGDFGDIIMGTDME
PVL_060021400-t42_1-p1      RRYQLCIKELTNLVNNTDTNFHSDITFRKLYLKRKLIYDAAVEGDLLLKLNNYRYNKDFCKDIRWSLGDFGDIIMGTDME
ACB42432.1                  RRYQLCMKELTNLVNNTDTYFHSDITFRKLYLKRKLIYDAAVEGDLLFKLNNYRYNKDFCKDIRWSLGDFGDIIMGTDME


PCYB_063270-t26_1-p1        GIGHSQVVENNLKSIFGTGKNALQNRVQWWHESKVHIWRAMMYSVRKRLKDRFAWICKINVAVNVEPQIYRWIREWGGDY
PcyM_0628800-t36_1-p1       GIGHSQVVENNLKSIFGTGKNALQNRVQWWHESKVHIWRAMMYSVRKRLKDRFAWICKINVAVNVEPQIYRWIREWGGDY
PVP01_0623800.1-p1          GIGYSKVVENNLRSIFGTGEKAQQHRKQWWNESKAQIWTAMMYSVKKRLKGNFIWICKINVAVNIEPQIYRWIREWGRDY
PVX_110810.1-p1             GIGYSKVVENNLRSIFGTDEKAQQRRKQWWNESKAQIWTAMMYSVKKRLKGNFIWICKLNVAVNIEPQIYRWIREWGRDY
Psim2302_000333900.1        GIGYSNVVENNLRSIFGTGQNAQQHRKQWWNESKAQIWRAMMYSVNKRLKGNFRWICKINVAVNIEPQIYRWIREWGRDY
Psim3636_000376000.1        GIGYSNVVENNLRSIFGTGQNAQQHRKQWWNESKAQIWRAMMYSVNKRLKGNFRWICKINVAVNIEPQIYRWIREWGRDY
PsimAD002_000401800.1       GIGYSKVVENNLRSIFGTGKTAQQHRKQWWNESKAQIWRAMMYSVNKRLKGNFIWICKINVAVNIEPQIYRWIREWGRDY
PsimAD005_000131600.1       GIGYSKVVENNLRSIFGTGKNAQQHRKQWWNESKAQIWRAMMYSVNKRLKGNFLWICKINVAVNIEPQIYRWIREWGRDY
PsimAF28_001016200.1        GIGYSKVVENNLRSIFGTGKTAQQHRKQWWNESKAQIWRAMMYSVNKRLKGNFIWICKINVAVNIEPQIYRWIREWGRDY
PsimAF33_000351600.1        GIGYSKVVENNLRSIFGTGQNAQQHRKQWWNESKAQIWRAMMYSVNKRLKGNFIWICKINVAVNIEPQIYRWIREWGRDY
PvivaxAM01_000437300.1      GIGYSKVVEDNLRSIFGTGKNAQQHRKQWWNESKAQIWTAMMYSVKKRLKGKFIWICKINVAVNIEPQIYRRIREWGRDY
PvivaxAM02_000308000.1      GIGYSEVVENNLRSIFGTGEQAQQRRKQWWNESKAQIWTAMMYSVKKRLKGKFIWICKINVAVNIEPQIYRRIREWGRDY
AAZ81533.1                  GIGYSKVVENNLRSIFGTGKNAQQHRKQWWNETKAQIWRAMMYSVKKRLKGNFIWICKINVAVNIEPQIYRWIREWGRDY
AAZ81530.1                  GIGYSEVVENNLRSIFGTGKNAQQRRKQWWNESKAQIWTAMMYSVKKRLKGKFIWICKINVAVNIEPQIYRRIREWGRDY
ABA39301.1                  GIGYSKVVENNLRSIFGTGEKAQQRRKQWWNESKAQIWTAMMYSVKKRLKGKFMWICKINVAVNIEPQIYRRIREWGRDY
ACB42428.1                  GIGYSKVVENNLRSIFGTGKNAQQHRKQWWNETKAQIWRAMMYSVKKRLKGNFIWICKINVAVNIEPQIYRWIREWGRDY
ACB42432                    GIGYSKVVENNLRSIFGTGKNAQQHRKQWWNESKAQIWRAMMYSVNKRLKGNFIWICKINVAVNIEPQIYRWIREWGRDY
PVL_060021400-t42_1-p1      GIGYSKVVENNLRSIFGTGEKAQQHRKQWWNESKAHIWRAMMYSVKKRLKGDFKWICKINVAVNIEPQIYRWIREWGRDY
ACB42432.1                  GIGYSKVVENNLRSIFGTGKNAQQHRKQWWNESKAQIWRAMMYSVNKRLKGNFIWICKINVAVNIEPQIYRWIREWGRDY


PCYB_063270-t26_1-p1        ITELPTERQKVKEKCDGNIIFTKKKVCTV-SQCKNACNLYDQWITRKKEQWDVLSNKFKSVKNEQSIKTADIATAYDILK
PcyM_0628800-t36_1-p1       ITELPTERQKVKEKCDGNIIFTKKKVCTV-SQCKNACNLYDQWITRKKEQWDVLSNKFKSVKNEQSIKTADIATAYDILK
PVP01_0623800.1-p1          VKELPTEVQKLKEKCDGKINYTDKKVCKV-PPCQNACKSYDQWITRKKNQWDVLSNKFISVKNAEKVQTAGIVTPYDILK
PVX_110810.1-p1             VSELPTEVQKLKEKCDGKINYTDKKVCKV-PPCQNACKSYDQWITRKKNQWDVLSNKFISVKNAEKVQTAGIVTPYDILK
Psim2302_000333900.1        VSELPTEVQKLKEKCDGKINYTDKKVCKV-PPCQNACKSYDQWITRKKNQWDVLSNKFKSVKNAEKVQTAGIVTPYDILK
Psim3636_000376000.1        VSELPTEVQKLKEKCDGKINYTDKKVCKV-PPCQNACKSYDQWITRKKNQWDVLSNKFKSVKNAEKVQTAGIVTPYDILK
PsimAD002_000401800.1       VSELPTEVQKLKEKCDGKINYTDKKVCKV-PPCQNACKSYDQWITRKKNQWDVLSNKFISVKNAEKVQTAGIVTPYDILK
PsimAD005_000131600.1       VSELPTEVQKLKEKCDGKINYTDKKVCKV-PPCQNACKSYDQWITRKKNQWDVLSNKFISVKNAEKVQTAGIVTPYDILK
PsimAF28_001016200.1        VSELPTEVQKLKEKCDGKINYTDKKVCKV-PPCQNACKSYDQWITRKKNQWDVLSNKFISVKNAEKVQTAGIVTPYDILK
PsimAF33_000351600.1        VSELPTEVQKLKEKCDGKINYTDKKVCKV-PPCQNACKSYDQWITRKKNQWDVLSNKFKSVKNAEKVQTAGIVTPYDILK
PvivaxAM01_000437300.1      VSELPTEVQKLKEKCDGKINYTDKKVCKV-PPCQNACKSYDQWITRKKNQWDVLSNKFKSVKNAEKVQTAGIVTPYDILK
PvivaxAM02_000308000.1      VSELPTEVQKLKEKCDGKINYTDKKVCKV-PPCQNACKSYDQWITRKKNQWDVLSNKFKSVKNAEKVQTAGIVTPYDILK
AAZ81533.1                  VSELPTEVQKLKEKCDGKINYTDKKVCKVLPPCQNACKSYDQWITRKKNQWDVLSNKFISVKNAEKVQTAGIVTPYDILK
AAZ81530.1                  VSELPTEVQKLKEKCDGKINYTDKKVCKV-PPCQNACKSYDQWITRKKNQWDVLSNKFKSVKNAEKVQTAGIVTPYDILK
ABA39301.1                  VSELPTEVQKLKEKCDGKINYTDKKVCKV-PPRQDACKSYDQWITRKKNQWDVLSNKFISVKNAEKVQTAGIVTPYDILK
ACB42428.1                  VSELPTEVQKLKEKCDGKINYTDKKVCKVLPPCQNACKSYDQWITRKKNQWDVLSNKFISVKNAEKVQTAGIVTPYDILK
ACB42432                    VSELPTEVQKLKEKCDGKINYTDKKVCKV-PPCQNACKSYDQWITRKKNQWDVLSNKFKSVKNAEKVQTAGIVTPYDILK
PVL_060021400-t42_1-p1      VSELPTEVQKLKEKCDGKINYTDKKVCTV-PPCQDACKSYDQWITRKKNQWDVLSNKFISVKSAEKVQTAGIVTPYDILK
ACB42432.1                  VSELPTEVQKLKEKCDGKINYTDKKVCKV-PPCQNACKSYDQWITRKKNQWDVLSNKFKSVKNAEKVQTAGIVTPYDILK
DBL domain                  |<------------------------------------------------------------------------------
```

Figure S17 (cont.)

```
PCYB_063270-t26_1-p1        QEINKFNEETFENEINKLDNAYIDLCLCSLEEVKKHTQNVVRNIEKAAKSVAPNPNPINKAVDSSKAEKVQGNPENGNVN
PcyM_0628800-t36_1-p1       QEINKFNEETFENEINKLDNAYIDLCLCSLEEVKKHTQNVVRNIEKAAKSVAPNPNPINKAVDSSKAEKVQGNPENGNVN
PVP01_0623800.1-p1          QELDEFNEVAFENEINKRDGAYIELCVCSVEEAKKNTQEVVTNVDNAAKSQATNSNPISQPVDSSKAEKVPGDSTHGNVN
PVX_110810.1-p1             QELDEFNEVAFENEINKRDGAYIELCVCSVEEAKKNTQEVVTNVDNAAKSQATNSNPISQPVDSSKAEKVPGDSTHGNVN
Psim2302_000333900.1        QELDEFNEVAFENEINKRDGAYIELCDCSVEEAKKNTQEVVTNVDNAAKSQATNSNPISQPVDSSKAEKVPGDSTHGNVN
Psim3636_000376000.1        QELDEFNEVAFENEINKRDGAYIELCDCSVEEAKKNTQEVVTNVDNAAKSQATNSNPISQPVDSSKAEKVPGDSTHGNVN
PsimAD002_000401800.1       QELDEFNEVAFENEINKRDGAYIELCDCSVEEAKKNTQEVVTNVDNAAKSQATNSNPISQPVDSSKAEKVPGDSTHGNVN
PsimAD005_000131600.1       QELDEFNEVAFENEINKRDGAYIELCDCSVEEAKKNTQEVVTNVDNAAKSQATNSNPISQPVDSSKAEKVPGDSTHGNVN
PsimAF28_001016200.1        QELDEFNEVAFENEINKRDGAYIELCDCSVEEAKKNTQEVVTNVDNAAKSQATNSNPISQPVDSSKAEKVPGDSTHGNVN
PsimAF33_000351600.1        QELDEFNEVAFENEINKRDGAYIELCDCSVEEAKKNTQEVVTNVDNAAKSQATNSNPISQPVDSSKAEKVPGDSTHGNVN
PvivaxAM01_000437300.1      QELDEFNEVAFENEINKRDGAYIELCVCSVEEAKKNTQEVVTNVDNAAKSQATNSNPISQPVDSSKAEKVPGDSTHGNVN
PvivaxAM02_000308000.1      QELDEFNEVAFENEINKRDGAYIELCVCSVEEAKKNTQEVVTNVDNAAKSQATNSNPISQPVDSSKAKKVPGDSTHGNVN
AAZ81533.1                  QELDEFNEMAFENEINKRDGAYIELCVCSVEEAKKNTQEVVTNVDNAAKSQATNSNPISQPVDSSKAEKVPGDSTHGNVN
AAZ81530.1                  QELDEFNEVAFENEINKRDGAYIELCVCSVEEAKKNTQEVVTNVDNAAKSQATNSNPISQPVDSSKAEKVPGDSTHGNVN
ABA39301.1                  QELDEFNEVAFENEINKRDGAHIELCVCSVEEAKKNTQEVVTNVDNAAKSQATNSNPISQPVDSSKAEKVPGDSTHGNVN
ACB42428.1                  QELDEFNEVAFENEINKRDGAYIELCVCSVEEAKKNTQEVVTNVDNAAKSQATNSNPISQPVDSSKAEKVPGDSTHGNVN
ACB42432                    QELDEFNEVAFENEINKRDGAYIELCVCSVEEAKKNTQEVVTNVDNAAKSQATNSNPISQPVDSSKAEKVPGDSTHGNVN
PVL_060021400-t42_1-p1      QELDEFNEVAFENEINKHDDAYIELCVCSVEEAKKNTQEVVTNVENAAKSQAPNSNPISQPVDSSKAEKVPGDSTHGNVN
ACB42432.1                  QELDEFNEVAFENEINKRDGAYIELCVCSVEEAKKNTQEVVTNVDNAAKSQATNSNPISQPVDSSKAEKVPGDSTHGNVN
DBL domain                  ----------------------------------------->|

PCYB_063270-t26_1-p1        SGANNSTTGKAATGDGQNGNQTPTKSNVQQSDVAESAGAKNVDPHKSVSEKSADTTSVTSIAEAGKENLGTSNSQPSKST
PcyM_0628800-t36_1-p1       SGANNSTTGKAATGDGQNGNQTPTKSNVQQSDVAESAGAKNVDPHKSVSEKSADTTSVTSIAEAGKENLGTSNSQPSKST
PVP01_0623800.1-p1          SGQDSSTTGKAVTGDGQNGNQTPAESDVQRSDIAESVSAKNVDPQKSVSERSDDTASVTGIAEAGKENLGASNSRPSEST
PVX_110810.1-p1             SGQDSSTTGKAVTGDGQNGNQTPAESDVQRSDIAESVSAKNVDPQKSVSKRSDDTASVTGIAEAGKENLGASNSRPSEST
Psim2302_000333900.1        SGQDSSTTGKAVTGDGQNGNQTPAESDVQRSDIAESVSAKNVDPQKSVSERSDDTASVTGIAEAGKENLGASNSRPSEST
Psim3636_000376000.1        SGQDSSTTGKAVTGDGQNGNQTPAESDVQRSDIAESVSAKNVDPQKSVSERSDDTASVTGIAEAGKENLGASNSRPSEST
PsimAD002_000401800.1       SGQDSSTTGKAVTGDGQNGNQTPAESDVQRSDIAESVSAKNVDPQKSVSERSDDTASVTGIAEAGKENLGASNSRPSEST
PsimAD005_000131600.1       SGQDSSTTGKAVTGDGQNGNQTPAESDVQRSDIAESVSAKNVDPQKSVSERSDDTASVTGIAEAGKENLGASNSRPSEST
PsimAF28_001016200.1        SGQDSSTTGKAVTGDGQNGNQTPAESDVQRSDIAESVSAKNVDPQKSVSERSDDTASVTGIAEAGKENLGASNSRPSEST
PsimAF33_000351600.1        SGQDSSTTGKAVTGDGQNGNQTPAESDVQRSDIAESVSAKNVDPQKSVSERSDDTASVTGIAEAGKENLGASNSRPSEST
PvivaxAM01_000437300.1      SGQDSSTTGKAVTGDGKNGNQTPAESDVQRSDIAESVSAKNVDPQKSVSERSDDTASVTGIAEAGKENLGASNSRPSEST
PvivaxAM02_000308000.1      SGQDSSTTGKAVTGDGQNGNQTPAESDVQRSDIAESVSAKNVDPQKSVSERSDDTASVTGIAEAGKENLGASNSRPSEST
AAZ81533.1                  SGQDSSTTGKAVTGDGQNGNQTPAESDVQRSDIAESVSAKNVDPQKSVSERSDDTASVTGIAEAGKENLSASNSRPSEST
AAZ81530.1                  SGQDSSTTGKAVTGDGQNGNQTPAESDVQRSDIAESVSAKNVDPQKSVSERSDDTASVTGIAEAGKENLGASNSRPSEST
ABA39301.1                  SGQDSSTTGKAVTGDGKNGNQTPAESDVQRSDIAESVSAKNVDPQKSVSERSDDTASVTGIAEAGKENLGASNSRPSEST
ACB42428.1                  SGQDSSTTGKAVTGDGQNGNQTPAESDVQRSDIAESVSAKNVDPQKSVSERSDDTASVTGIAEAGKENLGASNSRPSEST
ACB42432                    SGQDSSTTGKAVTGDGQNGNQTPAESDVQRSDIAESVSAKNVDPQKSVSKRSDDTASVTGIAEAGKENLGASNSRPSEST
PVL_060021400-t42_1-p1      SGQDSSTTGKAVTGDGQNGNQTPAESDVQRSDIAESVSAKNVDPQKSVSERSDDTASVTGVTESGKENLGASNSPPSEPT
ACB42432.1                  SGQDSSTTGKAVTGDGQNGNQTPAESDVQRSDIAESVSAKNVDPQKSVSKRSDDTASVTGIAEAGKENLGASNSRPSEST


PCYB_063270-t26_1-p1        VEANSPGDGTVNSASISVKNSEKPLVTTDKGLEPSKDNSDNNGSTE------SKKSEANPDSNSKGETGMGQDNDKEKAT
PcyM_0628800-t36_1-p1       VEANSPGDGTVNSASISVKNSEKPLVTTDKGLEPSKDNSDNNGSTE------SKKSEANPDSNSKGETGMGQDNDKEKAT
PVP01_0623800.1-p1          VEANSPGDDTVNSASIPVVSGENPLVTPYNGLGHSKDNSDSDGPAEFAESTKSAESMANPDSNSKGETGKGQDNDMAKAT
PVX_110810.1-p1             VEANSPGDDTVNSASIPVVSGENPLVTPYNGLRHSKDNSDSDGP---------AESMANPDSNSKGETGKGQDNDMAKAT
Psim2302_000333900.1        VEANSPGDDTVNSASIPVVSGKNPLVTPYNGL--------------------------------------GQDNDMAKAT
Psim3636_000376000.1        VEANSPGDDTVNSASIPVVSGKNPLVTPYNGL--------------------------------------GQDNDMAKAT
PsimAD002_000401800.1       VEANSPGDDTVNSASIPVVSGKNPLVTPYNGL--------------------------------------GQDNDMAKAT
PsimAD005_000131600.1       VEANSPGDDTVNSASIPVVSGKNPLVTPYNGL--------------------------------------GQDNDMAKAT
PsimAF28_001016200.1        VEANSPGDDTVNSASIPVVSGKNPLVTPYNGL--------------------------------------GQDNDMAKAT
PsimAF33_000351600.1        VEANSPGDDTVNSASIPVVSGKNPLVTPYNGL--------------------------------------GQDNDMAKAT
PvivaxAM01_000437300.1      VEANSPGDDTVNSASIPVVSGENPLVTPYNGLRHSKDNSDSDGP---------AESMANPDSNSKGETGKGQDNDMAKAT
PvivaxAM02_000308000.1      VEANSPGDDTVNSASIPVVSGENPLVTPYNGLRHSKDNSDSDGP---------AESMANPDSNSKGETGKGQDNDMAKAT
AAZ81533.1                  VEANSPGDDTVNSASIPVVSGENPLVTPYNGLRHSKDNSDSDGP---------AESMANPDSNSKGETGKGQDNDMAKAT
AAZ81530.1                  VEANSPGDDTVNSASIPVVSGENPLVTPYNGLRHSKDNSDSDGP---------AESMANPDSNSKGETGKGQDNDMAKAT
ABA39301.1                  VEANSPGDDTVNSASIPVVSGENPLVTPYNGLRHSKDNSDSDGP---------AESMANPDSNSKGETGKGQDNDMAKAT
ACB42428.1                  VEANSPGDDTVNSASIPVVSGENPLVTPYNGLRHSKDNSDSDGP---------AESMANPDSNSKGETGKGQDNDMAKAT
ACB42432                    VEANSPGDDTVNSASIPVVSGENPLVTPYNGLRHSKDNSDSDGP---------AESMANPDSNSKGETGKGQDNDMAKAT
PVL_060021400-t42_1-p1      VEANSPGDGTVNSASIPVVSGENPLVTPDNGLGHSKDNSDSDGPAEFAESMKSVESMANPDSNSKGETGKGQDNDMAKAT
ACB42432.1                  VEANSPGDDTVNSASIPVVSGENPLVTPYNGLRHSKDNSDSDGP---------AESMANPDSNSKGETGKGQDNDMAKAT
```

Figure S17 (cont.)

```
PCYB_063270-t26_1-p1      KDSSNSSDNTSSAKGDTTSAVDRNFNGGVPEDRDKIVGSKKEEKEDNSANKDAATVV----------GGNTNDRTENGME
PcyM_0628800-t36_1-p1     KDSSNSSDNTSSAKGDTTSAVDRNFNGGVPEDRDKIVGSKKEEKEDNSANKDAATVV----------GGNTNDRTENGME
PVP01_0623800.1-p1        KDSSNSSDGTSSATGDTTDAVDREINKGVPEDRDKTVGSKDGGGEDNSANKDAATVVGEDRIRENSAGGSTNDRSKNDTE
PVX_110810.1-p1           KDSSNSSDGTSSATGDTTDAVDREINKGVPEDRDKTVGSKDGGGEDNSANKDAATVVGEDRIRENSAGGSTNDRSKNDTE
Psim2302_000333900.1      KDSSNSSDGTSSATGDTTDAVDREINKGVPEDRDKTVGSKDGGGEDNSANKDAATVVGEDRIRENSAGGSTNDRSKNDTE
Psim3636_000376000.1      KDSSNSSDGTSSATGDTTDAVDREINKGVPEDRDKTVGSKDGGGEDNSANKDAATVVGEDRIRENSAGGSTNDRSKNDTE
PsimAD002_000401800.1     KDSSNSSDGTSSATGDTTDAVDREINKGVPEDRDKTVGSKDGGGEDNSANKDAATVVGEDRIRENSAGGSTNDRSKNDTE
PsimAD005_000131600.1     KDSSNSSDGTSSATGDTTDAVDREINKGVPEDRDKTVGSKDGGGEDNSANKDAATVVGEDRIRENSAGGSTNDRSKNDTE
PsimAF28_001016200.1      KDSSNSSDGTSSATGDTTDAVDREINKGVPEDRDKTVGSKDGGGEDNSANKDAATVVGEDRIRENSAGGSTNDRSKNDTE
PsimAF33_000351600.1      KDSSNSSDGTSSATGDTTDAVDREINKGVPEDRDKTVGSKDGGGEDNSANKDAATVVGEDRIRENSAGGSTNDRSKNDTE
PvivaxAM01_000437300.1    KDSSNSSDGTSSATGDTTDAVDREINKGVPEDRDKTVGSKDGGGEDNSANKDAATVVGEDRIRENSAGGSTNDRSKNDTE
PvivaxAM02_000308000.1    KDSSNSSDGTSSARGDTTDAVDREINKGVPEDRDKTVGSKDGGGEDNSANKDAATVVGEDRIRENSAGGSTNDRSKNDTE
AAZ81533.1                KDSSNSSDGTSSATGDTTDAVDREINKGVPEDRDKTVGSKDGGGEDNSANKDAATVVGEDRIRENSAGGSTNDRSKNDTE
AAZ81530.1                KDSSNSSDGTSSATGDTTDAVDREINKGVPEDRDKTVGSKDGGGEDNSANKDAATVVGEDRIRENSAGGSTNDRSKNDTE
ABA39301.1                KDSSNSSDGTSSATGDTTDAVDREINKGVPEDRDKTVGSKDGGGEDNSANKDAATVVGEDRIRENSAGGSTNDRSKNDTE
ACB42428.1                KDSSNSSDGTSSATGDTTDAVDREINKGVPEDRDKTVGSKDGGGEDNSANKDAATVVGEDRIRENSAGGSTNDRSKNDTE
ACB42432                  KDSSNSSDGTSSATGDTTDAVDREINKGVPEDRDKTVGSKDGGGEDNSANKDAATVVGEDRIRENSAGGSTNDRSKNDTE
PVL_060021400-t42_1-p1    KDSSNSSDSTNSATGDATGAVDREINKGVPEDSDKTVGSKEGGGEDNSANKDAAVVVGEDRIRENSAGGSINDRSKNDTE
ACB42432.1                KDSSNSSDGTSSATGDTTDAVDREINKGVPEDRDKTVGSKDGGGEDNSANKDAATVVGEDRIRENSAGGSTNDRSKNDTE

PCYB_063270-t26_1-p1      KNNVPAPDSKQSVDATPLSKTESLELNESAHRITNDTTHSLKNENEGSEKDLQKHDFTNNDMPNEEPNSAQTTDAEGHHR
PcyM_0628800-t36_1-p1     KNNVPAPDSKQSVDATPLSKTESLELNESAHRITNDTTHSLKNENEGSEKDLQKHDFTNNDMPNEEPNSAQTTDAEGHHR
PVP01_0623800.1-p1        KNGASTPDSKQSEDATALSKTESLESTESGDRTTNDTTNSLENKNGGKEKDLQKHDFKSNDTPNEEPNSDQTTDVEGHDR
PVX_110810.1-p1           KNGASTPDSKQSEDATALSKTESLESTESGDRTTNDTTNSLENKNGGKEKDLQKHDFKSNDTPNEEPNSDQTTDAEGHDR
Psim2302_000333900.1      KNGASTPDSKQSEDATALSKTESLESTESGDRTTNDTTNSLENKNGGKEKDLQKHDFKSNDTPNEEPNSDQTTDAEGHDR
Psim3636_000376000.1      KNGASTPDSKQSEDATALSKTESLESTESGDRTTNDTTNSLENKNGGKEKDLQKHDFKSNDTPNEEPNSDQTTDAEGHDR
PsimAD002_000401800.1     KNGASTPDSKQSEDATALSKTESLESTESGDRTTNDTTNSLENKNGGKEKDLQKHDFKSNDTPNEEPNSDQTTDAEGHDR
PsimAD005_000131600.1     KNGASTPDSKQSEDATALSKTESLESTESGDRTTNDTTNSLENKNGGKEKDLQKHDFKSNDTPNEEPNSDQTTDAEGHDR
PsimAF28_001016200.1      KNGASTPDSKQSEDATALSKTESLESTESGDRTTNDTTNSLENKNGGKEKDLQKHDFKSNDTPNEEPNSDQTTDAEGHDR
PsimAF33_000351600.1      KNGASTPDSKQSEDATALSKTESLESTESGDRTTNDTTNSLENKNGGKEKDLQKHDFKSNDTPNEEPNSDQTTDAEGHDR
PvivaxAM01_000437300.1    KNGASTPDSKQSEDATALSKTESLESTESGDRTTNDTTNSLENKNGGKEKDLQKHDFKSNDTPNEEPNSDQTTDAEGHDR
PvivaxAM02_000308000.1    KNGASTPDSKQSEDATALSKTESLESTESGDRTTNDTTNSLENKNGGKEKDLQKHDFKSNDTPNEEPNSDQTTDAEGHDR
AAZ81533.1                KNGASTPDSKQSEDATALSKTESLESTESGDRTTNDTTISLENKNGGKEKDLQKHDFKSNDTPNEEPNSDQTTDAEGHDR
AAZ81530.1                KNGASTPDSKQSEDATALSKTESLESTESGDRTTNDTTNSLENKNGGKEKDLQKHDFKSNDTPNEEPNSDQTTDAEGHDR
ABA39301.1                KNGASTPDSKQSEDATALSKTESLESTESGDRTTNDTTNSLENKNGGKEKDLQKHDFKSNDTPNEEPNSDQTTDAEGHDR
ACB42428.1                KNGASTPDSKQSEDATALSKTESLESTESGDRTTNDTTNSLENKNGGKEKDLQKHDFKSNDTPNEEPNSDQTTDVEGHDR
ACB42432                  KNGASTPDSKQSEDATALSKTESLESTESGDRTTNDTTNSLENKNGGKEKDLQKHDFKSNDTPNEEPNSDQTTDAEGHDR
PVL_060021400-t42_1-p1    KNGAPTPDSKQSEDATALSKTESLESTESVDRTTTDTTNSLENKNGGKEKDLQKHDFKSNDTPNEEPNSDQTTDAEGPDR
ACB42432.1                KNGASTPDSKQSEDATALSKTESLESTESGDRTTNDTTNSLENKNGGKEKDLQKHDFKSNDTPNEEPNSDQTTDAEGHDR

PCYB_063270-t26_1-p1      YSMKNDNGEMRKHMNKGTFTKNPNSNQLNSHNDLSNGKLDIKEYKYRDVNATREKIIYMSEVRRCNNNISLNYCNSVKDK
PcyM_0628800-t36_1-p1     YSMKNDNGEMRKHMNKGTFTKNPNSNQLNSHNDLSNGKLDIKEYKYRDVNATREKIIYMSEVRRCNNNISLNYCNSVKDK
PVP01_0623800.1-p1        DSIKNDKAERRKHMNKDTFTKNTNSHHLNSNNNLSNGKLDIKEYKYRDVKATRENIILMSSVRKCNNNISLEYCNSVEDK
PVX_110810.1-p1           DSIKNDKAERRKHMNKDTFTKNTNSHHLNSNNNLSNGKLDIKEYKYRDVKATREDIILMSSVRKCNNNISLEYCNSVEDK
Psim2302_000333900.1      DSIKNDKAERRKHMNKDTFTKNTNSHHLNSNNNLSNGKLDIKEYKYRDVKATRENIILMSSVRKCNNNISLKYCNSVEDK
Psim3636_000376000.1      DSIKNDKAERRKHMNKDTFTKNTNSHHLNSNNNLSNGKLDIKEYKYRDVKATRENIILMSSVRKCNNNISLKYCNSVEDK
PsimAD002_000401800.1     DSIKNDKAERRKHMNKDTFTKNTNSHHLNSNNNLSNGKLDIKEYKYRDVKATRENIILMSSVRKCNNNISLKYCNSVEDK
PsimAD005_000131600.1     DSIKNDKAERRKHMNKDTFTKNTNSHHLNSNNNLSNGKLDIKEYKYRDVKATRENIILMSSVRKCNNNISLEYCNSVEDK
PsimAF28_001016200.1      DSIKNDKAERRKHMNKDTFTKNTNSHHLNSNNNLSNGKLDIKEYKYRDVKATRENIILMSSVRKCNNNISLKYCNSVEDK
PsimAF33_000351600.1      DSIKNDKAERRKHMNKDTFTKNTNSHHLNSNNNLSNGKLDIKEYKYRDVKATRENIILMSSVRKCNNNISLKYCNSVEDK
PvivaxAM01_000437300.1    DSIKNDKAERRKHMNKDTFTKNTNSHHLNSNNNLSNGKLDIKEYKYRDVKATRENIILMSSVRKCNNNISLEYCNSVEDK
PvivaxAM02_000308000.1    DSIKNDKAERRKHMNKDTFTKNTNSHHLNSNNNLSNGKLDIKEYKYRDVKATRENIILMSSVRKCNNNISLEYCNSVEDK
AAZ81533.1                DSIKNDKAERRKHMNKDTFTKNTNSHHLNSNNNLSNGKLDIKEYKYRDVKATRENIILMSSVRKCSNNISLEYCNSVEDK
AAZ81530.1                DSIKNDKAERRKHMNKDTFTKNTNSHHLNSNNNLSNGKLDIKEYKYRDVKATREDIILMSSVRKCNNNISLEYCNSVEDK
ABA39301.1                DSIKNDKAERRKHMNKDTFTKNTNSHHLNSNNNLSNGKLDIKEYKYRDVKATREDIILMSSVRKCNNNISLEYCNSVEDK
ACB42428.1                DSIKNDKAERRKHMNKDTFTKNTNSHHLNSNNNLSNGKLDIKEYKYRDVKATRENIILMSSVRKCNNNISLEYCNSVEDK
ACB42432                  DSIKNDKAERRKHMNKDTFTKNTNSHHLNSNNNLSNGKLDIKEYKYRDVKATREDIILMSSVRKCNNNISLEYCNSVEDK
PVL_060021400-t42_1-p1    DGIKNDKAERRKHMNKDTFTKNQNSHHLNSNNNLGNGKLDIKEYEYRDVNATREKIILMSAVRKCNNNISLKYCNSVEDK
ACB42432.1                DSIKNDKAERRKHMNKDTFTKNTNSHHLNSNNNLSNGKLDIKEYKYRDVKATREDIILMSSVRKCNNNISLEYCNSVEDK
```

Figure S17 (cont.)

```
PCYB_063270-t26_1-p1      MSSNTCSREKSKNLCCSISDYCLNYFEVYTNEYHNCMKKEFEDPSYKCFTKGGFTDKAYFAAGGALLILLLLITSRHMIK
PcyM_0628800-t36_1-p1     MSSNTCSREKSKNLCCSISDYCLNYFEVYTNEYHNCMKKEFEDPSYKCFTKGGFTDKAYFAAGGALLILLLLITSRHMIK
PVP01_0623800.1-p1        ISSNTCSREKSKNLCCSISDFCLNYFDVYSYEYHSCMKKEFEDPSYKCFTKGGFKDKTYFAAAGALLILLLLIASRKMIK
PVX_110810.1-p1           ISSNTCSREKSKNLCCSISDFCLNYFDVYSYEYLSCMKKEFEDPSYKCFTKGGFKDKTYFAAAGALLILLLLIASRKMIK
Psim2302_000333900.1      ISSNTCSREKSKNLCCSISDFCLNYFDVHSYEYLSCMKKEFEDPSYKCFTKGGFKDKAYFAAAGALLILLLLIASRKMIK
Psim3636_000376000.1      ISSNTCSREKSKNLCCSISDFCLNYFDVHSYEYLSCMKKEFEDPSYKCFTKGGFKDKAYFAAAGALLILLLLIASRKMIK
PsimAD002_000401800.1     ISSNTCSREKSKNLCCSISDFCLNYFDVHSYEYLSCMKKEFEDPSYKCFTKGGFKDKAYFAAAGALLILLLLIASRKMIK
PsimAD005_000131600.1     ISSNTCSREKSKNLCCSISDFCLNYFDVHSYEYLSCMKKEFEDPSYKCFTKGGFKDKTYFAAAGALLILLLLIASRKMIK
PsimAF28_001016200.1      ISSNTCSREKSKNLCCSISDFCLNYFDVHSYEYLSCMKKEFEDPSYKCFTKGGFKDKAYFAAAGALLILLLLIASRKMIK
PsimAF33_000351600.1      ISSNTCSREKSKNLCCSISDFCLNYFDVHSYEYLSCMKKEFEDPSYKCFTKGGFKDKAYFAAAGALLILLLLIASRKMIK
PvivaxAM01_000437300.1    ISSNTCSREKSKNLCCSISDFCLNYFDVNSYEYHSCMKKEFEDPSYKCFTKGGFKDKTYFAAAGALLILLLLIASRKMIK
PvivaxAM02_000308000.1    ISSNTCSREKSKNLCCSISDFCLNYFDVYSYEYLSCMKKEFEDPSYKCFTKGGFKDKTYFAAAGALLILLLLIASRKMIK
AAZ81533.1                ISSNTCSREKSKNLCCSISDFCLNYFDVYSYEYLSCMKKEFEDPSYKCFTKGGFKDKTYFAAAGALLILLLLIASRKMIK
AAZ81530.1                ISSNTCSREKSKNLCCSISDFCLNYFDVNSYEYHSCMKKEFEDPSYKCFTKGGFKDKTYFAAAGALLILLLLIASRKMIK
ABA39301.1                ISSNTCSREKSKNLCCSISDFCLNYFDVYSYEYLSCMKKEFEDPSYKCFTKGGFKDKTYFAAAGALLILLLLIASRKMIK
ACB42428.1                ISSNTCSREKSKNLCCSISDFCLNYFDVNSYEYHSCVKKEFEDPSYKCFTKGGFKDKTYFAAAGALLILLLLIASRKMIK
ACB42432                  ISSNTCSREKSKNLCCSISDFCLNYFDVHSYEYLSCMKKEFEDPSYKCFTIGGFKDKTYFAAAGALLILLLLIASRKMIK
PVL_060021400-t42_1-p1    ISSNTCSREKSKNLCCSISDFCLNYFDVYSFEYHNCMKKEFEDPSYKCFTKGGFTDNTYFAAAGALLILLLLIASRKMIK
ACB42432.1                ISSNTCSREKSKNLCCSISDFCLNYFDVHSYEYLSCMKKEFEDPSYKCFTIGGFKDKTYFAAAGALLILLLLIASRKMIK
Trans-membrane domain                                                              |<-------------->|

PCYB_063270-t26_1-p1      NDSEEATFNEFEEHCDNIQRIPLMPNNIEHMQPLTPLDYS
PcyM_0628800-t36_1-p1     NDSEEATFNEFEEHCDNIQRIPLMPNNIEHMQPLTPLDYS
PVP01_0623800.1-p1        NDSEEATFNEFEEYCDNIHRIPLMPNNIEHMQPSTPLDYS
PVX_110810.1-p1           NDSEEATFNEFEEYCDNIHRIPLMPNNIEHMQPSTPLDYS
Psim2302_000333900.1      NDSEEATFNEFEEYCDNIHRIPLMPNNIEHMQPSTPLDYS
Psim3636_000376000.1      NDSEEATFNEFEEYCDNIHRIPLMPNNIEHMQPSTPLDYS
PsimAD002_000401800.1     NDSEEATFNEFEEYCDNIHRIPLMPNNIEHMQPSTPLDYS
PsimAD005_000131600.1     NDSEEATFNEFEEYCDNIHRIPLMPNNIEHMQPSTPLDYS
PsimAF28_001016200.1      NDSEEATFNEFEEYCDNIHRIPLMPNNIEHMQPSTPLDYS
PsimAF33_000351600.1      NDSEEATFNEFEEYCDNIHRIPLMPNNIEHMQPSTPLDYS
PvivaxAM01_000437300.1    NDSEEATFNEFEEYCDNIHRIPLMPNNIEHMQPSTPLDYS
PvivaxAM02_000308000.1    NDSEEATFNEFEEYCDNIHRIPLMPNNIEHMQPSTPLDYS
AAZ81533.1                NDSEEATFNEFEEYCDNIHRIPLM----------------
AAZ81530.1                NDSEEATFNEFEEYCDNIHRIPLM----------------
ABA39301.1                NDSEEATFNAFEEYCDNIHRIPLM----------------
ACB42428.1                NDSEEATFKEFEEYCDNIHRIPLMPNNIEHMQPSTPLDYS
ACB42432                  NDSEEATFNEFEEYCDNIHRIPLMPNNIEHMQPSTPLDYS
PVL_060021400-t42_1-p1    NDSEEATFNEFEEYCDNIHRIPLMPNNIEHMQPSTPLDYS
ACB42432.1                NDSEEATFNEFEEYCDNIHRIPLMPNNIEHMQPSTPLDYS
```
