# Supplementary material for: The genome of the zoonotic malaria parasite Plasmodium simium reveals adaptations to host switching
Source: BMC Biol. 2021 Oct 1;19:219. doi: 10.1186/s12915-021-01139-5 (PMC8485552; doi:10.1186/s12915-021-01139-5)
Supplement: Supplementary file 4 — Additional file 4: Figure S25. RBP2a alignment. Complete alignment of RBP2a protein sequences. [file 12915_2021_1139_MOESM4_ESM.pdf]

Figure S25

Complete alignment of RBP2a protein sequences

```
PVX_121920.1-p1            MENKVLWAVFYNLVLFLLASSKESNRIKAYKLKKEPKLWPLQDSLNESDKFEYTNGGKENPPNFFSSNVKAHNKKEGKKY
PVP01_1402400.1-p1         MENKVLWAVFYNLVLFLLASSKESNRIKAYKLKKEPKLWPLQDSLNESDKFEYTNGGKENPPNFFSSNVKAHNKKEGKKY
Psim2302_000007000.1       MENKVLWAVFYNLVLFLLASSKESNRIKAYKLKKEPKLWPLQDSLNESDKFEYTNGGKENPPNFFSSNVKAHNKKEGKKY
Psim3636_000006300.1       MENKVLWAVFYNLVLFLLASSKESNRIKAYKLKKEPKLWPLQDSLNESDKFEYTNGGKENPPNFFSSNVKAHNKKEGKKY
PsimAD002_000007200.1      MENKVLWAVFYNLVLFLLASSKESNRIKAYKLKKEPKLWPLQDSLNESDKFEYTNGGKENPPNFFSSNVKAHNKKEGKKY
PsimAD005_000448800.1      MENKVLWAVFYNLVLFLLASSKESNRIKAYKLKKEPKLWPLQDSLNESDKFEYTNGGKENPPNFFSSNVKAHNKKEGKKY
PsimAF28_000006200.1       MENKVLWAVFYNLVLFLLASSKESNRIKAYKLKKEPKLWPLQDSLNESDKFEYTNGGKENPPNFFSSNVKAHNKKEGKKY
PsimAF33_000006700.1       MENKVLWAVFYNLVLFLLASSKESNRIKAYKLKKEPKLWPLQDSLNESDKFEYTNGGKENPPNFFSSNVKAHNKKEGKKY
PsimAF34_000005900.1       MENKVLWAVFYNLVLFLLASSKESNRIKAYKLKKEPKLWPLQDSLNESDKFEYTNGGKENPPNFFSSNVKAHNKKEGKKY
PvivaxAM01_000076800.1     MENKVLWAVFYNLVLFLLASSKESNRIKAYKLKKEPKLWPLQDSLNESDKFEYTNGGKENPPNFFSSNVKAHNKKEGKKY
PvivaxAM02_000474900.1     MENKVLWAVFYNLVLFLLASSKESNRIKAYKLKKEPKLWPLQDSLNESDKFEYTNGGKENPPNFFSSNVKAHNKKEGKKY
PVL_140006600-t42_1-p1     MENKVLWAVFYNLVLFLLASSKESNRIKAYKLKKESKLWPLQDSLNESDKFEYTNGGKENPPNFFNSNVKAHNKKEGKKY

PVX_121920.1-p1            EQNLSLPDNTSFVTVKNYNYTRTPSHHAYIRRDNTHTNSTNNNQIRNVPKELNPREFLFTPKNQISASLIQTNGPVAPMD
PVP01_1402400.1-p1         EQNLSLPDNTSFVTVKNYNYTRTPSHHAYIRRDNTHTNSTNNNQIRNVPKELNPREFLFTPKNQISASLIQTNGPVAPMD
Psim2302_000007000.1       EQNLSLPDNTSFVTVKNYNYTRTPSHHAYIRRDNTHTNSTNNNQIRNVPKELNPREFLFTPKNQISASLIQTNKPVAPMD
Psim3636_000006300.1       EQNLSLPDNTSFVTVKNYNYTRTPSHHAYIRRDNTHTNSTNNNQIRNVPKELNPREFLFTPKNQISASLIQTNKPVAPMD
PsimAD002_000007200.1      EQNLSLPDNTSFVTVKNYNYTRTPSHHAYIRRDNTHTNSTNNNQIRNVPKELNPREFLFTPKNQISASLIQTNKPVAPMD
PsimAD005_000448800.1      EQNLSLPDNTSFVTVKNYNYTRTPSHHAYIRRDNTHTNSTNNNQIRNVPKELNPREFLFTPKNQISASLIQTNKPVAPMD
PsimAF28_000006200.1       EQNLSLPDNTSFVTVKNYNYTRTPSHHAYIRRDNTHTNSTNNNQIRNVPKELNPREFLFTPKNQISASLIQTNKPVAPMD
PsimAF33_000006700.1       EQNLSLPDNTSFVTVKNYNYTRTPSHHAYIRRDNTHTNSTNNNQIRNVPKELNPREFLFTPKNQISASLIQTNKPVAPMD
PsimAF34_000005900.1       EQNLSLPDNTSFVTVKNYNYTRTPSHHAYIRRDNTHTNSTNNNQIRNVPKELNPREFLFTPKNQISASLIQTNGPVAPMD
PvivaxAM01_000076800.1     EQNLSLPDNTSFVTVKNYNYTRTPSHHAYIRRDNTHTNSTNNNQIRNVPKELNPREFLFTPKNQISASLIQTNGPVAPMD
PvivaxAM02_000474900.1     EQNLSLPDNTSFVTVKNYNYTRTPSHHAYIRRDNTHTNSTNNNQIRNVPKELNPREFLFTPKNQISASLIQTNGPVAPMD
PVL_140006600-t42_1-p1     EQNLSLPNNTSFVTVKNYNYTRTPSHHAYIRRDNTHTNSTNNNQIRNVPKELNPREFLFTPNNQISASLIQTNGPVAPMD

PVX_121920.1-p1            ILRYLDFSNSSGQIISTVYPFYVQMNYFAEIKYYITYHYEAKKNYDEAYNQSVNPLMSSIQNQINSCVPKKAALEKTIFV
PVP01_1402400.1-p1         ILRYLDFSNSSGQIISTVYPFYVQMSYFAEIKYYITYHYEAKKNYDEAYNQSVNPLMSRIQNQINSCVPKKAALEKTIFV
Psim2302_000007000.1       ILRYLDFSNSSGQIISTVYPFYVQMNYFAEIKYYITYHYEAKKKYDEAYNQSVNPLMSSIQNQINSCVPKKAALEKTIFV
Psim3636_000006300.1       ILRYLDFSNSSGQIISTVYPFYVQMNYFAEIKYYITYHYEAKKNYDEAYNQSVNPLMSSIQNQINSCVPKKAALEKTIFV
PsimAD002_000007200.1      ILRYLDFSNSSGQIISTVYPFYVQMNYFAEIKYYITYHYEAKKNYDEAYNQSVNPLMSSIQNQINSCVPKKAALEKTIFV
PsimAD005_000448800.1      ILRYLDFSNSSGQIISTVYPFYVQMNYFAEIKYYITYHYEAKKNYDEAYNQSVNPLMSSIQNQINSCVPKKAALEKTIFV
PsimAF28_000006200.1       ILRYLDFSNSSGQIISTVYPFYVQMNYFAEIKYYITYHYEAKKKYDEAYNQSVNPLMSSIQNQINSCVPKKAALEKTIFV
PsimAF33_000006700.1       ILRYLDFSNSSGQIISTVYPFYVQMNYFAEIKYYITYHYEAKKNYDEAYNQSVNPLMSSIQNQINSCVPKKAALEKTIFV
PsimAF34_000005900.1       ILRYLDFSNSSGQIISTVYPFYVQMNYFAEIKYYITYHYEAKKNYDEEYNQSVNPLMSSIQNQINSCVPKKAALEKTIFV
PvivaxAM01_000076800.1     ILRYLDFSNSSGQIISTVYPFYVQMSYFAEIKYYITYHYEAKKNYDEAYNQSVNPLMSSIQNQINSCVPKKAALEKTIFV
PvivaxAM02_000474900.1     ILRYLDFSNSSGQIISTVYPFYVQMSYFAEIKYYITYHYEAKKNYDEAYNQSVNPLMSSIQNQINSCVPKKAALEKTIFV
PVL_140006600-t42_1-p1     ILRYLDFSNSSGQIISTVYPFYVQMNYFAEIKYYITFHFEAKKNYDEAYNQSVNPLMSSIQNQINSCVPKKAALEKTIFV

PVX_121920.1-p1            LEYPENHNINLSNYEAKHNEYKQQLDAYKNCVQANMESYTDRMSKFNEKIYSILNSVKCTDACETDTYEIMLEIYVERVK
PVP01_1402400.1-p1         LEYPENHNINLSNYEAKHNEYKQQLDAYKNCVQANMKSYTDRMSNFNEKIYSILNSVKCTDACETDTYEIMLEIYVERVK
Psim2302_000007000.1       LEYPENHNINLSNYEAKHNEYKQQLDAYKNCVQANMESYTDRMSEFNEKIYSILNSVKCTDACETVTYKIMVEIYVERVN
Psim3636_000006300.1       LEYPENHNINLSNYEAKHNEYKQQLDAYKNCVQANMESYTDRMSEFNEKIYSILNSVKCTDACETVTYKIMVEIYVERVN
PsimAD002_000007200.1      LEYPENHNINLSNYEAKHNEYKQQLDAYKNCVQANMESYTDRMSKFNEKIYSILNSVKCTDACKTVMYKIMLEIYVERVK
PsimAD005_000448800.1      LEYPENHNINLSNYEAKHNEYKQQLDAYKNCVQANMESYTDRMSKFNEKIYSILNSVKCTDACKTVMYKIMLEIYVERVK
PsimAF28_000006200.1       LEYPENHNINLSNYEAKHNEYKQQLDAYKNCVQANMESYTDRMSKFNEKIYSILNSVKCTDACKTVMYKIMLEIYVERVK
PsimAF33_000006700.1       LEYPENHNINLSNYEAKHNEYKQQLDAYKNCVQANMESYTDRMSKFNEKIYSILNSVKCTDACKTVMYKIMLEIYVERVK
PsimAF34_000005900.1       LEYPENHNINLSNYEAKHNEYKQQLDAYKNCVQANMESYTDRMSKFNEKIYSILNSVKCTDACKTVMYKIMLEIYVERVK
PvivaxAM01_000076800.1     LEYPENHNINLSNYEAKHNEYKQQLDAYKNCVQANMKSYTDRMSKFNEEIYSILNSVKCTDACKTVTYEIMLEIYVERVK
PvivaxAM02_000474900.1     LEYPENHNINLSNYEAKHNEYKQQLDAYKNCVQANMKSYTDRMSKFNEEIYSILNSVKCTDACKTVTYEIMLEIYVERVK
PVL_140006600-t42_1-p1     LENPESHNINLSNYEEKHNEYKQQLDAYKNCVQANMESYTDRMSKFNEKIYSILNSVKCTDACETDTYEIMLEIYVERVK

PVX_121920.1-p1            EVNHNNYVNYLSTLKASLQLGVTLMLKVKQEIDNNVTISAINFLQEEMLDIITIGEAHTGKIIHGKENVLKLQNNNIPPQ
PVP01_1402400.1-p1         EVNHNNYVNYLSTLKASLQLGVTLMLKVKQEIDNNVTISAINFLQEEMLDIITIGEAHTGKIIHGKENVLKLQNNNIPSQ
Psim2302_000007000.1       EVNHNNYVNYLSTLKASLQLGVTLMLKVKQEIDNNVTISAINFLQEEMLDIITIGEAHTGKIIHGKENVLKLQNNNIPPK
Psim3636_000006300.1       EVNHNNYVNYLSTLKASLQLGVTLMLKVKQEIDNNVTISAINFLQEEMLDIITIGEAHTGKIIHGKENVLKLQNNNIPPK
PsimAD002_000007200.1      EVNHNNYVNYLSTLKASLQLGVTLMLKVKQEIDNNVTISAINFLQEEMLDIITIGEAHTGKIIHGKENVLKLQNNNIPPK
PsimAD005_000448800.1      EVNHNNYVNYLSTLKASLQLGVTLMLKVKQEIDNNVTISAINFLQEEMLDIITIGEAHTGKIIHGKENVLKLQNNNIPPK
PsimAF28_000006200.1       EVNHNNYVNYLSTLKASLQLGVTLMLKVKQEIDNNVTISAINFLQEEMLDIITIGEAHTGKIIHGKENVLKLQNNNIPPK
PsimAF33_000006700.1       EVNHNNYVNYLSTLKASLQLGVTLMLKVKQEIDNNVTISAINFLQEEMLDIITIGEAHTGKIIHGKENVLKLQNNNIPPK
PsimAF34_000005900.1       EVNHNNYVNYLSTLKASLQLGVTLMLKVKQEIDNNVTISAINFLQEEMLDIITIGEAHTGKIIHGKENVLKLQNNNIPPK
PvivaxAM01_000076800.1     EVNHNNYVNYLSTLKASLQLGVTLMLKVKQEIDNNVTISAINFLQEEMLDIITIGEAHTGKIIHGKENVLKLQNNNIPSQ
PvivaxAM02_000474900.1     EVNHNNYVNYLSTLKASLQLGVTLMLKVKQEIDNNVTISAINFLQEEMLDIITIGEAHTGKIIHGKENVLKLQNNNIPSQ
PVL_140006600-t42_1-p1     EVNLNNYVNYLSTLKASLQFGVTLMLKVKQEIDNNVTISTINFLQEEMLDIITICEAHTGKIIHGKENVLKLQGKEIPAE

PVX_121920.1-p1            VPLSTLKKLYFDSANFYATYKFSLKRADTTTAALKEKGKLLANLYNKLITYVSEKIDKNLDSLYFISKSESMISEFEDTF
PVP01_1402400.1-p1         VPLSTLKKLYFDSANFYATYKFSLKRADTTTAALKEKEKLLANLYNKLITYVSEKIDKNLDSLYFISKSESMISEFEDTF
Psim2302_000007000.1       VPLSTLKKLYFDSANFYATYKFSLKRADTTTAALKEKGILLANLYNKLITYVSEKIDKNLDSLYFISKSESMISEFEDTF
Psim3636_000006300.1       VPLSTLKKLYFDSANFYATYKFSLKRADTTTAALKKKRKLLRNLYKKLITYVSEKIDKNLDSLYFISKSESMISEFEDTF
PsimAD002_000007200.1      VPLSTLKKLYFDSANFYATYKFSLKRADTTTAALKKKRKLLRNLYKKLITYVSEKIDKNLDSLYFISKSESMISEFEDTF
PsimAD005_000448800.1      VPLSTLKKLYFDSANFYATYKFSLKRADTTTAALKKKRKLLRNLYKKLITYVSEKIDKNLDSLYFISKSESMISEFEDTF
PsimAF28_000006200.1       VPLSTLKKLYFDSANFYATYKFSLKRADTTTAALKKKRKLLRNLYKKLITYVSEKIDKNLDSLYFISKSESMISEFEDTF
PsimAF33_000006700.1       VPLSTLKKLYFDSANFYATYKFSLKRADTTTAALKEKGILLANLYNKLITYVSEKIDKNLDSLYFISKSESMISEFEDTF
PsimAF34_000005900.1       VPLSTLKKLYFDSANFYATYKFSLKRADTTTAALKEKGILLANLYNKLITYVSEKIDKNLDSLYFISKSESMISEFEDTF
PvivaxAM01_000076800.1     VPLSTLKKLYFDSANFYATYKFSLKRADTTTAALKEKGKLLANLYNKLITYVSEKIDKNLDSLYFISKSESMISEFEDTF
PvivaxAM02_000474900.1     VPLSTLKKLYFDSANFYATYKFSLKRADTTTAALKEKGKLLANLYNKLITYVSEKIDKNLDSLYFISKSESMISEFEDTF
PVL_140006600-t42_1-p1     VPLSTLKKLYFDSANFYATYKFSLKRTDTTTAALREKGKLLADLYNKLITYVSEKIDKNLDSSYFISKSESMISEFEDTF
```

Figure S25 (cont.)

```
PVX_121920.1-p1            KAGTEASKENSSITADYARYSNLQIIDIKSKYDYKITELKKYLNQLKVLISPMKDLYNLNSLQKDQRDSVRSIRSSDSSP
PVP01_1402400.1-p1         KAGTEASKENSSITADYARYSNLQIIDIKSMYDYKITELKKYLNQLKVLISPMKDLYNLNSLQKDQRDSVRSIRSSNSSP
Psim2302_000007000.1       KAGTEASKENSSITADYARYSNLQIIDIKSKYDYKITELKKYLNQLKVLISPMKDLYNLNSLQKDQRDSVRSIRSSNSSP
Psim3636_000006300.1       KAGTEASKENSSITADYARYSNLQIIDIKSKYDYKITELKKYLNQLKVLISPMKDLYNLNSLQKDQRDSVRSIRSSNSSP
PsimAD002_000007200.1      KAGTEASKENSSITADYARYSNLQIIDIKSKYDYKITELKKYLNQLKVLISPMKDLYNLNSLQKDQRDSVRSIRSSNSSP
PsimAD005_000448800.1      KAGTEASKENSSITADYARYSNLQIIDIKSKYDYKITELKKYLNQLKVLISPMKDLYNLNSLQKDQRDSVRSIRSSNSSP
PsimAF28_000006200.1       KAGTEASKENSSITADYARYSNLQIIDIKSKYDYKITELKKYLNQLKVLISPMKDLYNLNSLQKDQRDSVRSIRSSNSSP
PsimAF33_000006700.1       KAGTEASKENSSITADYARYSNLQIIDIKSKYDYKITELKKYLNQLKVLISPMKDLYNLNSLQKDQRDSVRSIRSSNSSP
PsimAF34_000005900.1       KAGTEASKENSSITADYARYSNLQIIDIKSKYDYKITELKKYLNQLKVLISPMKDLYNLNSLQKDQRDSVRSIRSSNSSP
PvivaxAM01_000076800.1     KAGTEASKENSSITADYARYSNLQIIDIKSKYDYKITELKKYLNQLKVLISPMKDLYNLNSLQKDQRDSVRSIRSSDSSP
PvivaxAM02_000474900.1     KAGTEASKENSSITADYARYSNLQIIDIKSKYDYKITELKKYLNQLKVLISPMKDLYNLNSLQKDQRDSVRSIRSSDSSP
PVL_140006600-t42_1-p1     KAGTEASKENPSITADYARYSNLQIIDIKSKYDYKITELKKYLNQLKVLISPMKDLYNLNSLQKDQRDSGRSIRSSNSSP

PVX_121920.1-p1            QEKVSALLNFLSTIKEENGTVLENFKNIERYYEQGKPLKGTLEGLIKMIKTYEQDIVNLIDNEKHNRSYKDDIKNKIIYI
PVP01_1402400.1-p1         QEKVSALLNFLSKIKEENGTVLENFKNIERYYEQGKPLKGTLEGLIKMIKTYEQDIVNLIDNEKHNRSYKDDIKNKIIYI
Psim2302_000007000.1       QEKVSALLNFLSKIKEEYGTVLENFKNIERYYEQGKPLKGTLEGLIKMIKAYEQDIVNLIDNEKHNRSYKDDIKNKIIYI
Psim3636_000006300.1       QEKVSALLNFLSTIKKEYGTVLENFKNIERYYEQGKPLKGTLEGLIKMIKAYEQDIVNLIDNEKHNRSYKDDIKNKIIYI
PsimAD002_000007200.1      QEKVSALLNFLSTIKKEYGTVLENFKNIERYYEQGKPLKGTLEGLIKMIKAYEQDIVNLIDNEKHNRSYKDDIKNKIIYI
PsimAD005_000448800.1      QEKVSALLNFLSTIKKEYGTVLENFKNIERYYEQGKPLKGTLEGLIKMIKAYEQDIVNLIDNEKHNRSYKDDIKNKIIYI
PsimAF28_000006200.1       QEKVSALLNFLSTIKKEYGTVLENFKNIERYYEQGKPLKGTLEGLIKMIKAYEQDIVNLIDNEKHNRSYKDDIKNKIIYI
PsimAF33_000006700.1       QEKVSALLNFLSKIKEEYGTVLENFKNIERYYEQGKPLKGTLEGLIKMIKAYEQDIVNLIDNEKHNRSYKDDIKNKIIYI
PsimAF34_000005900.1       QEKVSALLNFLSKIKEEYGTVLENFKNIERYYEQGKPLKGTLEGLIKMIKAYEQDIVNLIDNEKHNRSYKDDIKNKIIYI
PvivaxAM01_000076800.1     QEKVSALLNFLSTIKEENGTVLENFKNIERYYEQGKPLKGTLEGLIKMIKTYEQDIVNLIDNEKHNRSYKDDIENKIIYI
PvivaxAM02_000474900.1     QEKVSALLNFLSTIKEENGTVLENFKNIERYYEQGKPLKGTLEGLIKMIKAYEQDIVNLIDNEKHNRSYKDDIENKIIYI
PVL_140006600-t42_1-p1     QKKVSALINLLSTIKDEDGTVLENFKNIERYYEQGKPLKGTLEGLIKMIKAYEQDIVNLIDNEKHNRSYKDDIKNKIKYI

PVX_121920.1-p1            TEKTDAIKDIIPLNEEVDNIIKQIEKLFNEDLFHEGLPNMEKLKKEKEKIKATIKSLNTNFYDGDLKELVDDMLNFVYKH
PVP01_1402400.1-p1         TEKTDAIKDIIPLNEEVDNIIKQIEKLFNEDLFHEGLPNMEKLKKEKEKIKATIKSLNTNFYDGDLKELVDDMLNFVYKH
Psim2302_000007000.1       TEKTDAIKDIIPLNEEVDNIIKQIEKLFNEDLFHEGLPNMEKLKKEKEKIKATIKSLNTNFYDGDLKELVDDMLNFVYKH
Psim3636_000006300.1       TEKTDAIKDIIPLNEEVDNIIKQIEKLFNEDLFHEGLPNMEKLKKEKEKIKATIKSLNTNFYDGDLKELVDDMLNFVYKH
PsimAD002_000007200.1      TEKTDAIKDIIPLNEEVDNIIKQIEKLFNEDLFHEGLPNMEKLKKEKEKIKATIKSLNTNFYDGDLKELVDDMLNFVYKH
PsimAD005_000448800.1      TEKTDAIKDIIPLNEEVDNIIKQIEKLFNEDLFHEGLPNMEKLKKEKEKIKATIKSLNTNFYDGDLKELVDDMLNFVYKH
PsimAF28_000006200.1       TEKTDAIKDIIPLNEEVDNIIKQIEKLFNEDLFHEGLPNMEKLKKEKEKIKATIKSLNTNFYDGDLKELVDDMLNFVYKH
PsimAF33_000006700.1       TEKTDAIKDIIPLNEEVDNIIKQIEKLFNEDLFHEGLPNMEKLKKEKEKIKATIKSLNTNFYDGDLKELVDDMLNFVYKH
PsimAF34_000005900.1       TEKTDAIKDIIPLNEEVDNIIKQIEKLFNEDLFHEGLPNMEKLKKEKEKIKATIKSLNTNFYDGDLKELVDDMLNFVYKH
PvivaxAM01_000076800.1     TEKTDAIKDIIPLNEEVDNIIKQIEKLFNEDLFHEGLPNMEKLKKEKEKIKATIKSLNTNFYDGDLKELVDDMLNFVYKH
PvivaxAM02_000474900.1     TEKTDAIKDIIPLNEEVDNIIKQIEKLFNEDLFHEGLPNMEKLKKEKEKIKATIKSLNTNFYDGDLKELVDDMLNFVYKH
PVL_140006600-t42_1-p1     TEKTDAIKDIIPLNEEVDNIIKQIEKLFNEDLFHEGLPNMEKLKKEKEKIKATIKSLNTNFYDGDLKELVDDMLNFVYKH

PVX_121920.1-p1            SDFYNQAYAKGDVVNLQKEAEQEYEKITKLKSNNIPQMLKDLKSESKNLSELKDTMMDEMLNNVHQDISNVFEQIRNKYK
PVP01_1402400.1-p1         SDFYDQAYAKGDVVNLQKEAEQEYEKITKLKSNNIPQMLKDLKSESKNLSELKDTMMDEMLNNVHQDISNVFEQIRNKYK
Psim2302_000007000.1       SDFYDQAYDKGDVVNLQKEAEQEYEKITKLKSNNIPQMLKDLKSESKNLSELKDTMMDEMLNNVHQDISNVFEQIRNKYK
Psim3636_000006300.1       SDFYDQAYDKGDVVNLQKEAEQEYEKITKLKSNNIPQMLKDLKSESKNLSELKDTMMDEMLNNVHQDISNVFEQIRNKYK
PsimAD002_000007200.1      SDFYDQAYDKGDVVNLQKEAEQEYEKITKLKSNNIPQMLKDLKSESKNLSELKDTMMDEMLNNVHQDISNVFEQIRNKYK
PsimAD005_000448800.1      SDFYDQAYDKGDVVNLQKEAEQEYEKITKLKSNNIPQMLKDLKSESKNLSELKDTMMDEMLNNVHQDISNVFEQIRNKYK
PsimAF28_000006200.1       SDFYDQAYDKGDVVNLQKEAEQEYEKITKLKSNNIPQMLKDLKSESKNLSELKDTMMDEMLNNVHQDISNVFEQIRNKYK
PsimAF33_000006700.1       SDFYDQAYDKGDVVNLQKEAEQEYEKITKLKSNNIPQMLKDLKSESKNLSELKDTMMDEMLNNVHQDISNVFEQIRNKYK
PsimAF34_000005900.1       SDFYDQAYDKGDVVNLQKEAEQEYEKITKLKSNNIPQMLKDLKSESKNLSELKDTMMDEMLNNVHQDISNVFEQIRNKYK
PvivaxAM01_000076800.1     SDFYDQAYAKGDVVNLQKEAEQEYEKITKLKSNNIPQMLKDLKSESKNLSELKDTMMDEMLNNVHQDISNVFEQIRNKYK
PvivaxAM02_000474900.1     SDFYDQAYAKGDVVNLQKEAEQEYEKITKLKSNNIPQMLKDLKSESKNLSELKDTMMDEMLNNVHQDISNVFEQIRNKYK
PVL_140006600-t42_1-p1     SDFYDQAYAKGDVVNLQKEAEQEYEKITKLKSNNIPQILKDLKSESKNLSELKDTMMDEMLNNVHQDISNAFEQIRNKYK

PVX_121920.1-p1            DITTSLSAYKEDKQKFEKFKVDMIQTKNELLGSLQQNDKKLSEANNMYEEFLAHIDTTTNREKQMYNQANALKENLANVQ
PVP01_1402400.1-p1         DITTSLSAYKEDKQKFEKFKVDMIQTKNELLGSLQQNDKKLSEANNMYEEFLAHIDTTTNREKQMYNQANALKENLANVQ
Psim2302_000007000.1       DITTSLSAYKEDKQKFEKFKVDMIQTKNELVGSLQQNDKKLSEANNMYEEFLAHIDTTTNREKQMYNQANALKENLANVQ
Psim3636_000006300.1       DITTSLSAYKEDKQKFEKFKVDMIQTKNELVGSLQQNDKKLSEANNMYEEFLAHIDTTTNREKQMYNQANALKENLANVQ
PsimAD002_000007200.1      DITTSLSAYKEDKQKFEKFKVDMIQTKNELVGSLQQNDKKLSEANNMYEEFLAHIDTTTNREKQMYNQANALKENLANVQ
PsimAD005_000448800.1      DITTSLSAYKEDKQKFEKFKVDMIQTKNELVGSLQQNDKKLSEANNMYEEFLAHIDTTTNREKQMYNQANALKENLANVQ
PsimAF28_000006200.1       DITTSLSAYKEDKQKFEKFKVDMIQTKNELVGSLQQNDKKLSEANNMYEEFLAHIDTTTNREKQMYNQANALKENLANVQ
PsimAF33_000006700.1       DITTSLSAYKEDKQKFEKFKVDMIQTKNELVGSLQQNDKKLSEANNMYEEFLAHIDTTTNREKQMYNQANALKENLANVQ
PsimAF34_000005900.1       DITTSLSAYKEDKQKFEKFKVDMIQTKNELVGSLQQNDKKLSEANNMYEEFLAHIDTTTNREKQMYNQANALKENLANVQ
PvivaxAM01_000076800.1     DITTSLSAYKEDKQKFEKFKVDMIQTKNELLGSLQQNDKKLSEANNMYEEFLAHIDTTTNREKQMYNQANALKENLANVQ
PvivaxAM02_000474900.1     DITTSLSAYKEDKQKFEKFKVDMIQTKNELLGSLQQNDKKLSEANNMYEEFLAHIDTTTNREKQMYNQANALKENLANVQ
PVL_140006600-t42_1-p1     DITTSLSAYKEDKQKFEKFKVDMIQTKNELLGSLQQNDKKLSEANNMYEEFLAHIDTTTNRENQMYNQANALKENLANVQ

PVX_121920.1-p1            SKISSYQGKIEKLKAHTRKNYEKIKTLLQTFGNENINAKLAEYEKEFNENKRVVNTIINQIQESNKNIENIKILNNSIDR
PVP01_1402400.1-p1         SKISSYQGKIEKLKAHTRKNYEKIKTLLQTFGNENINAKLAEYEKEFNENKRVVNTIINQIQESNKNIENIKILNNSIDR
Psim2302_000007000.1       SKISSYQGKIEKLKAHTRKNYEKIKTLLQTFGNENINAKLAEYEKEFNENKRVVNTIINQIQESNKNIENIKILNNSIDR
Psim3636_000006300.1       SKISSYQGKIEKLKAHTRKNYEKIKTLLQTFGNENINAKLAEYEKEFNENKRVVNTIINQIQESNKNIENIKILNNSIDR
PsimAD002_000007200.1      SKISSYQGKIEKLKAHTRKNYEKIKTLLQTFGNENINAKLAEYEKEFNENKRVVNTIINQIQESNKNIENIKILNNSIDR
PsimAD005_000448800.1      SKISSYQGKIEKLKAHTRKNYEKIKTLLQTFGNENINAKLAEYEKEFNENKRVVNTIINQIQESNKNIENIKILNNSIDR
PsimAF28_000006200.1       SKISSYQGKIEKLKAHTRKNYEKIKTLLQTFGNENINAKLAEYEKEFNENKRVVNTIINQIQESNKNIENIKILNNSIDR
PsimAF33_000006700.1       SKISSYQGKIEKLKAHTRKNYEKIKTLLQTFGNENINAKLAEYEKEFNENKRVVNTIINQIQESNKNIENIKILNNSIDR
PsimAF34_000005900.1       SKISSYQGKIEKLKAHTRKNYEKIKTLLQTFGNENINAKLAEYEKEFNENKRVVNTIINQIQESNKNIENIKILNNSIDR
PvivaxAM01_000076800.1     SKISSYQGKIEKLKAHTRKNYEKIKTLLQTFGNENINAKLAEYEKEFNENKEVVNTIINQIQESNKNIENIKILNNSIDR
PvivaxAM02_000474900.1     SKISSYQGKIEKLKAHTRKNYEKIKTLLQTFGNENINAKLAEYEKEFNENKEVVNTIINQIQESNKNIENIKILNNSIDR
PVL_140006600-t42_1-p1     SKISSYQGKIEKLKAHTRKNYEKIKTLLQTFGNENINAKLAEYEKEFNENKRVVNTIINQIQESNKNIENIKILNNSIDR
```

Figure S25 (cont.)

```
PVX_121920.1-p1            SEVNKQLIEELATNKQQLKEKIDSHIKQLNSYNIIAKDDKLIFEKRLNEEKANINTQLSDTSIDNLKAQIQETLDYYKTA
PVP01_1402400.1-p1         SEVNKQLIEELATNKQQLKEKIDSHIKQLNSYNIIAKDDKLIFEKTLNEEKANINTQLSDTSIDNLKAQIQETLDYYKTA
Psim2302_000007000.1       SEVNKQLIEELATNKQQLKEKIDSHIKQLNSYNIIAKDDKLIFEKTLNEEKANINTQLSDTSIDNLKAQIQETLDYYKTA
Psim3636_000006300.1       SEVNKQLIEELATNKQQLKEKIDSHIKQLNSYNIIAKDDKLIFEKTLNEEKANINTQLSDTSIDNLKAQIQETLDYYKTA
PsimAD002_000007200.1      SEVNKQLIEELATNKQQLKEKIDSHIKQLNSYNIIAKDDKLIFEKTLNEEKANINTQLSDTSIDNLKAQIQETLDYYKTA
PsimAD005_000448800.1      SEVNKQLIEELATNKQQLKEKIDSHIKQLNSYNIIAKDDKLIFEKTLNEEKANINTQLSDTSIDNLKAQIQETLDYYKTA
PsimAF28_000006200.1       SEVNKQLIEELATNKQQLKEKIDSHIKQLNSYNIIAKDDKLIFEKTLNEEKANINTQLSDTSIDNLKAQIQETLDYYKTA
PsimAF33_000006700.1       SEVNKQLIEELATNKQQLKEKIDSHIKQLNSYNIIAKDDKLIFEKTLNEEKANINTQLSDTSIDNLKAQIQETLDYYKTA
PsimAF34_000005900.1       SEVNKQLIEELATNKQQLKEKIDSHIKQLNSYNIIAKDDKLIFEKTLNEEKANINTQLSDTSIDNLKAQIQETLDYYKTA
PvivaxAM01_000076800.1     SEVNKQLIEELATNKQQLKEKIDSHIKQLNSYNIIAKDDKLIFEKTLNEEKANINTQLSDTSIDNLKAQIQETLDYYKTA
PvivaxAM02_000474900.1     SEVNKQLIEELATNKQQLKEKIDSHIKQLNSYNIIAKDDKLIFEKTLNEEKANINTQLSDTSIDNLKAQIQETLDYYKTA
PVL_140006600-t42_1-p1     SEVNKQLIEELATNKQQLKEKIDNHIKQLNSYNIIAKDDKLIFEKTLDEEKANINTQLSDTSIDNLKAQIQEMLDYCKTA

PVX_121920.1-p1            KANTNDKDGTHLEKLDKKKMDWKDSKVKIDKLSTSYQVLDKKINDLIKKQHDEIVALIDKLITEKGNEIREKTDEKIKHL
PVP01_1402400.1-p1         KANTNDKDGTHLEKLDKKKMDWKDSKVKIDILSTSYQVLDKKINDLIKKQHDEIVALIDKLITEKGNEIREKTDEKIKHL
Psim2302_000007000.1       KANTNDKDGTHLEKLDKKKMDWKDSKVKIDKLSTSYQVLDKKINDLIKKQHDEIVALIDKLITEKGNEIREKTDEKIKHL
Psim3636_000006300.1       KANTNDKDGTHLEKLDKKKMDWKDSKVKIDKLSTSYQVLDKKINDLIKKQHDEIVALIDKLITEKGNEIREKTDEKIKHL
PsimAD002_000007200.1      KANTNDKDGTHLEKLDKKKMDWKDSKVKIDKLSTSYQVLDKKINDLIKKQHDEIVALIDKLITEKGNEIREKTDEKIKHL
PsimAD005_000448800.1      KANTNDKDGTHLEKLDKKKMDWKDSKVKIDKLSTSYQVLDKKINDLIKKQHDEIVALIDKLITEKGNEIREKTDEKIKHL
PsimAF28_000006200.1       KANTNDKDGTHLEKLDKKKMDWKDSKVKIDKLSTSYQVLDKKINDLIKKQHDEIVALIDKLITEKGNEIREKTDEKIKHL
PsimAF33_000006700.1       KANTNDKDGTHLEKLDKKKMDWKDSKVKIDKLSTSYQVLDKKINDLIKKQHDEIVALIDKLITEKGNEIREKTDEKIKHL
PsimAF34_000005900.1       KANTNDKDGTHLEKLDKKKMDWKDSKVKIDKLSTSYQVLDKKINDLIKKQHDEIVALIDKLITEKGNEIREKTDEKIKHL
PvivaxAM01_000076800.1     KANTNDKDGTHLEKLDKKKMDWKDSKVKIDKLSTSYQVLDKKINDLIKKQHDEIVALIDKLITEKGNEIREKTDEKIKHL
PvivaxAM02_000474900.1     KANTNDKDGTHLEKLDKKKMDWKDSKVKIDKLSTSYQVLDKKINDLIKKQHDEIVALIDKLITEKGNEIREKTDEKIKHL
PVL_140006600-t42_1-p1     KAKTNDKDGTHLEKLNKQKMDWKDSKVKIDKLSTSYQVLDKKINDLIKKQHDEIVALIDKLITEKGNEIREKTDEKIKHL

PVX_121920.1-p1            SEIKTKLSSFTVSDNVKNTQNVAIKGEISGFEKKMEALLRRIDNNNAKLTELRGKADQHVTTLRGEKNKTAEFNAKKDSL
PVP01_1402400.1-p1         SEIKTKLSSFTVSDNVKNTQNVAIKGEISGFEKKMEALLRRIDNNNAKLTELRGKADQHVTTLRGEKNKTAEFNAKKDSL
Psim2302_000007000.1       SEIKTKLSSFTVSDNVKNTQNVAIKGEISGFEKKMEALLRRIDNNNAKLTELRGKADQHVTTLRGEKNKTAEFNAKKDSL
Psim3636_000006300.1       SEIKTKLSSFTVSDNVKNTQNVAIKGEISGFEKKMEALLRRIDNNNAKLTELRGKADQHVTTLRGEKNKTAEFNAKKDSL
PsimAD002_000007200.1      SEIKTKLSSFTVSDNVKNTQNVAIKGEISGFEKKMEALLRRIDNNNAKLTELRGKADQHVTTLRGEKNKTAEFNAKKDSL
PsimAD005_000448800.1      SEIKTKLSSFTVSDNVKNTQNVAIKGEISGFEKKMEALLRRIDNNNAKLTELRGKADQHVTTLRGEKNKTAEFNAKKDSL
PsimAF28_000006200.1       SEIKTKLSSFTVSDNVKNTQNVAIKGEISGFEKKMEALLRRIDNNNAKLTELRGKADQHVTTLRGEKNKTAEFNAKKDSL
PsimAF33_000006700.1       SEIKTKLSSFTVSDNVKNTQNVAIKGEISGFEKKMEALLRRIDNNNAKLTELRGKADQHVTTLRGEKNKTAEFNAKKDSL
PsimAF34_000005900.1       SEIKTKLSSFTVSDNVKNTQNVAIKGEISGFEKKMEALLRRIDNNNAKLTELRGKADQHVTTLRGEKNKTAEFNAKKDSL
PvivaxAM01_000076800.1     SEIKTKLSSFTVSDNVKNTQNVAIKGEISGFEKKMEALLRRIDNNNAKLTELRGKADQHVTTLRGEKNKTAEFNAKKDSL
PvivaxAM02_000474900.1     SEIKTKLSSFTVSDNVKNTQNVAIKGEISGFEKKMEALLRRIDNNNAKLTELRGKADQHVTTLRGEKNKTAEFNAKKDSL
PVL_140006600-t42_1-p1     SEIKTKLSSFTVSDNVKNTQNVAIKGEVNGFEKKIEALLKRVDNNNAKLTELRGKADQHVTTLRGEKNKTAEFNAKKDSL

PVX_121920.1-p1            EKIHQQMENTHKELESMENEKIPINDINQMEIEYARILIHHMVQQIGEKKERSKIILEEIKTTIASIDQAKKNIPPEQQD
PVP01_1402400.1-p1         EKIHQQMENTHKELESMENEKIPINDINQMEIEYARILIHHMVQQIGEKKERSKIILEEIKTTIASIDQAKKNIPPEQQD
Psim2302_000007000.1       EKIHQQMENTHKELESMENEKIPINDINQMEIEYARILIHHMVQQIGEKKERSKIILEEIKTTIASIDQAKKNIPPEQQD
Psim3636_000006300.1       EKIHQQMENTHKELESMENEKIPINDINQMEIEYARILIHHMVQQIGEKKERSKIILEEIKTTIASIDQAKKNIPPEQQD
PsimAD002_000007200.1      EKIHQQMENTHKELESMENEKIPINDINQMEIEYARILIHHMVQQIGEKKERSKIILEEIKTTIASIDQAKKNIPPEQQD
PsimAD005_000448800.1      EKIHQQMENTHKELESMENEKIPINDINQMEIEYARILIHHMVQQIGEKKERSKIILEEIKTTIASIDQAKKNIPPEQQD
PsimAF28_000006200.1       EKIHQQMENTHKELESMENEKIPINDINQMEIEYARILIHHMVQQIGEKKERSKIILEEIKTTIASIDQAKKNIPPEQQD
PsimAF33_000006700.1       EKIHQQMENTHKELESMENEKIPINDINQMEIEYARILIHHMVQQIGEKKERSKIILEEIKTTIASIDQAKKNIPPEQQD
PsimAF34_000005900.1       EKIHQQMENTHKELESMENEKIPINDINQMEIEYARILIHHMVQQIGEKKERSKIILEEIKTTIASIDQAKKNIPPEQQD
PvivaxAM01_000076800.1     EKIHQQMENTHKELESMENEKIPINDINQMEIEYARILIHHMVQQIGEKKERSKIILEEIKTTIASIDQAKKNIPPEQQD
PvivaxAM02_000474900.1     EKIHQQMENTHKELESMENEKIPINDINQMEIEYARILIHHMVQQIGEKKERSKIILEEIKTTIASIDQAKKNIPPEQQD
PVL_140006600-t42_1-p1     EKIHQQMENTHKELESMENEKIPINDINQMEIEYGRILIHHMVQQIGEKKERSKSILEEITSTIESIEQAKQNIPPEQQD

PVX_121920.1-p1            ILREFEYSGYRDKATASSTEIEQIDKSAKAEEGKANSSTNLSDIDVIKKQVLSYFRKATTESNTMENALREIKNVNTFLT
PVP01_1402400.1-p1         ILREFEYSGYRDKATASSTEIEQIDKSAKAEEGKANSSTNLSDIDVIKKQVLSYFRKATTESNTMENALREIKNVNTFLT
Psim2302_000007000.1       ILREFEYSGYRDKATASSTEIEQIDKSAKAEEGKANSSTNLSDIDVIKKQVLSYFRKATTESNTMENALREIKNVNTFLT
Psim3636_000006300.1       ILREFEYSGYRDKATASSTEIEQIDKSAKAEEGKANSSTNLSDIDVIKKQVLSYFRKATTESNTMENALREIKNVNTFLT
PsimAD002_000007200.1      ILREFEYSGYRDKATASSTEIEQIDKSAKAEEGKANSSTNLSDIDVIKKQVLSYFRKATTESNTMENALREIKNVNTFLT
PsimAD005_000448800.1      ILREFEYSGYRDKATASSTEIEQIDKSAKAEEGKANSSTNLSDIDVIKKQVLSYFRKATTESNTMENALREIKNVNTFLT
PsimAF28_000006200.1       ILREFEYSGYRDKATASSTEIEQIDKSAKAEEGKANSSTNLSDIDVIKKQVLSYFRKATTESNTMENALREIKNVNTFLT
PsimAF33_000006700.1       ILREFEYSGYRDKATASSTEIEQIDKSAKAEEGKANSSTNLSDIDVIKKQVLSYFRKATTESNTMENALREIKNVNTFLT
PsimAF34_000005900.1       ILREFEYSGYRDKATASSTEIEQIDKSAKAEEGKANSSTNLSDIDVIKKQVLSYFRKATTESNTMENALREIKNVNTFLT
PvivaxAM01_000076800.1     ILREFEYSGYRDKATASSTEIEQIDKSAKAEEGKANSSTNLSDIDVIKKQVLSYFRKATTESNTMENALREIKNVNTFLT
PvivaxAM02_000474900.1     ILREFEYSGYRDKATASSTEIEQIDKSAKAEEGKANSSTNLSDIDVIKKQVLSYFRKATTESNTMENALREIKNVNTFLT
PVL_140006600-t42_1-p1     ILHEFEYSGYRDKATASSTEIEQIDKSAKTEEGKANSSTNLSDIDVIKKEVLSYLRKATTESNTMEDALSEIKNVNTFLT

PVX_121920.1-p1            SNISEEILANILKSAARTQELNQQAVGEFNKTDRLIKEVEAKLSQANEHKSAISGSVEYKQIEQKINLIKQIQKEITAGK
PVP01_1402400.1-p1         SNISEEILANILKSAARTQELNQQAVGEFNKTDRLIKEVEAKLSQANEHKSAISGSVEYKQIEQKINLIKQIQKEITAGK
Psim2302_000007000.1       SKNSEEILA-----------------------------------------------------------------------
Psim3636_000006300.1       SKNSEEILA-----------------------------------------------------------------------
PsimAD002_000007200.1      SKNSEEILA-----------------------------------------------------------------------
PsimAD005_000448800.1      SKNSEEILA-----------------------------------------------------------------------
PsimAF28_000006200.1       SKNSEEILA-----------------------------------------------------------------------
PsimAF33_000006700.1       SKNSEEILA-----------------------------------------------------------------------
PsimAF34_000005900.1       SKNSEEILA-----------------------------------------------------------------------
PvivaxAM01_000076800.1     SNISEEILANILKSAARTQELNQQAVGEFNKTDRLIKEVEAKLSQANEHKSAISGSVEYKQIEQKINLIKQIQKEITAGK
PvivaxAM02_000474900.1     SNISEEILANILKSAARTQELNQQAVGEFNKTDRLIKEVEAKLSQANEHKSAISGSVEYKQIEQKINLIKQIQKEITAGK
PVL_140006600-t42_1-p1     SNISEEILANILKSAARTQELNQQAVGEFNKADRLIKEVEAKLSQATEHKSAIPGSAEYKQIEQKINLIKQIQKEITAGK
```

Figure S25 (cont.)

```
PVX_121920.1-p1            EEINNCLSNTKEYKEKCESEVNSVNRGKAKVDFLQKREALEK-KMSQENLGKITDSIDQCKKDLADITSLEVKVKANYDS
PVP01_1402400.1-p1         EEINNCLSNTKEYKEKCESEVNSVNRGKAKVDFLQKREALEK-KMSQENLGKITDSIDQCKKDLADITSLEVKVKANYDS
Psim2302_000007000.1       --------------------------------------------------------------------------------
Psim3636_000006300.1       --------------------------------------------------------------------------------
PsimAD002_000007200.1      --------------------------------------------------------------------------------
PsimAD005_000448800.1      --------------------------------------------------------------------------------
PsimAF28_000006200.1       --------------------------------------------------------------------------------
PsimAF33_000006700.1       --------------------------------------------------------------------------------
PsimAF34_000005900.1       --------------------------------------------------------------------------------
PvivaxAM01_000076800.1     EEINNCLSNTKEYKEKCESEVNSVNRGKAKVDFLQKREALEK-KMSQENLGKITDSIDQCKKDLADITSLEVKVKANYDS
PvivaxAM02_000474900.1     EEINNCLSNTKEYKEKCESEVNSVNRGKAKVDFLQKREALEK-KMSQENLGKITDSIDQCKKDLADITSLEVKVKANYDS
PVL_140006600-t42_1-p1     EEINTCLSNTKEYKEKCESEVNSVNRGKAKVDFLQKRKELEENRMSQENLSKITDSIDQCKKDLAEIASSELKVKANYDS

PVX_121920.1-p1            IIKYEESINTILNYSSILEYKTKLEIRKKEKTDLMTYINTENSAIQEKLLNLQKKLNQLNENTDYTKVGNDLNNAKSTKA
PVP01_1402400.1-p1         IIKYEESINTILNYSSILEYKTKLEIRKKEKTDLMTYINTENSAIQEKLLNLQKKLNQLNENTDYTKVGNDLNNAKSTKA
Psim2302_000007000.1       --------------------------------------------------------------------------------
Psim3636_000006300.1       --------------------------------------------------------------------------------
PsimAD002_000007200.1      --------------------------------------------------------------------------------
PsimAD005_000448800.1      --------------------------------------------------------------------------------
PsimAF28_000006200.1       --------------------------------------------------------------------------------
PsimAF33_000006700.1       --------------------------------------------------------------------------------
PsimAF34_000005900.1       --------------------------------------------------------------------------------
PvivaxAM01_000076800.1     IIKYEESINTILNYSSILEYKTKLEIRKKEKTDLMTYINTENSAIQEKLLNLQKKLNQLNENTDYTKVGNDLNNAKSTKA
PvivaxAM02_000474900.1     IIKYEESINTILNYSSILEYKTKLEIRKKEKTDLMTYINTENSAIQEKLLNLQKKLNQLNENTDYTKVGNDLNNAKSTKA
PVL_140006600-t42_1-p1     IIKYEESINTILNYSSILEYQTKLEIRKKEKTDLMNYINTENSAIQEKLLNLQKKLNQLNENTDYTKVGNDLNNAKSTKA

PVX_121920.1-p1            NVTIQYNLGRVKHQLENLSVIKQELEKVLSAATDLERDISKIADVTESNNLESLNGKEADYTKRIRSFNKLKQLVQEKAA
PVP01_1402400.1-p1         NVIIQYNLGRVKHQLENVSVIKQELEKVLSAATDLERDISKIADVTESNNLESLNGKEADYTKRIRSFNKLKQLVQEKAA
Psim2302_000007000.1       --------------------------------------------------------------------------------
Psim3636_000006300.1       --------------------------------------------------------------------------------
PsimAD002_000007200.1      --------------------------------------------------------------------------------
PsimAD005_000448800.1      --------------------------------------------------------------------------------
PsimAF28_000006200.1       --------------------------------------------------------------------------------
PsimAF33_000006700.1       --------------------------------------------------------------------------------
PsimAF34_000005900.1       --------------------------------------------------------------------------------
PvivaxAM01_000076800.1     NVTIQYNLGRVKHQLENVSVIKQELEKVLSAATDLERDISKIADVTESNNLESLNGKEADYTKRIRSFNKLKQLVQEKAA
PvivaxAM02_000474900.1     NVTIQYNLGRVKHQLENLSVIKQELEKVLSAATDLERDTSKIADVTESNNLESLNGKEADYTKRIRSFNKLKQLVQEKAA
PVL_140006600-t42_1-p1     NVTIQYNLGRVKHQLENVSVIKQELEKVLSAATDLEREVSKISDVTESNNLESLNGKEADYTKHIKSFNKLKLLVQEKAA

PVX_121920.1-p1            KVEEISSDTDNIEKELTEHKIIFEVGLTERLIEIVKNRKSYVDTTKELLNSSLNNFASLFNGLDLNGYNPKANLEMYTQK
PVP01_1402400.1-p1         KVEEISSDTDNIEKELTEHKIIFEVGLTERLIEIVKNRKSYVDTTKELLNSSLNNFASLFNGLDLKGYNPKANLEMYTQK
Psim2302_000007000.1       --------------------------------------------------------------------------------
Psim3636_000006300.1       --------------------------------------------------------------------------------
PsimAD002_000007200.1      --------------------------------------------------------------------------------
PsimAD005_000448800.1      --------------------------------------------------------------------------------
PsimAF28_000006200.1       --------------------------------------------------------------------------------
PsimAF33_000006700.1       --------------------------------------------------------------------------------
PsimAF34_000005900.1       --------------------------------------------------------------------------------
PvivaxAM01_000076800.1     KVEEISSDTDNIEKELTEHKIIFEVGLTERLIEIVKNRKSYVDTTKELLNSSLNNFASLFNGLDLNGYNPKANLEMYTQK
PvivaxAM02_000474900.1     KVEEISSDTDNIEKELTEHKIIFEVGLTERLIEIVKNRKSYVDTTKELLNSSLNNFASLFNGLDLNGYNPKANLEMYTQK
PVL_140006600-t42_1-p1     KVEEISSDTDNIEKELTEHKIIFEVGLTERLIEIIKNRKSYVDTTKELLNSSLNNFASLFNGLDLKGYNPKANLEMYTQK

PVX_121920.1-p1            LNTIHNEFMASHKIFDEKSKKVLDKDVNFTEAKTLREEAQKEDVTLKNKEEEAKSYLSDIKKKESFEFILHMKEKLNQIS
PVP01_1402400.1-p1         LNTIHNEFMASHKIFDEKSKKVLDKDVNFTEAKTLREEAQKEDVTLKNKEEEAKSYLSDIKKKESFEFILHMKEKLNQIS
Psim2302_000007000.1       --------------------------------------------------------------------------------
Psim3636_000006300.1       --------------------------------------------------------------------------------
PsimAD002_000007200.1      --------------------------------------------------------------------------------
PsimAD005_000448800.1      --------------------------------------------------------------------------------
PsimAF28_000006200.1       --------------------------------------------------------------------------------
PsimAF33_000006700.1       --------------------------------------------------------------------------------
PsimAF34_000005900.1       --------------------------------------------------------------------------------
PvivaxAM01_000076800.1     LNTIHNEFMASHKIFDEKSKKVLDKDVNFTEAKTLREEAQKEDVTLKNKEEEAKSYLSDIKKKESFEFILHMKEKLNQIS
PvivaxAM02_000474900.1     LNTIHNEFMASHKIFDEKSKKVLDKDVNFTEAKTLREEAQKEDVTLKNKEEEAKSYLSDIKKKESFEFILHMKEKLNQIS
PVL_140006600-t42_1-p1     LNTIHNEFMASHKIFDEKSKKVLVKDVNFTEAKTLREEAQKEDVTLKNKEEEAKSYLSDIKKKESFEFILHMKEKLTQIS

PVX_121920.1-p1            KMCEQQYEQADKGYSEVKTSIDRIANLNDENSIADVLKEANDKNEQVQNLTHYTYKNEAQNVLRHMAKSANFIGINLVTG
PVP01_1402400.1-p1         KMCEQQYEQADKGYSEVKTSIDRIANLNDENSIADVLKEANDKNEQVQNLTHYTYKNEAQNVLRHMAKSANFIGINLVTG
Psim2302_000007000.1       --------------------------------------------------------------------------------
Psim3636_000006300.1       --------------------------------------------------------------------------------
PsimAD002_000007200.1      --------------------------------------------------------------------------------
PsimAD005_000448800.1      --------------------------------------------------------------------------------
PsimAF28_000006200.1       --------------------------------------------------------------------------------
PsimAF33_000006700.1       --------------------------------------------------------------------------------
PsimAF34_000005900.1       --------------------------------------------------------------------------------
PvivaxAM01_000076800.1     KMCEQQYEQADKGYSEVKTSIDRIANLNDENSIADVLKEANDKNEQVQNLTHYTYKNEAQNVLRHMAKSANFIGINLVTG
PvivaxAM02_000474900.1     KMCEQQYEQADKGYSEVKTSIDRIANLNDENSIADVLKEANDKNEQVQNLTHYTYKNEAQNVLRHMAKSANFIGINLVTG
PVL_140006600-t42_1-p1     KMCEQQYEQADKGYSEVKTSIDRIANLNDENSVADVLKEANDQNEQVQNLTHYTYKNEAQNVLRHMAKSANFIGINLVTG
```

Figure S25 (cont.)

```
PVX_121920.1-p1             IQPTELSSQASESTTPELKFESEGEMKLEILTLSGNTTKLDYYKNMKDAYQSVLSIFKYSHGIDEKQRESQKITESANGS
PVP01_1402400.1-p1          IQPTELSSQASESTTPELKFESEGEMKLEILTLSGNTTKLDYYKNMKDAYQSVLSIFKYSHGIDEKQRESQKITESANGS
Psim2302_000007000.1        --------------------------------------------------------------------------------
Psim3636_000006300.1        --------------------------------------------------------------------------------
PsimAD002_000007200.1       --------------------------------------------------------------------------------
PsimAD005_000448800.1       --------------------------------------------------------------------------------
PsimAF28_000006200.1        --------------------------------------------------------------------------------
PsimAF33_000006700.1        --------------------------------------------------------------------------------
PsimAF34_000005900.1        --------------------------------------------------------------------------------
PvivaxAM01_000076800.1      IQPTELSSQASESTTPELKFESEGEMKLEILTLSGNTTKLDYYKNMKDAYQSVLSIFKYSHGIDEKQRESQKITESANGS
PvivaxAM02_000474900.1      IQPTELSSQASESTTPELKFESEGEMKLEILTLSGNTTKLDYYKNMKDAYQSVLSIFKYSHGIDEKQRESQKITESANGS
PVL_140006600-t42_1-p1      IQPTELSSQASESTTPELKFESEREMKLEILTLSGNTTKLDYYKNMKDAYQSVLSILKYSHGIDEKQRESQKITESANGS

PVX_121920.1-p1             YLNNKEINEFKGRLNNVKSKQTAISNKIDNATTLLHNLNKIKTDDKNYDTILEKDASEELKRRRDSFNQEMKNTVDGLKL
PVP01_1402400.1-p1          YLNNKEINEFKGRLNNVKSKQTAISNKIDNATTLLHNLNKIKTDDKNYDTILEKDASEELKRRRDSFNQEIKNTVDGLKL
Psim2302_000007000.1        --------------------------------------------------------------------------------
Psim3636_000006300.1        --------------------------------------------------------------------------------
PsimAD002_000007200.1       --------------------------------------------------------------------------------
PsimAD005_000448800.1       --------------------------------------------------------------------------------
PsimAF28_000006200.1        --------------------------------------------------------------------------------
PsimAF33_000006700.1        --------------------------------------------------------------------------------
PsimAF34_000005900.1        --------------------------------------------------------------------------------
PvivaxAM01_000076800.1      YLNNKEINEFKGRLNNVKSKQTAISNKIDNATTLLHNLNKIKTDDKNYDTILEKDASEELKRRRDSFNQEMKNTVDGLKL
PvivaxAM02_000474900.1      YLNNKEINEFKGRLNNVKSKQTAISNKIDNATTLLHNLNKIKTDDKNYDTILEKDASEELKRRRDSFNQEMKNTVDGLKL
PVL_140006600-t42_1-p1      YLNNKAINEFKGRLNNVKSKQTAISNKIDNATTLLHNLNKIKTDDKNYDTILEKDASEELKRRRDSFNQEMKNTVDGLKL

PVX_121920.1-p1             KEIQEKFNEQVKLLQNLETKVSTLNVHEGNATETVKKENTAVDAIQAAMEGIEKDVEYINYSYDELLKKGQKIENQRYTS
PVP01_1402400.1-p1          KEIQEKFNEQVKLLQNLETKVSTLNVHEGNATETVKKENTAVDAIQAAMEGIEKDVEYINYSYDELLKKGQKIENQRYTS
Psim2302_000007000.1        --------------------------------------------------------------------------------
Psim3636_000006300.1        --------------------------------------------------------------------------------
PsimAD002_000007200.1       --------------------------------------------------------------------------------
PsimAD005_000448800.1       --------------------------------------------------------------------------------
PsimAF28_000006200.1        --------------------------------------------------------------------------------
PsimAF33_000006700.1        --------------------------------------------------------------------------------
PsimAF34_000005900.1        --------------------------------------------------------------------------------
PvivaxAM01_000076800.1      KEIQEKFNEQVKLLQNLETKVSTLNVHEGNATETVKKENTAVDAIQAAMEGIEKDVEYINYSYDELLKKGQKIENQRYTS
PvivaxAM02_000474900.1      KEIQEKFNEQVKLLQNLETKVSTLNVHEGNATETVKKENTAVDAIQAAMEGIEKDVEYINYSYDELLKKGQKIENQRYTS
PVL_140006600-t42_1-p1      KEIQEKFNEQVKLLQNLETKVSTLNVHEGNVTETVKKENTAVDAIQAAMEGIEKDVEYINYSYDELLKKGQKIENQRYTS

PVX_121920.1-p1             IRENLTNKIANDSSAINKIKKKAQQYLAYIKNNYNSIYNDTGTLNEYFDTKRLSNHDLTNVQEATRLHIEMSAAVEASEE
PVP01_1402400.1-p1          IRENLTNKIANDSSAINKIKKKAQEYLAYIKNNYNSIYNDTGTLNEYFDTKRLSNHDLTNVQEATRLHIEMSAAVEASEE
Psim2302_000007000.1        --------------------------------------------------------------------------------
Psim3636_000006300.1        --------------------------------------------------------------------------------
PsimAD002_000007200.1       --------------------------------------------------------------------------------
PsimAD005_000448800.1       --------------------------------------------------------------------------------
PsimAF28_000006200.1        --------------------------------------------------------------------------------
PsimAF33_000006700.1        --------------------------------------------------------------------------------
PsimAF34_000005900.1        --------------------------------------------------------------------------------
PvivaxAM01_000076800.1      IRENLTNKIANDSSAINKIKEKAQQYLAYIKNNYNSIYNDTGTLNEYFDTKRLSNHDLTNVQEATRLHIEMSAAVEASEE
PvivaxAM02_000474900.1      IRENLTNKIANDSSAINKIKEKAQQYLAYIKNNYNSIYNDTGTLNEYFDTKRLSNHDLTNVQEATRLHIEMSAAVEASEE
PVL_140006600-t42_1-p1      IRENLTNKIANDSSAINKIKKKAQQYLAYIKNNYNSIYNDIGTLNEYFDIKRLSNHDLTNVQEATRLHIEMSAAVEASEE

PVX_121920.1-p1             IIADMKNEFITNTEADISALQNSADRLKSLYSNLKRKQISINQIYKKINLIKLQEIKTSANKYMDIAKLFNNVLEAQHKE
PVP01_1402400.1-p1          IIADMKNEFITNTEADISALQNSADRLMSLYSNLKRKQISINQIYKKINLIKLQEIKTSANKYMDIAKLFNNVLEAQHKE
Psim2302_000007000.1        --------------------------------------------------------------------------------
Psim3636_000006300.1        --------------------------------------------------------------------------------
PsimAD002_000007200.1       --------------------------------------------------------------------------------
PsimAD005_000448800.1       --------------------------------------------------------------------------------
PsimAF28_000006200.1        --------------------------------------------------------------------------------
PsimAF33_000006700.1        --------------------------------------------------------------------------------
PsimAF34_000005900.1        --------------------------------------------------------------------------------
PvivaxAM01_000076800.1      IIADMKNEFITNTEADISALQNSADRLKSLYSNLKRKQISINQIYKKINLIKLQEIKTSANKYMDIAKLFNNVLEAQHKE
PvivaxAM02_000474900.1      IIADMKNEFITNTEADISALQNSADRLKSLYSNLKRKQISINQIYKKINLIKLQEIKTSANKYMDIAKLFNNVLEAQHKE
PVL_140006600-t42_1-p1      IIADMKNEFITNTEADISALQNSADRLKSLYSNLKRKQISINQIYKKINLIKLQEIKTSANKYMDIAKLFNNVLEAQHKK

PVX_121920.1-p1             LAQDRSKILQAK--------------------------------------------------------------------
PVP01_1402400.1-p1          LAQDISKILQAK--------------------------------------------------------------------
Psim2302_000007000.1        --------------------------------------------------------------------------------
Psim3636_000006300.1        --------------------------------------------------------------------------------
PsimAD002_000007200.1       --------------------------------------------------------------------------------
PsimAD005_000448800.1       --------------------------------------------------------------------------------
PsimAF28_000006200.1        --------------------------------------------------------------------------------
PsimAF33_000006700.1        --------------------------------------------------------------------------------
PsimAF34_000005900.1        --------------------------------------------------------------------------------
PvivaxAM01_000076800.1      LAQDRSKILQAK--------------------------------------------------------------------
PvivaxAM02_000474900.1      LAQDRSKILQAK--------------------------------------------------------------------
PVL_140006600-t42_1-p1      LAQDRSKILQVKEKLINTEKELANLDETITLQSLKKSNELCNSATKNIQDIRELEKENNKEDKKIKIYGEKISHLINRRK
```

Figure S25 (cont.)

```
PVX_121920.1-p1             --------------------------------------------------------------------------------
PVP01_1402400.1-p1          --------------------------------------------------------------------------------
Psim2302_000007000.1        --------------------------------------------------------------------------------
Psim3636_000006300.1        --------------------------------------------------------------------------------
PsimAD002_000007200.1       --------------------------------------------------------------------------------
PsimAD005_000448800.1       --------------------------------------------------------------------------------
PsimAF28_000006200.1        --------------------------------------------------------------------------------
PsimAF33_000006700.1        --------------------------------------------------------------------------------
PsimAF34_000005900.1        --------------------------------------------------------------------------------
PvivaxAM01_000076800.1      --------------------------------------------------------------------------------
PvivaxAM02_000474900.1      --------------------------------------------------------------------------------
PVL_140006600-t42_1-p1      VLLNDVSEYDRTENFDHENEQAANDLQNDIATIKKVLVSSEEQYRKLLENVKKNESLYSNNDTKNFTLEISKKIENVKRK

PVX_121920.1-p1             --------------------------------------------------------------------------------
PVP01_1402400.1-p1          --------------------------------------------------------------------------------
Psim2302_000007000.1        --------------------------------------------------------------------------------
Psim3636_000006300.1        --------------------------------------------------------------------------------
PsimAD002_000007200.1       --------------------------------------------------------------------------------
PsimAD005_000448800.1       --------------------------------------------------------------------------------
PsimAF28_000006200.1        --------------------------------------------------------------------------------
PsimAF33_000006700.1        --------------------------------------------------------------------------------
PsimAF34_000005900.1        --------------------------------------------------------------------------------
PvivaxAM01_000076800.1      --------------------------------------------------------------------------------
PvivaxAM02_000474900.1      --------------------------------------------------------------------------------
PVL_140006600-t42_1-p1      ISINIPESEQLLQIENRFGDIKAIINGIKADNDVDEYVEEVYKNIQREKEKLTDMRNGEQVKEAIKNITHYNDETKNKLS

PVX_121920.1-p1             ----------------------------------------ENSINMQQLLESHIDKLRALITNIDKELIELTNGKIKESN
PVP01_1402400.1-p1          ----------------------------------------ENSINMQQLLESHIDKLRALITNIDKELIELTNGKIKASN
Psim2302_000007000.1        --------------------------------------------------------------------------------
Psim3636_000006300.1        --------------------------------------------------------------------------------
PsimAD002_000007200.1       --------------------------------------------------------------------------------
PsimAD005_000448800.1       --------------------------------------------------------------------------------
PsimAF28_000006200.1        --------------------------------------------------------------------------------
PsimAF33_000006700.1        --------------------------------------------------------------------------------
PsimAF34_000005900.1        --------------------------------------------------------------------------------
PvivaxAM01_000076800.1      ----------------------------------------ENSINMQQLLESHIDKLRALITNIDKELIELTNDKIKASN
PvivaxAM02_000474900.1      ----------------------------------------ENSINMQQLLESHIDKLRALITNIDKELIELTNGKIKESN
PVL_140006600-t42_1-p1      RIYNAFEKVKMKKKDMEKIFASISEKSENNAIQNDVKNAIEHSINMQQLLESHIDKLRALITNIDKELIELKNGKIKASN

PVX_121920.1-p1             RRISQISPMGQGKLFSTPEGQAYNNLHNTGYNHYGSGNHSRGRNENGNVRFAAGIVVGFGVCSFFASALFKGKGENETYG
PVP01_1402400.1-p1          RRISQISPMGQGKLFSTPEGQAYNNLHNTGYNHNGSGNHSRGRNENGNVRFAAGIVVGFGVCSFFASALFKGKGENETYG
Psim2302_000007000.1        --ISQISPMEQGKLFSTPEGQAYNNLHNTGYNHYGSGNHSRGRNENGNVRFAAGIVVVFGVCSFFASALFKGKGENETYG
Psim3636_000006300.1        --ISQISPMEQGKLFSTPEGQAYNNLHNTGYNHYGSGNHSRGRNENGNVRFAAGIVVVFGVCSFFASALFKGKGENETYG
PsimAD002_000007200.1       --ISQISPMEQGKLFSTPEGQAYNNLHNTGYNHYGSGNHSRGRNENGNVRFAAGIVVVFGVCSFFASALFKGKGENETYG
PsimAD005_000448800.1       --ISQISPMEQGKLFSTPEGQAYNNLHNTGYNHYGSGNHSRGRNENGNVRFAAGIVVVFGVCSFFASALFKGKGENETYG
PsimAF28_000006200.1        --ISQISPMEQGKLFSTPEGQAYNNLHNTGYNHYGSGNHSRGRNENGNVRFAAGIVVVFGVCSFFASALFKGKGENETYG
PsimAF33_000006700.1        --ISQISPMEQGKLFSTPEGQAYNNLHNTGYNHYGSGNHSRGRNENGNVRFAAGIVVVFGVCSFFASALFKGKGENETYG
PsimAF34_000005900.1        --ISQISPMEQGKLFSTPEGQAYNNLHNTGYNHYGSGNHSRGRNENGNVRFAAGIVVVFGVCSFFASALFKGKGENETYG
PvivaxAM01_000076800.1      RRISQISPMGQGKLFSTPEGQAYNNLHNTGYNHYGSGNHSRGRNENGNVRFAAGIVVGFGVCSFFASALFKGKGENETYG
PvivaxAM02_000474900.1      RRISQISPMGQGKLFSTPEGQAYNNLHNTGYNHYGSGNHSRGRNENGNVRFAAGIVVGFGVCSFFASALFKGKGENETYG
PVL_140006600-t42_1-p1      RRISQISPMGQGKLFSTPEGQAYNNLHNTGYNHYGSGNHSRGRNENGNVRFAAGIVVGLGVCSFFASALFKGKKENETYG

PVX_121920.1-p1             RDLNSRDEEFEGKNNGNLQDKEEIIEVSFHESENVY
PVP01_1402400.1-p1          RDLNSRDEEFEGKNNGNLQDKEEIIEVSFHESENAY
Psim2302_000007000.1        RDLNSRDEEFEGKNNGNLQDKEEIIEVSFHESENVY
Psim3636_000006300.1        RDLNSRDDEFEGKNNGNLQDKEEIIEVSFHESENVY
PsimAD002_000007200.1       RDLNSRDDEFEGKNNGNLQDKEEIIEVSFHESENVY
PsimAD005_000448800.1       RDLNSRDDEFEGKNNGNLQDKEEIIEVSFHESENVY
PsimAF28_000006200.1        RDLNSRDDEFEGKNNGNLQDKEEIIEVSFHESENVY
PsimAF33_000006700.1        RDLNSRDEEFEGKNNGNLQDKEEIIEVSFHESENVY
PsimAF34_000005900.1        RDLNSRDEEFEGKNNGNLQDKEEIIEVSFHESENVY
PvivaxAM01_000076800.1      RDLNSRDEEFEGKNNGNLQDKEEIIEVSFHESENVY
PvivaxAM02_000474900.1      RDLNSRDEEFEGKNNGNLQDKEEIIEVSFHESENVY
PVL_140006600-t42_1-p1      RDLNSRDEEFEGKNNGNLQDKEEIIEVSFHESENVY
```
